# Conformational Dynamics and Binding Interactions of SARS-CoV‑2 Spike Protein Variants: Omicron, XBB.1.9.2, and EG.5

**DOI:** 10.1021/acs.jcim.5c00308

**Published:** 2025-07-11

**Authors:** Clauber Henrique Souza da Costa, Camila Auad Beltrão de Freitas, Alberto Monteiro dos Santos, Carlos Gabriel da Silva de Souza, José Rogério A. Silva, Jerônimo Lameira, Vicent Moliner, Munir S. Skaf

**Affiliations:** 1 Institute of Chemistry and Center for Computing in Engineering & Sciences, 28132 University of Campinas − UNICAMP, Campinas, SP 13084-862, Brazil; 2 Laboratório de Planejamento e Desenvolvimento de Fármacos, Instituto de Ciências Exatas e Naturais, 37871 Universidade Federal do Pará, Belém, Pará 66075-110, Brazil; 3 Laboratory of Computer Modeling of Molecular Biosystems (CompMBio), Federal University of Pará, Belém 66075-110, Brazil; 4 Catalysis and Peptide Research Unit, University of KwaZulu-Natal, Durban 4000, South Africa; 5 Institute of Advanced Materials (INAM), 16748 Universitat Jaume I, Castellon 12071, Spain

## Abstract

The SARS-CoV-2 virus, responsible for the COVID-19 pandemic, has continuously evolved, generating numerous variants with varying degrees of infectivity and transmissibility. The EG.5 subvariant of SARS-CoV-2 emerged globally in mid-2023 as part of the ongoing evolution of the Omicron lineage. Derived from the recombinant XBB.1.9 sublineage, EG.5 has attracted attention due to its enhanced immune escape and sustained transmissibility. As a member of the FLip lineage, EG.5 harbors the convergent F456L mutation in the spike receptor-binding domain (RBD), a key residue for neutralizing antibody recognition. Understanding the molecular mechanisms underlying these variations is crucial for developing effective antiviral strategies. In this study, we employed accelerated molecular dynamics simulations, free-energy calculations, and interaction fingerprint analysis, to investigate the intricate molecular interactions between the spike RBD and the angiotensin-converting enzyme 2 (ACE2) receptor in wild-type SARS-CoV-2 and its variants, specifically Omicron, XBB.1.9.2, and the concerning EG.5 variant. Our findings reveal that electrostatic interactions are the predominant driving force behind the stabilization of the viral spike protein-ACE2 complex. The Omicron, XBB.1.9.2, and EG.5 variants exhibit distinct electrostatic profiles at the spike–ACE2 interface, with mutations at key residues reconfiguring local interactions. These changes enhance ACE2 binding specificity and stabilize the spike–ACE2 complex through intensified electrostatic interactions. The EG.5 variant, with its stronger binding affinity to ACE2, underscores the ongoing threat posed by SARS-CoV-2. The F456L mutation in EG.5 enhances protein stability, further supporting its increased affinity for ACE2. Our research provides valuable insights for designing targeted antiviral therapies, including peptide inhibitors and bioactive compounds. Continuous research is essential to effectively combat COVID-19 and its evolving variants.

## Graphical abstract

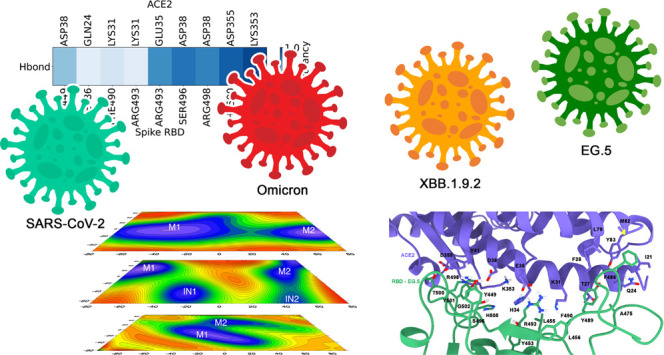

## Introduction

Since the onset of the COVID-19 pandemic in early 2020, the SARS-CoV-2 (severe acute respiratory syndrome coronavirus 2) virus has posed a significant global health challenge.^1,2^ Originating in Wuhan, China, this highly infectious virus has rapidly spread worldwide, undergoing continuous genetic mutations.^3–6^

The Omicron variant (B.1.1.529), which emerged in late 2021, was the last Variant of Interest (VOI) designated by the World Health Organization (WHO) until August 2023. Previous variants, such as Alpha (B.1.1.7), Beta (B.1.351), Gamma (P.1), and Delta (B.1.617.2), exhibited distinct impacts on transmissibility, disease severity, and vaccine response.^7^ First reported in February 2023, the EG.5 variant rapidly reached high global prevalence, with over 100,000 sequences submitted to the Global Initiative on Sharing All Influenza Data (GISAID) database by November 2023.^8,9^

Significant advances have been made in understanding SARS-CoV-2, particularly by clarifying that dynamic interactions between the wild-type (WT) spike protein, a class I homotrimeric fusion glycoprotein composed of 1,273 amino acids,^3,10,11^ and its variants are pivotal for viral entry through recognition of the angiotensin-converting enzyme 2 (ACE2) receptor.^10,11^ This interaction is governed by the receptor-binding domain (RBD), a region that undergoes conformational changes to expose or conceal receptor-binding determinants. Notably, ACE2 is the primary receptor for spike protein–host cell engagement, as the viral entry process relies on the RBD within the S1 subunit of the spike protein interacting with ACE2.^8,12–14^

The Omicron variant, presented a significant number of mutations, including K417N, S477N, T478 K, E484A, and N501Y in its spike protein.^9^ Clinically, this variant exhibited a shorter incubation period, milder symptoms, and an increased risk of reinfection compared to previous SARS-CoV-2 variants.^15,16^ Initially, BA.1, BA.1.1, and BA.2 were the most prevalent sublineages. However, by February 2023, XBB subvariants, particularly XBB.1.5, became dominant globally.^17^ Subsequently, additional XBB sublineages like XBB.1.9.1, XBB.1.9.2, XBB.1.16, and XBB.2.3 emerged. These sublineages accumulated spike protein mutations such as F486P, R403 K, V445S, L455F, F456L, and K478R, potentially leading to increased antigenic drift and immune escape, posing challenges to vaccine efficacy and natural immunity.^18,19^

EG.5, also known as Eris, is a descendant of XBB.1.9.2, sharing a similar amino acid profile with XBB.1.5 (Kraken), which contributes to its increased transmissibility and immune evasion.^20–23^ EG.5 carries an additional F456L mutation in the spike protein, while its immediate descendant EG.5.1 has an additional Q52H mutation.^24–26^ Both were found to be moderately more resistant for class one monoclonal antibodies, confirmed in studies of immune evasion, which showed that F456L mutation drives the enhanced neutralization escape.^27–29^

EG.5.1 has further evolved, resulting in a descendant lineage named HK.3 (XBB.1.9.2.5.1.1.3), which harbors L455F mutation,^29^ ushering in a new generation of spike protein mutations known as FLip mutations (L455F and F456L), which involve the exchange of two amino acids, can increase ACE2 binding and further reduce neutralizing antibody efficacy,^30,31^ suggesting that it may outcompete other circulating XBB subvariants and become the dominant strain globally.^32,33^

Experimentally, was reported that these mutations act synergistically to promote greater evasion of class one neutralizing antibodies, since residues 455 and 456 in the RBD gene are predominantly recognized by these antibodies.^27^ This corroborates structural analyses, which indicate that the disruption in the receptor binding mode results in an exceptional affinity for ACE2, reinforcing the ability to escape the action of antibodies.^34^ Collectively, the biochemical evidence indicates that the isolated L455F substitution diminishes the spike protein’s affinity for ACE2, whereas the concerted L455F and F456L residue shift enhances ACE2 engagement and markedly heightens resistance to class one monoclonal antibodies.^27,35^

Understanding the structural dynamics of viral interactions is critical to guiding infection control strategies, elucidating the evolution of viral machinery, and supporting vaccine development in the face of emerging, more transmissible variants.^8,12,14,36–40^ Given the continuous genetic evolution of SARS-CoV-2, this study employed accelerated molecular dynamics (aMD) simulations, combined with Free Energy calculations and Interaction Fingerprint Analysis (IFP), in an attempt to investigate the impact of specific RBD mutations on the interaction between the viral spike protein and the human ACE2 receptor. We compared the wild-type (WT) virus with the Omicron variant and its subvariants XBB.1.9.2 and EG.5, aiming to identify structural dynamics, binding affinity changes, and potential correlations with increased infectivity and viral evolution. Our results are discussed in the light of available experimental findings inasmuch as possible.

## Material and Methods

We constructed molecular dynamics simulation systems based on the RBD-ACE2 complex structure (PDB code 6M0J).^12^ Our previous work investigated structural differences between the WT SARS-CoV-2 RBD and the RBDs of the Omicron, XBB.1.9.2, and EG.5 variants (Figure 1).^41^ The Omicron variant served as a template for the XBB.1.9.2 system, incorporating the S371F, T376A, D405N, R408S, and N501Y mutations. Similarly, the EG.5 system included the F456L mutation.^25^ The Q52H mutation, present in EG.5.1, was not explicitly modeled but can be inferred from our results, as this variant also carries the F456L mutation. Protonation states were assigned using the PDB 2PQR server at pH 7.^42^ The systems were modeled using the AMBER22 package with the ff14SB force field^43,44^ and solvated with TIP3P water^45^ in a cubic box with a 10 Å buffer. Sodium ions were added for electroneutrality.

**Figure 1:**
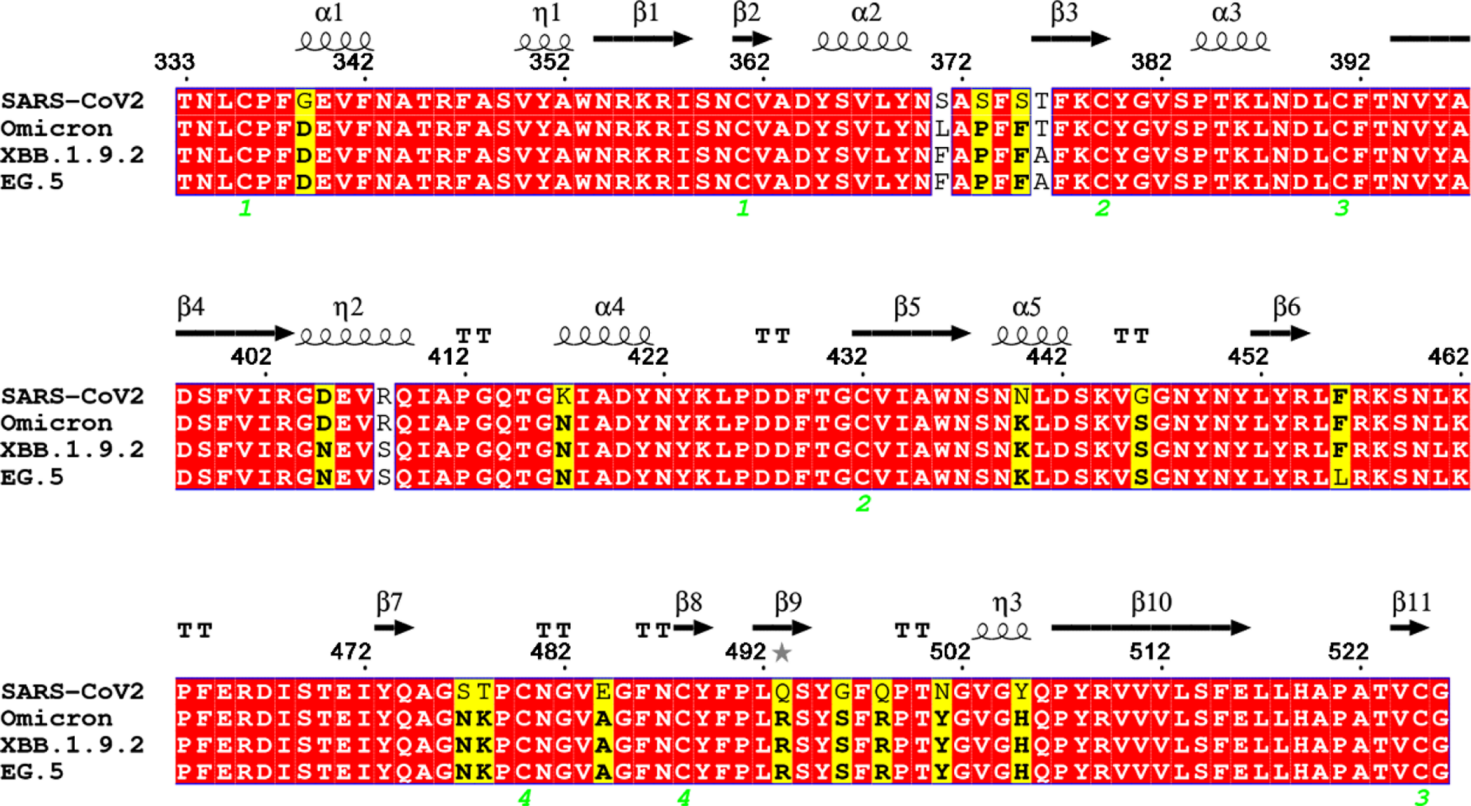
Alignment of the sequences of models of WT SARS-CoV-2 and variants Omicron, XBB.1.9.2, and EG.5 used in this work.

The protocol preceding the aMD simulations was consistent with our previous work.^41^ It involved four minimization stages (10,000 cycles each) using the conjugate gradient method and steep descent to minimize energy and optimize the system. The system was then heated linearly from 0 to 300 K over 5 ns under NVT conditions using Langevin dynamics (collision frequency of 2 ps) as a thermostat. The SHAKE algorithm was employed to constrain covalent hydrogen bonds.^46^ Subsequently, 10 ns of classical molecular dynamics (MD) simulations were performed to serve as a reference potential for the subsequent aMD simulations. Finally, three independent 200 ns aMD simulations were conducted for each system (RBD-SARS-CoV-2-ACE2, RBD-Omicron-ACE2, RBD-XBB.1.9.2-ACE2, and RBD-EG.5-ACE2) under NPT conditions, each initiated with different random seeds to ensure statistical independence and adequate sampling.

The use of artificial potentials to smooth the energy surface in accelerated molecular dynamics (aMD) simulations has been widely employed to explore rare events and relevant conformational transitions.^41,47,48^ However, its effectiveness is highly sensitive to the definition of boost parameters, and suboptimal choices can compromise the representativeness of conformational sampling.^47,49^ Additionally, modifying the potential energy surface may introduce artifacts that affect the accuracy of thermodynamic and kinetic property estimations.^48^ While these limitations do not preclude the method’s applicability, they must be carefully considered when interpreting quantitative results and constructing free energy surfaces derived from such simulations.^48–50^ In this study, aMD was selected for its ability to overcome energy barriers and sample sparsely populated conformational states, which is particularly advantageous for investigating protein–protein interactions modulated by point mutations. This approach has previously proven effective in studies involving SARS-CoV-2 variants (Alpha, Delta, and Omicron), enabling access to relevant conformational regions associated with binding affinity and potential immune escape mechanisms, thereby providing insights into differential transmissibility among variants.^41^

The successful application of aMD enabled us to elucidate the differences between the variant, its subvariants, and the WT, allowing for exploration of a wider range of low-energy structures. By reducing energy barriers between different system states, aMD overcomes the temporal limitations inherent in traditional nanosecond simulations, which are often confined to potential energy minima with high energy barriers during many computational steps.^41,51–56^

The initial step in aMD involved calculating the total potential (EPTOT) and dihedral angle energies (DIHED) during a 10 ns classical molecular dynamics (MD) simulation (Table S1). These parameters were found to stabilize during the MD simulation, as shown in Figure S1 and Table S1. These values were used to define the parameters required for aMD simulations.

### Principal Component Analysis (PCA) and Free Energy Landscape (FEL)

The CPPTRAJ module^44,57^ of AMBER22 was used to extract Cα atom trajectories from the MD simulations. Principal component analysis (PCA) was then performed on these trajectories. To ensure accurate PCA, an iterative superposition procedure was applied to align the structures, excluding residues with significant positional differences. This approach allows for a more reliable identification of the ″core″ invariant residues and prevents underestimation of atomic displacements.^58–61^

Free Energy Landscape (FEL) analysis provides a statistical mechanics description of a protein’s potential energy surface, revealing the most probable conformations.^62,63^ FEL analysis considers a broader range of conformations, highlighting the pathways leading to the functionally active native state.^62,64–66^ In this work, the construction of the free energy landscape was carried out using the principal components PC1 and PC2 in eq 1:

$$\Delta G(PC_{s})=-k_{B}T[\ln\;\rho (PC_{1},PC_{2})-\ln\;\rho_{\max}]$$ 1

As such, Δ*G*(*PC*_*s*_) is a function of the probability distribution obtained from the MD trajectories.^67,68^ The probability of the maximum value, ρ_*max*_, is subtracted from the free energy, scaled by the Boltzmann constant and temperature (300 K), to bring the lowest energy state closer to zero. The highest probability conformations, corresponding to the minima on the free energy landscape, denoted here M1 and M2, represent the most stable structures, potentially resembling the native state. Transition structures between M1 and M2 may not be easily identified. However, intermediate (IN) structures may emerge from local minima in the vicinity of the lowest points on the FEL (M1 and M2).

### Protein–Protein Binding Free Energy

To assess the binding affinities of the complexes, we employed the Molecular Mechanics Generalized Born Surface Area (MM/GBSA)^69^ method as implemented in AMBER22.^44^ This approach combines molecular mechanics calculations with a continuum solvation model to estimate binding free energies of macromolecules.^79–81^

Equations 2–4 define MM/GBSA or its Poisson–Boltzmann counterpart (MM/PBSA) and the binding free energy (Δ*G*_*bind*_) between a ligand (L) and a receptor (R) to form an RL complex:

$$\Delta G_{bind}=\Delta H-T\Delta S\approx \Delta E_{MM}+\Delta G_{sol}-T\Delta S$$ 2

$$\Delta E_{MM}=\Delta E_{inter}+\Delta E_{elec}+\Delta E_{vdw}$$ 3

$$\Delta G_{sol}=\Delta G_{PB/GB}+\Delta G_{SA}$$ 4

where Δ*E*_*MM*_, Δ*G*_*sol*_ and – *T*Δ*S* are the variations in MM energy from the gas phase, free solvation energy, and conformational entropy in the existing binding, respectively. The term Δ*E*_*MM*_ includes Δ*E*_*inter*_ (bond, angle, and dihedral energies), electrostatic energy (Δ*E*_*elec*_), and van der Waals energies (Δ*E*_*vdw*_). The Δ*G*_*sol*_ is the sum of the electrostatic solvation energies Δ*G*_*PB*/*GB*_ (polar contribution) with the nonelectrostatic solvation component Δ*G*_*SA*_ (nonpolar contribution). The polar contribution is calculated using the GB or PB models, while the nonpolar energy is estimated from the Solvent Accessible Surface Area (SASA). The change in conformational entropy – *T*Δ*S* is typically calculated by normal-mode analysis on a set of conformational snapshots obtained from MD simulations.^70–72^ A total of 25,000 frames were selected from 50 ns of aMD output per replica, amounting to 150 ns per system, usingΔG_bind_ = G_RBD‑ACE2_ - G_RBD_ – G_ACE2_5

Here, G_RBD‑ACE2_ represents the average of single-trajectory snapshots from the aMD simulation of the RBD-ACE2 complex. In turn, G_RBD_ and G_ACE2_ correspond to the free energy of the RBD and ACE2 proteins, respectively. This protocol was used in a previous study,^55^ in which we reported accurate results in describing the energies involved in the binding process between Spike RBD and ACE2.

### Interaction Fingerprints (IFP)

Inter- and intramolecular interactions are fundamental to molecular recognition and biochemical processes. Understanding these interactions provides insights into how key amino acid residues interact with ligands and how proteins interact with each other.^73,74^ Interaction fingerprints (IFPs) are vector representations of molecular interactions which can be used to identify interacting residues, characterize interaction types, and predict protein–ligand and protein–protein interactions.^74–77^ Here, the freely available ProLIF Python library has been used to generate functional interaction profiles from molecular complexes derived from molecular dynamics trajectories. ProLIF’s capabilities are enhanced by its integration with RDKit and MDAnalysis libraries,^73,78,79^ enabling comprehensive analysis of molecular interactions in various contexts.

## Results and Discussion

We performed three independent 200 ns aMD simulations for each of the four RBD–ACE2 complexes (WT, Omicron, XBB.1.9.2, and EG.5). This yielded a total of 600 ns of aMD sampling per system, the equivalent of 1.2 ms of conventional MD simulation.^41,80,81^

Our RMSD analysis reveals that the SARS-CoV-2 RBD exhibits distinct dynamic behavior compared to its variants (Figure 2a). The WT, Omicron, XBB.1.9.2, and EG.5 RBD domains displayed RMSD values of 3.17, 2.73, 2.34, and 1.94 Å, respectively, indicating a progressive stabilization of the RBD domain (Table S2). Notably, the XBB.1.9.2 replicas exhibited higher RMSD values for both the RBD and ACE2, reinforcing the trend of increased structural fluctuations for this variant (Table S2). A similar trend is observed for the RBD-ACE2 complexes. The WT, Omicron, and XBB.1.9.2 complexes exhibited greater RMSD values compared to the EG.5 complex (Figure 2c), suggesting more pronounced structural fluctuations and a wider range of conformational changes. Furthermore, analysis of the center-of-mass distance between the RBD and ACE2 revealed significant fluctuations for the XBB.1.9.2 variant, indicating potential alterations in the binding mode between these proteins during molecular dynamics simulations (Figure 2d). Additionally, the XBB.1.9.2 replicas showed greater center-of-mass distances between the RBD and ACE2, aligning with these observations. Our results highlight the unique stability of the EG.5 variant and suggest that XBB.1.9.2 may represent a transient phase in viral evolution. This interpretation is reinforced by the replica simulations, which showed that XBB.1.9.2 exhibited higher average RMSD values and greater RBD–ACE2 distances (Figure S3 and Table S2).

**Figure 2:**
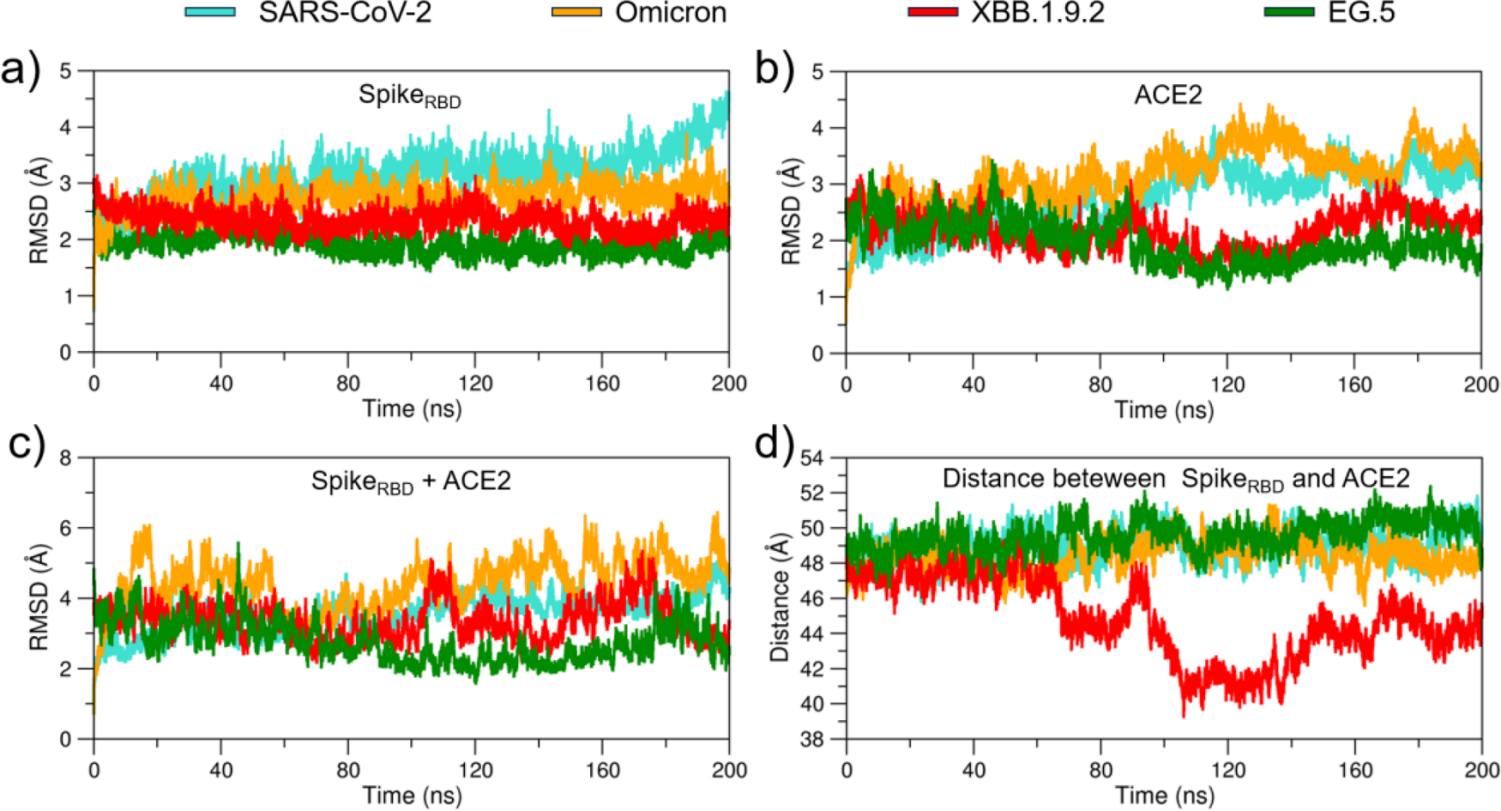
RMSD analysis for WT, Omicron, XBB.1.9.2, and EG.5. (a) RMSD for the RBD system. (b) RMSD for the ACE2 receptor. (c) RMSD for the complex formed by RBD + ACE2. (d) Distance between RBD and ACE2 for all systems.

The root-mean-square fluctuations (RMSF) of the RBD-ACE2 complex, depicted in Figure 3, highlight specific regions with significant conformational fluctuations. ACE2 residues are color-coded: pink (20–95), yellow (348–368), and blue (395–425). Similarly, Spike RBD regions are categorized as N-terminal (purple, 333–400), central (red, 401–452), and C-terminal (green, 453–536). Notably, the N-terminal RBD region (purple) exhibits the highest fluctuations, particularly in XBB.1.9.2, reaching up to 12 Å in certain positions. Conversely, the red and green RBD regions, directly involved in ACE2 interaction, display reduced fluctuations. In ACE2, the yellow and blue regions, which interact with the RBD, show significant fluctuations, although less pronounced in the XBB.1.9.2, Omicron, and EG.5 systems, suggesting that mutations in these regions may influence protein dynamics and binding interactions. The RMSF data for replicas 2 and 3 are presented in Figure S3, compared with replica 1, and demonstrate higher fluctuations in the RBD region of XBB.1.9.2, along with greater stability for the EG.5 fluctuations.

**Figure 3:**
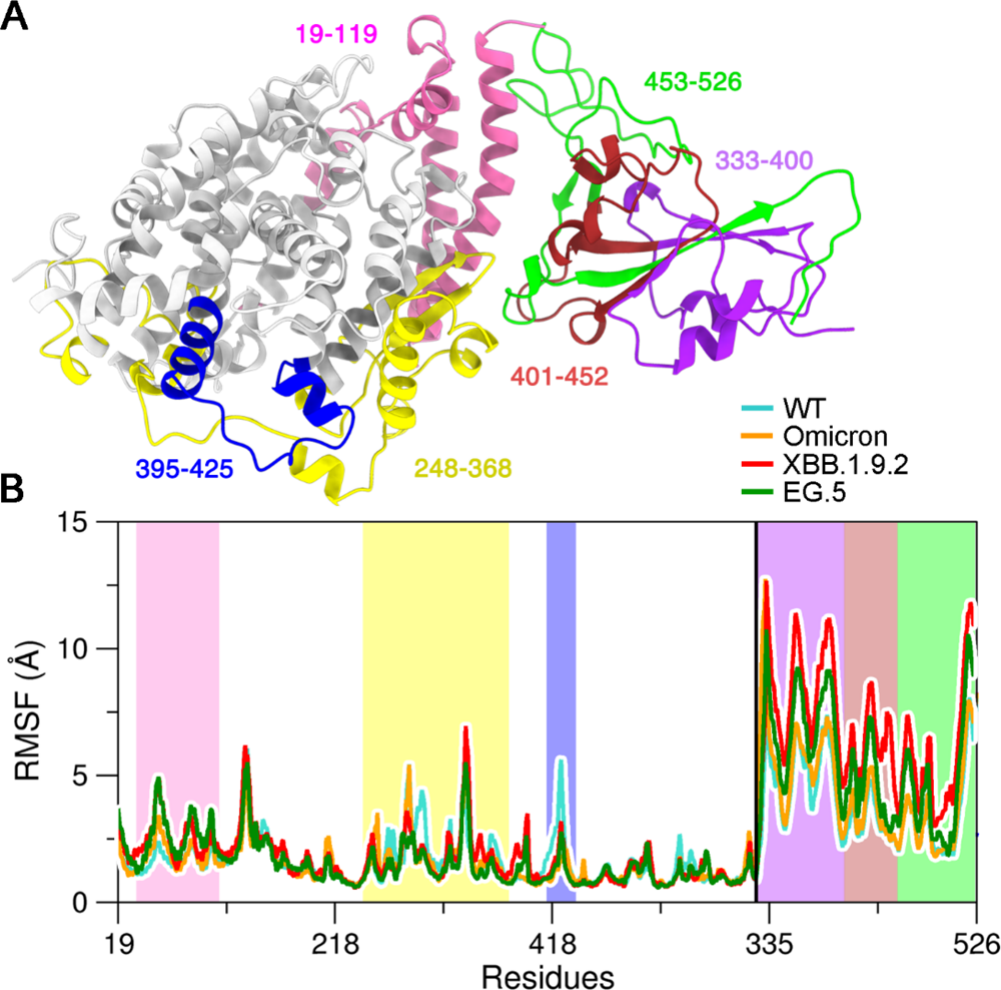
(a) Three-dimensional structure of ACE2 and RBD; (b) RMSF for different regions of WT SARS-CoV-2 (cyan), Omicron (orange), XBB.1.9.2 (red) and EG.5 (green) systems.

The Free Energy Landscape (FEL) was computed to further elucidate the relationship between fluctuations and conformational states of the RBD protein in the WT and its mutant variants. This analysis highlights the minimal (M) and intermediate (IN) conformational states adopted by the RBD along the MD trajectory. Representative M1 structures for the WT SARS-CoV-2 and its variants are presented in Figure 4, with additional structures provided in the Supporting Information (Figures S5–S8).

**Figure 4:**
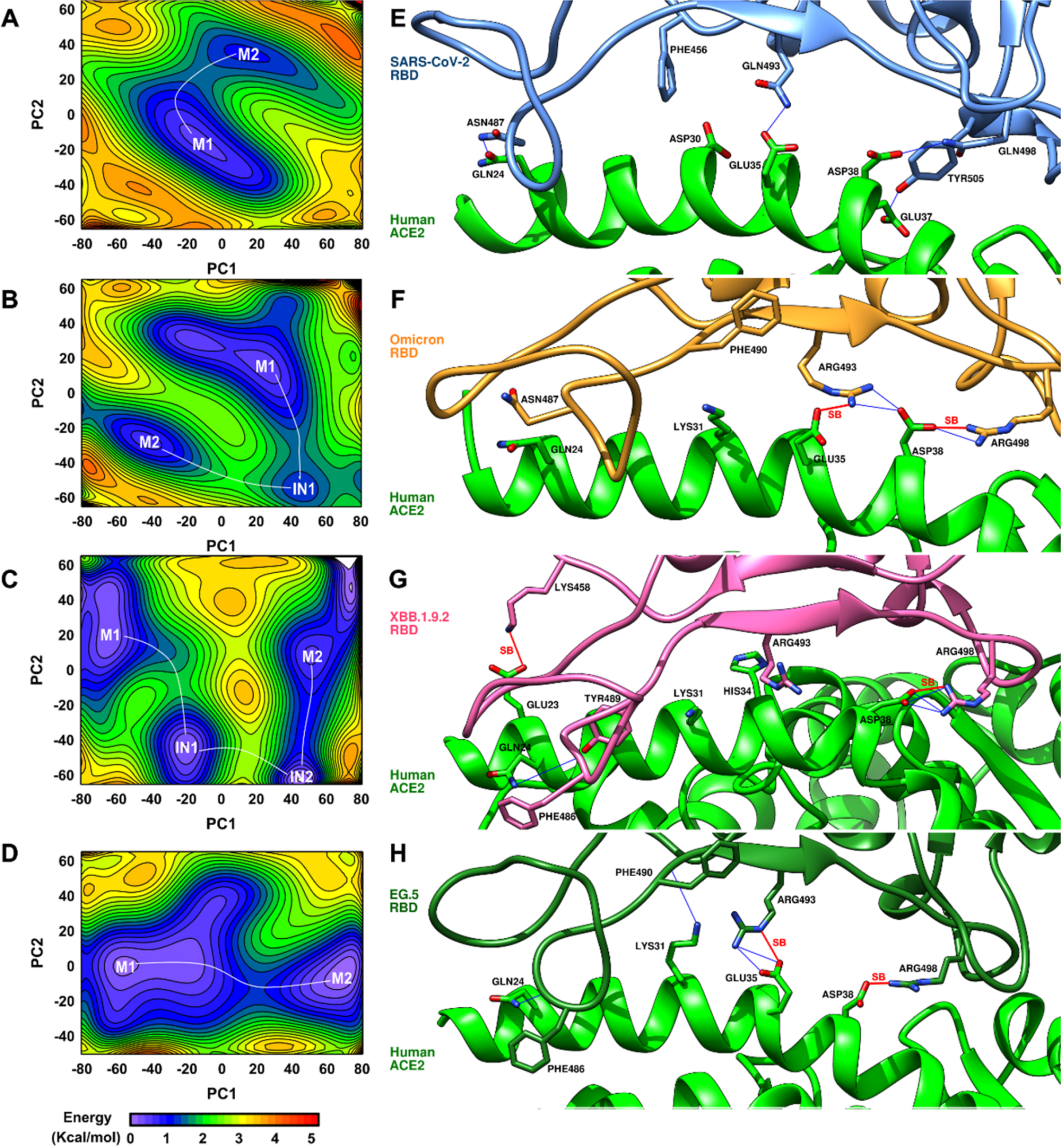
FEL analysis of native systems (a) SARS-CoV-2, (b) Omicron, (c) XBB.1.9.1, and (d) EG.5. The structures of each representative of the minima highlighting the distances that exist between the salt bridges (SB) are highlighted in red, (e) M1 SARS-CoV-2, (f) M1 Omicron, (g) M1 XBB.1.9.1, and (h) M1 EG.5, where minimum 1 (M1) and minimum 2 (M2) are two distinct conformational states. IN1 and IN2 are intermediate states.

In the SARS-CoV-2 system, two distinct conformational states, M1 and M2, are observed in the RBD (Figure 4a). The Omicron variant exhibits two primary conformational states, M1 and M2, connected by an intermediate state (IN1) characterized by rearrangements within the Spike RBD region (Figure 4b). In contrast, the Omicron subvariant XBB.1.9.2 demonstrates a more complex transition pathway between M1 and M2, involving two distinct intermediate states (IN1 and IN2) (Figure 4c). The EG.5 variant exhibits two stable minima characterized by strong ACE2 interactions and pronounced conformational changes within the Spike protein (Figure 4d). Furthermore, the proximity of the identified minima in all systems, particularly in the wild type and EG.5 variant, suggests a high degree of conformational plasticity within the spike protein. This dynamic switching between closely related binding modes may represent a potential mechanism for evading the immune response^85^ (Figure 4a,d) and may explain why XBB.1.9.2 is resistant to antiviral humoral immunity induced by vaccination.^82^

Stable salt bridge formations between the ACE2 receptor and the virus’ RBD significantly contribute to the binding stability of the complex (Figure 4f–h). Salt bridges play a crucial role in protein–protein interactions, influencing binding affinity and specificity. Their presence or absence can significantly impact the stability of the virus-receptor complex.^12,41,83,84^

The exclusive presence of the Asp30-Lys417 salt bridge in the WT SARS-CoV-2 system within the M2 basin (Figure S5a) suggests enhanced binding affinity compared to the M1 configuration, which lacks this interaction (Figure S5b) and likely exhibits a dynamic equilibrium between higher and lower affinity states. Notably, this particular salt bridge is absent in all variants considered here. Despite variations in domain interactions and conformations across the variants, the overall number of residues involved in the interface formation remains relatively constant (see Supporting Information).

Variant simulations reveal a notable shift toward a balanced distribution of hydrogen bonds and salt bridges (Figure 4f–h). Configurations with two or three salt bridges between ACE2 and the viral RBD exhibit the most favorable energetics. These stabilizing salt bridges, absent in the WT, emerge in the Omicron variant due to the Gln493Arg and Gln498Arg mutations, which are conserved in subsequent variants (Figure 4f–h).

Transitions between different minima were also observed in the other replicas, highlighting that WT exhibits two main conformations throughout the aMD simulations. Similarly, XBB.1.9.2 undergoes frequent transitions between minima, whereas the EG.5 variant displays greater stability among its primary conformational states (Figure S9).

Analysis of the variants reveals a shifting balance between hydrogen bonds, salt bridges, and nonbonded interactions. This suggests an evolutionary trend toward exploiting dynamic interactions between these domains, potentially influencing viral infectivity and immune evasion. In Omicron, the Ser19-Gly476 hydrogen bond, and less frequently Ser19-Asn477, arise due to the Ser477Asn mutation. Subsequent variants further refine these interactions, with Gly476 and Asn477 engaging in nonbonded interactions with Thr20 and Gln24, respectively. These interactions, absent in WT SARS-CoV-2, may contribute to the observed shifts in binding affinity and the emergence of new escape mechanisms.

Certain conserved residue interactions within the RBD-ACE2 interface, such as those involving Leu456 (with ACE2 Thr27 and Asp30) and Tyr489 (with Thr27, Gln24, Tyr83, and Lys31), are likely critical for maintaining ACE2 binding despite potential future mutations. Currently, the combination of the L455F and F456L mutations is observed to exert a compensatory effect at the ACE2 binding site, resulting in significantly increased affinity. This enhanced interaction contributes to greater resistance to neutralizing antibodies and increased binding affinity to the ACE2 receptor.^27^ Post-Omicron, a notable shift from stable hydrogen bonds to weaker nonbonded interactions is observed involving residues Gly476, Ala475, Phe486, and Asn487. This shift may be a consequence of Omicron-specific mutations, including those affecting Asn/Tyr501 and Tyr/His505, which also disrupt stable hydrogen bonds. Further details on these specific interactions are provided in the Supporting Information (Figures S4 and S10–S12).

Regions involving residues Tyr449 and Tyr453 exhibit dynamic interactions with Asp38 and Gln42, suggesting a potential for future salt bridge formation, similar to the observed Thr478Lys or Tyr505His mutations. Similar dynamic behavior is observed in residues Thr500 and Gly502. Conversely, conserved ACE2 interactions, including those involving Glu23, Gln24, Thr27, Lys31, His34, Glu35, Asp38, Tyr41, Met82, Tyr83, Asn330, Lys353, Gly354, and Asp355, represent prime targets for enhancing binding affinity through hydrogen bonds. These critical and conserved interactions provide crucial insights into the binding dynamics and potential future evolutionary trajectories of the virus. Furthermore, these conserved residues, essential for binding, represent promising targets for the development of neutralizing antibodies and small molecule inhibitors.

### Dissecting the RBD-ACE2 Interface: Implications for Viral Evolution and Therapeutic Targeting

To identify critical residues mediating the RBD-ACE2 interaction, we carried out Interaction Fingerprint Analysis (IFP) during 200 ns of accelerated molecular dynamics (aMD) simulations. We considered only interactions observed in more than 50% of the simulation time. IFP analysis is shown for SARS-CoV-2 (Figure 5a) and the EG.5 variant (Figure 5b). Results for other variants are available as Supporting Information. Table S3 shows the IFP interactions along with their occupancy values. Figures S4 and S10–S12 provides a three-dimensional visualization of key residue interactions identified by IFP within the RBD-ACE2 interface of SARS-CoV-2.

**Figure 5:**
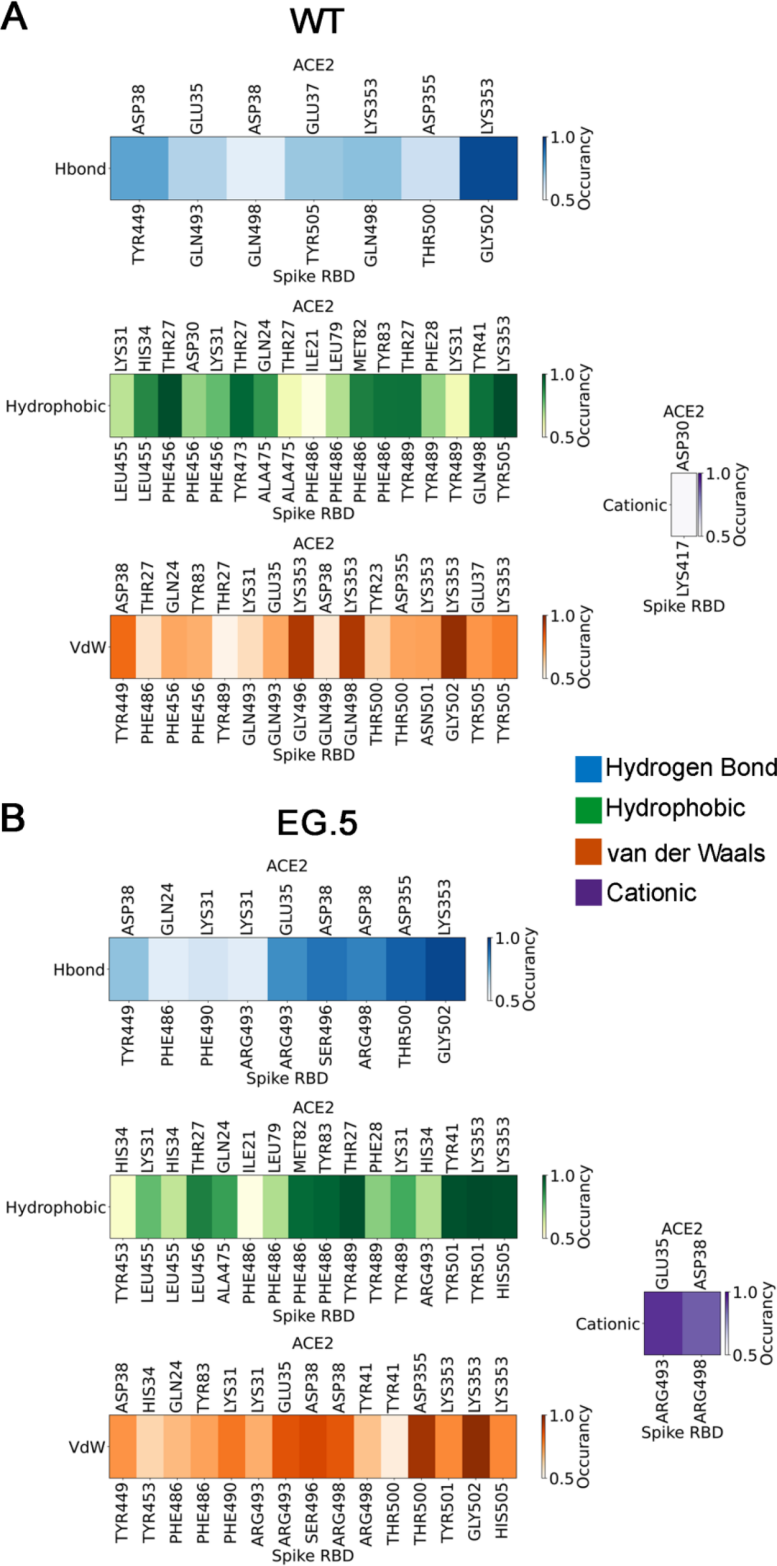
Fingerprint of Protein–protein Interaction between the Spike Binding Region and ACE2 for the systems (a) WT SARS-CoV-2 and (b) EG.5. The variations in color tones reflect the occurrence of interactions over a period of 200 ns for each studied system. The types of interaction are Hydrogen Bonds (H-bond) in blue; Hydrophobic Interactions in green; Cationic Interactions in purple; and van der Waals interactions in orange.

The IFP analysis reveals that the F456L mutation in EG.5 significantly reduces the hydrophobic interactions of the RBD with the ACE2 receptor. In the EG.5 variant, Leu456 engages in hydrophobic contact only with residue Thr27 (Occurancy 0.88), whereas in the WT, Phe456 forms multiple interactions with Thr27 (Occurancy 0.98), Asp30 (Occurancy 0.71), and Lys31 (Occurancy 0.72), in addition to van der Waals contacts with Gln24 (Occurancy 0.70) and Tyr83 (Occurancy 0.68). This reduction in contact frequency indicates that the F456L mutation does not directly strengthen ACE2 binding, supporting the hypothesis that its functional role is primarily related to immune evasion. This interpretation is consistent with previous experimental data showing that F456L occurs in critical epitope regions recognized by neutralizing antibodies, interfering with immune response without significantly affecting receptor affinity.^22,85^ Point substitutions such as L455F or F456L have been shown to substantially reduce ACE2 affinity in the BA.2 background. Both residues, 455 and 456, are part of a central epitope targeted by public Class 1 antibodies.^86^

In comparative analysis, it is observed that in XBB.1.9.2, Phe456 retains hydrophobic interactions with Thr27 (Occurancy 0.96), Asp30 (Occurancy 0.71), and Lys31 (Occurancy 0.80), similar to WT, reinforcing the observation that substitution to Leu in EG.5 substantially diminishes the involvement of this position in ACE2 binding. Altogether, these data support the model in which F456L is an adaptive mutation driven by immune escape, with minimal impact on receptor-binding affinity (Figure S4).

Our analysis identified ACE2 residues Gln24, Thr27, Lys31, His34, Glu35, Asp38, Tyr41, Leu79, and Lys355 as forming stable interactions across all variants throughout the simulations. Notably, the XBB.1.9.2 variant exhibited a weakened interaction interface, with absent interactions at positions Lys21, Phe28, Met82, Tyr83, and Asp355. Also, variant-specific interactions were observed: Omicron showed exclusive interactions at residue Lys371, while XBB.1.9.2 exhibited exclusive interactions at residues Glu23 and 354 (Figure S4).

Conserved interaction positions on the RBD surface include 455, 456, 475, 486, 489, 493, 498, 501, 502, and 505. Notably, XBB.1.9.2 exhibited absent interactions at residues 496 and 500, further indicating a weakened interaction interface in this variant. Omicron exhibits exclusive interactions at residues Arg490, indicating unique binding features compared to other variants, whereas XBB.1.9.2 showed exclusive interactions at residues Arg454, Arg493, and Gly504, suggesting potential differences in binding affinity. Figure S12 provides a visual representation of the key residues identified by IFP analysis.

These regions are of particular significance for viral evolution, as numerous variants exhibit alterations within these sites.^19,25,87–89^ The Omicron variant harbors mutations such as G493R, G498R, Y505H, and N501Y, which have been implicated in immune escape and enhanced transmissibility of the virus.^90–92^ IFP analysis revealed key differences between SARS-CoV-2 and Omicron, including an increase in the frequency of hydrogen bonding interactions and a modest decrease in hydrophobic and van der Waals interactions. The identification of these residues is key for understanding the RBD-ACE2 interaction and plays a pivotal role in the rational design of peptide inhibitors or bioactive compounds as potential therapeutic strategies against SARS-CoV-2 and its variants.

IFP analysis also revealed distinct behavior for the XBB.1.9.2 variant, characterized by less frequent and weaker (less persistent) interactions compared to SARS-CoV-2, Omicron, and EG.5 (Figure 5 and Figure S4). In particular, XBB.1.9.2 lacked interactions at residues Leu79 and Gly354 on ACE2, potentially impacting binding dynamics. This suggests that XBB.1.9.2 may adopt intermediate conformations that alter the binding mode and weaken interactions with the ACE2 receptor. Key residues involved in these interactions are highlighted in the structural representation of the XBB.1.9.2 RBD-ACE2 complex in Figure S12. Recent studies have demonstrated that modifications observed in EG.5 and its XBB sublineages not only enhance immune evasion but also significantly alter interactions with the human receptor.^33,85,93,94^

In contrast, analysis of the EG.5 variant, which exhibits increased infectivity and evades vaccine-induced antibodies, reveals more robust binding to the ACE2 receptor with significantly stronger interactions (Figure 5b) compared to the WT and its descendant lineages. While EG.5 does not exhibit any particularly unique interactions, it interestingly combines interactions observed exclusively in WT (residue Tyr449), Omicron (residue Arg490), and XBB.1.9.2 (residue Tyr453).

The enhanced binding strength of the EG.5 variant is further evidenced by the presence of more persistent hydrogen bonds (with frequency values above 0.8) between RBD residues (Arg493, Ser496, Arg498, Thr500, and Gly502) and ACE2 residues (Glu35, Asp38, Asp355, and Lys353), suggesting increased stability at the protein–protein interface, as depicted in Figure 6. Moreover, hydrophobic contacts and van der Waals interactions were observed more frequently throughout the simulations, highlighting the importance of key RBD residues, including Leu456, Ala475, Phe486, Tyr489, Tyr501, and His505. These residues are prone to mutations and play a crucial role in viral evolution.^90,92^ These robust interactions may contribute to enhance viral binding, potentially facilitating the infection process and subsequent viral replication within the host cell.

**Figure 6:**
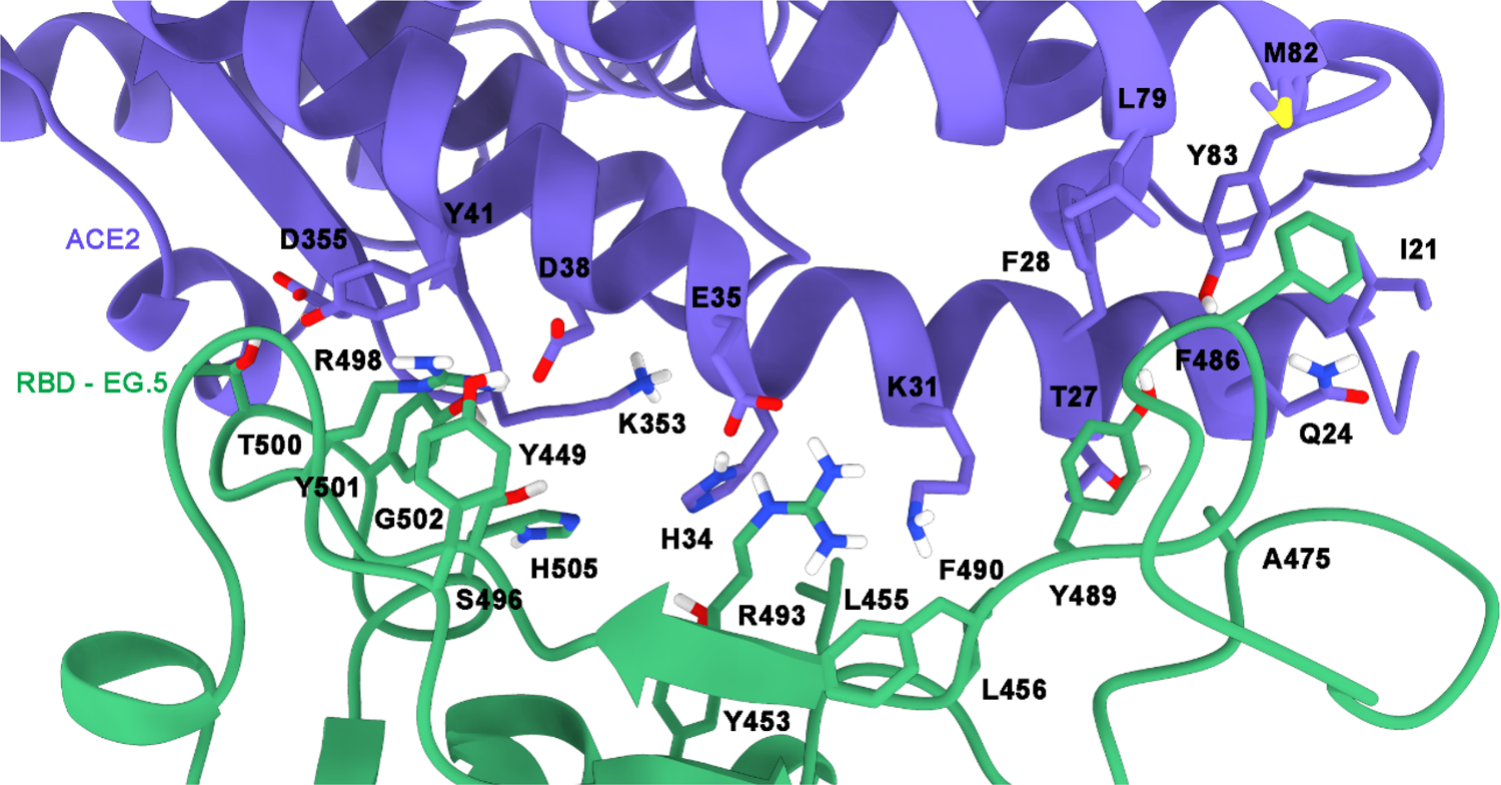
Tridimensional structure of ACE2 (in purple) and EG.5 RBD (in green) depicting the location of key residues identified by IFP during the 200 ns simulation.

### Energy Characterization of Spike-ACE2 Complexes

To assess the binding affinity of the EG.5 variant to the ACE2 receptor, we calculated binding free energies using the MM/GBSA method (Table 1).

**Table 1:** Binding Free Energy Results for SARS-CoV-2 Systems Calculated Using the MM/GBSA^a^

parameter	WT	Omicron	XBB.1.9.2	EG.5
Δ*E*_elec_	–610.70 ± 0.94	–1346.66 ± 1.15	–1379.37 ± 1.30	–1366.61 ± 1.07
Δ*E*_vdW_	–99.79 ± 0.17	–98.29 ± 0.20	–102.95 ± 0.21	–95.90 ± 0.17
Δ*G*_GB_	665.55 ± 0.89	1390.72 ± 1.09	1442.23 ± 1.27	1403.48 ± 1.02
Δ*E*_nonpolar_	–13.54 ± 0.02	–13.91 ± 0.02	–14.70 ± 0.03	–13.35 ± 0.02
Δ*G*_solv_	652.01 ± 0.88	1376.81 ± 1.08	1427.53 ± 1.26	1390.13 ± 1.01
Δ*G*_gas_	–710.49 ± 0.07	–1444.96 ± 0.13	–1482.32 ± 0.12	–1462.51 ± 0.09
Δ*G*_bind_	–58.49 ± 0.04	–68.15 ± 0.02	–54.79 ± 0.02	–72.38 ± 0.03

a All energy values are reported in kcal mol^–1^.

Understanding the thermodynamic aspects of binding affinity in protein–inhibitor systems is key for advancing the knowledge about molecular recognition. Thus, having an accurate and efficient method to calculate binding free energy (ΔG_bind_) is vital in computer-aided drug design.^95^ Various techniques for calculating ΔG_bind_ are available, ranging from rigorous approaches like thermodynamic integration (TI)^96^ and free energy perturbation (FEP)^97^ to simpler empirical scoring functions used in molecular docking.^98^ The stringent methods are computationally expensive because they require extensive sampling of multiple intermediate, nonphysical states, making them less suitable for high-throughput applications.^99^ The MM/GBSA approach used here is an alternative that balances effectiveness and efficiency, considering the configurational space for the ligand, protein–ligand interactions, and solvent in both bound and unbound states.^100–102^ The MM/GBSA approach has been successfully used in previous studies of SARS-CoV-2 systems.^103–109^

The thermodynamic analysis of gas-phase energies reveals significant distinctions between the WT strain and its subsequent variants, including Omicron, XBB.1.9.2, and EG.5. The WT strain has a total gas-phase energy of – 710.49 kcal/mol, while the Omicron variant demonstrates a remarkably lower total gas-phase energy of – 1444.96 kcal/mol. This trend continues with XBB.1.9.2 at – 1482.32 kcal/mol and EG.5 at – 1462.51 kcal/mol, indicating an approximate 2-fold enhancement in gas-phase stabilization in these variants compared to the WT.

Electrostatic interactions between the viral spike protein and the ACE2 receptor are the predominant driver for this increase in stabilization. The electrostatic energy component, ΔE_ele_, exhibits a substantial increase, shifting from – 610.70 kcal/mol in the WT strain to – 1346.66 kcal/mol in Omicron, – 1379.37 kcal/mol in XBB.1.9.2, and – 1366.61 kcal/mol in EG.5. This stark increase highlights the role of favorable polar interactions in enhancing the binding affinity of these variants. Conversely, the van der Waals energy component (ΔE_vdW_) remains relatively the same across all variants, ranging from approximately – 95 to – 103 kcal/mol. This indicates that variations in binding affinity across the mutants are predominantly electrostatically mediated rather than through changes in van der Waals forces.

Regarding solvation contributions, the solvation penalty includes contributions from the generalized Born solvation energy (ΔG_GB_) and the nonpolar solvation energy (ΔE_nonpolar_). The Born solvation energy scales with the enhanced gas-phase interactions. The WT strain presents a solvation energy of +652.01 kcal/mol, which sharply increases to +1376.81 kcal/mol for Omicron, + 1427.53 kcal/mol for XBB.1.9.2, and +1390.13 kcal/mol for EG.5. Conversely, the nonpolar solvation term remains nearly constant and relatively small (−13 to – 14 kcal/mol). These results corroborate the idea that the main compensatory factor counterbalancing the stronger gas-phase binding is the polar desolvation cost incurred when the protein interacts with the solvent and the ACE2 receptor.

Among the systems studied here, the EG.5 variant exhibits the most favorable total binding free energy value, suggesting it has the highest predicted binding affinity to the ACE2 receptor. Omicron follows closely, as it also displays a significant binding strength. Even though XBB.1.9.2 achieves the strongest gas-phase stabilization, its overall ΔG_bind_ turns out slightly higher (less favorable) than that of Omicron and EG.5. This is likely due to an overcompensation effect resulting from solvation penalties arising from the variant’s enhanced interactions with the polar environment.

### Energy Decomposition Analysis

While there may not be a stringent method to break down free energies into their contributions from protein–ligand systems through MD simulations,^110,111^ we can effectively compute the electrostatic and van der Waals interaction energies of the SARS-CoV-2 Spike complexes in order to assess key binding residues and how they relate to available experimental findings.

The electrostatic energy landscape at the spike-ACE2 interface shows significant modifications between the original SARS-CoV-2 virus and its Omicron, XBB.1.9.2, and EG.5 variants, with critical variations localized at specific residues within the RBD (Figure 7). Asp405 plays a pivotal role: in the original SARS-CoV-2, Asp405 contributes a markedly unfavorable electrostatic energy of +111.7 kcal/mol. In contrast, the XBB.1.9.2 and EG.5 variants show a reversal to – 2.78 kcal/mol and – 1.92 kcal/mol, respectively. Substituting Asp with Asn effectively neutralizes a significant destabilizing feature, transforming the local interaction milieu to one favoring ACE2 binding. Another critical site, Arg493, exhibits a substantial enhancement of electrostatic interactions across the variants. The energy profile shifts dramatically from – 11.4 kcal/mol in SARS-CoV-2 to – 137.7 kcal/mol in Omicron, – 149.8 kcal/mol in XBB.1.9.2, and – 152.1 kcal/mol in EG.5. This trend indicates that Arg493 serves as an electrostatic anchor in the mutant RBDs, thereby enhancing the stability of the spike-ACE2 complex. Similarly, Arg498 exhibits a significant transition from a modest – 4.2 kcal/mol in SARS-Cov-2 to – 117.4 kcal/mol in Omicron and further to – 147.7 kcal/mol and – 153.3 kcal/mol in XBB.1.9.2 and EG.5, respectively, which reinforces its role as a crucial contributor to electrostatics at the evolved interface. Conversely, Lys417 in the original strain presents a strong, favorable interaction of – 123.0 kcal/mol. However, upon mutation to Asn417 in Omicron and its progeny, this contribution diminishes markedly to – 25.5 kcal/mol in Omicron and further declines to near-neutral values of – 2.22 kcal/mol in XBB.1.9.2 and – 0.57 kcal/mol in EG.5. This attenuation likely reflects an evolutionary adaptation aimed at evading neutralizing antibodies that target the Lys417 site, albeit at the expense of local electrostatic stability. For Glu484, the original virus shows a severely unfavorable positive energy of +91.6 kcal/mol, which is significantly mitigated to +15.8 kcal/mol in Omicron. It is effectively neutralized to – 0.62 kcal/mol and – 0.97 kcal/mol in XBB.1.9.2 and EG.5, respectively. This shift suggests a marked reduction in unfavorable electrostatic repulsion at this position, potentially enhancing receptor binding and facilitating immune evasion.

**Figure 7:**
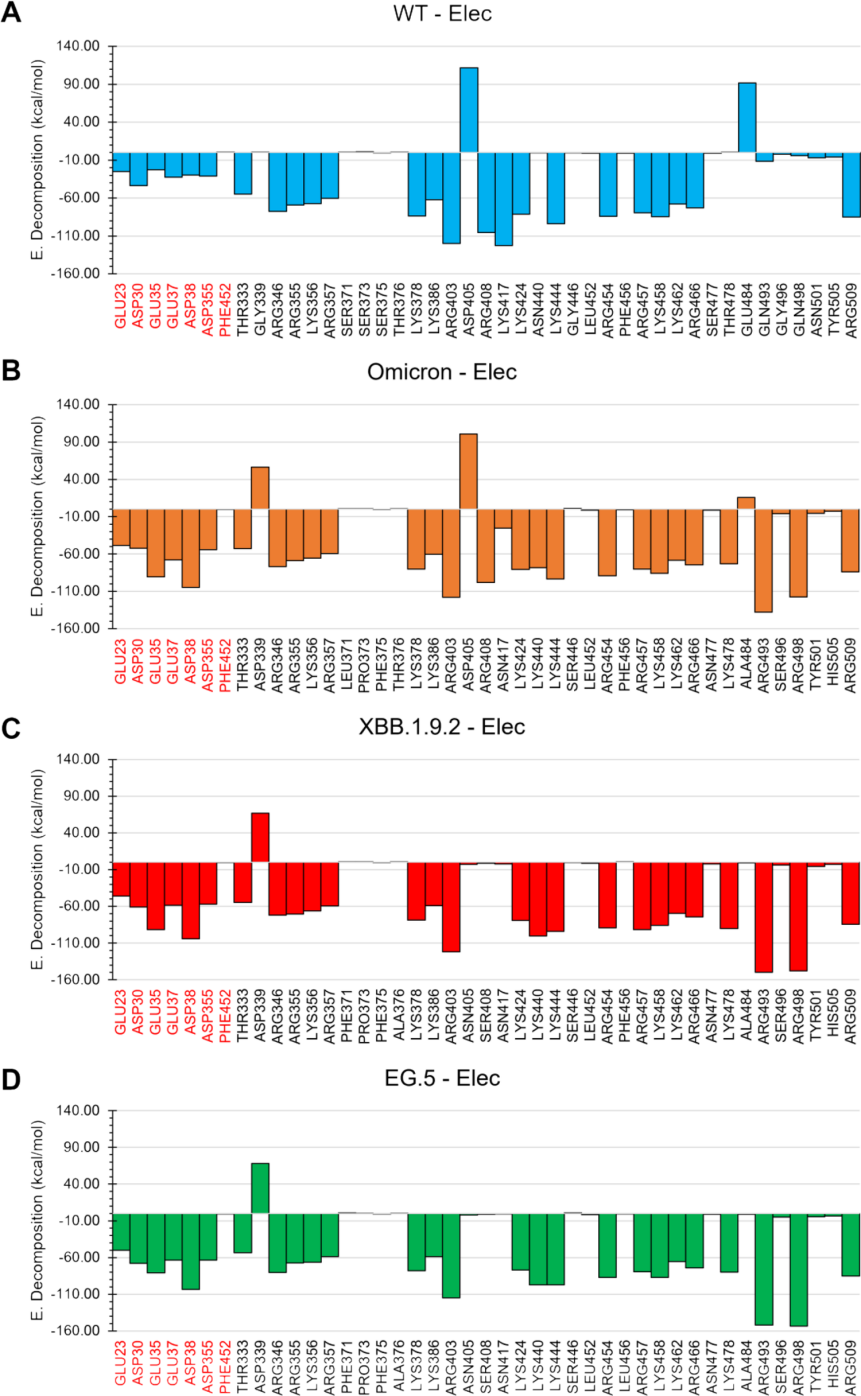
Electrostatic energy decomposition (in kcal/mol) for individual residues at the RBD–ACE2 interface in the (A) wild-type (WT), (B) Omicron, (C) XBB.1.9.2, and (D) EG.5 variants. Residues shown were selected based on significant energetic contributions across at least one variant. Positive values indicate unfavorable electrostatic interactions, while negative values reflect favorable contributions to complex stabilization. Residue names in red correspond to ACE2, while those in black belong to the Spike RBD.

Interestingly, while Asn501 maintains nearly the same energy contributions (−6.8 kcal/mol in SARS-CoV-2, – 5.7 kcal/mol in Omicron, – 5.5 kcal/mol in XBB.1.9.2, and – 4.2 kcal/mol in EG.5), the surrounding electrostatic context is significantly remodeled among the mutant lineages, thereby amplifying cooperative stabilization across the RBD-ACE2 interface. The significance of residue-level analysis lies in its capacity to elucidate local energetic contributions that collectively influence macroscopic binding characteristics. Without scrutinizing interactions at the individual residue level, critical hotspots or compensatory alterations may remain obscured, constraining mechanistic insights. Besides, these results indicate that the Omicron variant carries mutations such as G493R, G498R, Y505H, and N501Y, which are associated with immune escape and increased virus transmissibility.^89,90,92^

Despite the F456L mutation in EG.5, identified as critical for immune evasion, electrostatic analysis reveals no significant changes at this position when compared to the original, Omicron, and XBB.1.9.2 variants. This suggests that F456L does not enhance ACE2 binding via electrostatic interactions, but instead promotes neutralizing antibodies escape through conformational or structural alterations, consistent with previous reports on EG.5-mediated immune evasion.^27,35,85,93^

The molecular interplay between the RBD and ACE2 is central to delineating the mechanistic underpinnings of viral attachment and entry, providing critical structural and energetic determinants for the rational development of antiviral agents and vaccine formulations. Our thermodynamic analyses indicate that viral evolution does not merely enhance binding affinity through the amplification of favorable intermolecular interactions but rather orchestrates a sophisticated balance between gas-phase stabilization and desolvation penalties. This optimized energetic profile is likely sculpted by evolutionary pressures to fine-tune the spike–ACE2 interface, thereby maximizing viral infectivity while preserving conformational plasticity and facilitating immune escape. Deciphering this complex energetic landscape is pivotal for informing the design of high-affinity therapeutic inhibitors and broadly neutralizing antibodies capable of targeting evolved viral variants — a pursuit of paramount importance in the ongoing efforts to combat the COVID-19 pandemic and to develop adaptable, next-generation immunotherapeutic strategies. The detailed values for each individual residue are presented in Table S4.

To assess the binding affinity of the EG.5 variant to the ACE2 receptor, we calculated binding free energies using the MM/GBSA method (Table 1). Our results demonstrate that while WT SARS-CoV-2 exhibits a lower binding energy compared to Omicron, XBB.1.9.2 did not show a statistically significant increase in binding energy compared to WT SARS-CoV-2. In contrast, the EG.5 variant exhibited a significantly lower binding free energy, indicating a higher affinity for the ACE2 receptor. These findings align with experimental evidence that classifies EG.5, a descendant of XBB.1.9.2, as a variant of concern due to its enhanced binding affinity relative to SARS-CoV-2, Omicron, and XBB.1.9.2.^24–26,33^

Our findings strongly support the observed dominance of the EG.5 variant, which exhibits a significantly increased affinity for the ACE2 receptor. The F456L mutation, a key distinguishing feature of EG.5, appears to play a crucial role in this enhanced affinity. This mutation likely facilitates improved interactions with essential residues, leading to increased binding stability as evidenced by a more favorable ΔG. These results support the hypothesis that XBB.1.9.2 represents a transitional subvariant, while the prominent EG.5 variant demonstrates a significant increase in binding strength and affinity for the ACE2 receptor.

### Structural Implications for the Design of Inhibitors and Antibodies Targeting the SARS-COv-2 RBD

The development of novel antibodies capable of efficiently recognizing and interacting with the structural changes introduced by FLip lineages, particularly at critical epitope positions such as residues 455 and 456 in the RBD, represents a promising strategy for viral neutralization. Identifying key residues for inhibitor design enables direct interference with the RBD–ACE2 interaction, offering a therapeutic and preventive approach to block the initial stage of SARS-CoV-2 infection.^112–114^

Among these strategies, the development of ACE2-mimetic peptides stands out, as they can competitively bind to the spike RBD. Based on key interaction hotspots identified through fingerprint analysis, RBD residues such as Gln493Arg, Gln498Arg, Thr500, Gly496Ser, and Tyr499 emerge as critical hydrogen bonding targets. For the rational design of peptide inhibitors, the inclusion of acidic and uncharged polar residues is proposed to establish specific interactions with these sites. In addition, hydrophobic residues including Leu455, Phe456Leu, Tyr473, Ala475, Phe486, Tyr489, Asn501Tyr, and Tyr501His contribute significantly to stabilizing the RBD–ACE2 interface via apolar contacts. Accordingly, an ideal peptide inhibitor should incorporate nonpolar and aromatic amino acids to effectively engage these hydrophobic hotspots on the RBD.

Our findings also highlight the essential role of electrostatic interactions in RBD–ACE2 binding affinity. Positively charged residues in the RBD, such as Arg403, Lys440, Lys444, Arg493, and Arg498, exhibit strong attractive contributions, suggesting electrostatic complementarity with negatively charged ACE2 residues such as Glu35, Asp38, and Glu37. Thus, rational inhibitor design should also include acidic residues to enhance interface electrostatic matching. Furthermore, RBD epitopes may serve as targets for vaccine development aimed at eliciting more effective immune responses. Specifically, residues associated with the FLip lineage, such as the F456L mutation, did not show increased contributions to ACE2 binding in our models, reinforcing their role in immune evasion. These insights support the design of neutralizing antibodies targeting this region, as reported in recent studies.^115–118^

## Conclusions

This study provides molecular insights into the dynamic interplay between the SARS-CoV-2 spike protein Receptor-Binding Domain (RBD) and the human ACE2 receptor across key variants, including WT, Omicron, XBB.1.9.2, and EG.5. The FEL analysis highlights an interplay of conformational states across SARS-CoV-2 variants, including WT, Omicron, XBB.1.9.2, and EG.5. Both minimum (M1, M2) and intermediate (IN) states are observed in these variants, suggesting a complex landscape of conformational transitions. The stability of these conformational states is significantly influenced by the formation of salt bridges. While the WT exhibits a unique Asp30-Lys417 salt bridge in its M2 state, variants such as Omicron, XBB.1.9.2, and EG.5, despite displaying stable M1 and M2 states, necessitate transitions through intermediate states (IN1 and IN2) to shift between these minima. These intermediate transitions involve conformational adjustments facilitated by the formation of new salt bridges, such as Glu35-Arg493 and Asp38-Arg498, observed in these variants. The *in silico* observation of these intermediate states suggests that the virus is evolving to explore more complex dynamic interactions. This increased conformational flexibility and enhanced stability of ACE2 interactions may contribute to the observed resistance to neutralizing antibodies and the high infectivity of these variants.

Interaction Fingerprint Analysis (IFP) revealed that hydrophobic and van der Waals interactions occurred with greater frequency, underscoring the critical role of specific RBD residues such as Leu456, Ala475, Phe486, Tyr489, Tyr501, and His505. Our analyses identified conserved interactions on the ACE2 receptor involving positions Gln24, Thr27, Lys31, His34, Glu35, Asp38, Tyr41, Leu79, and Lys355 across all variants. Variant-specific interaction patterns were observed. Omicron exhibited unique interactions at residue 371, while XBB.1.9.2 displayed weakened interactions at residues Lys21, Phe28, Met82, Tyr83, and Asp355, suggesting distinct binding modes and potentially transitional characteristics for XBB.1.9.2. Analysis of the EG.5 variant revealed significantly enhanced binding to the ACE2 receptor compared to WT and its descendants. IFP analysis, coupled with strong hydrogen bond interactions (e.g., between RBD residues Arg493, Ser496, Arg498, Thr500, Gly502 and ACE2 residues Glu35, Asp38, Asp355, Lys353), indicated increased stability at the protein–protein interface.

While hydrophobic and vdW interactions dominate the interface in terms of frequency (*c.f*, Figure 5), electrostatic interactions are the primary energetic drivers of complex stabilization (*c.f,* Table 1, Figure 7). This finding demonstrates that the most frequent interactions are not necessarily the most energetically significant, making it crucial to consider both metrics in the analysis. The energetic decomposition analysis reveals that point mutations at key residues reconfigure the local electrostatic environment, contributing to the stabilization of the Spike–ACE2 complex. These findings demonstrate that the enhanced binding affinity in the Omicron, XBB.1.9.2, and EG.5 variants is primarily driven by intensified electrostatic interactions, with specific mutations such as G493R, G498R, and N501Y playing critical roles in stabilizing the Spike–ACE2 complex. Consistent with these findings, MM/GBSA calculations demonstrated a lower binding free energy for EG.5, suggesting higher receptor affinity. The F456L mutation in EG.5 likely plays a role in this enhanced binding by facilitating improved interactions with key residues. These results strongly support the hypothesis that EG.5, characterized by increased infectivity and immune evasion, has established a more stable interaction with the ACE2 receptor.

Our findings also highlight critical factors for the rational design of inhibitors, primarily based on key binding residues identified through IFP and per-residue decomposition analyses. Additionally, residues from the FLip lineage located within RBD epitopes showed no significant alterations in interaction forces or electrostatic contributions, suggesting their critical role in the recognition of neutralizing antibodies. This knowledge can guide the development of more effective vaccines targeting specific regions, selected based on their structural relevance, interaction profile, and energetic contribution. The identification of key binding hotspots, together with the characterization of stabilizing forces at the spike–ACE2 interface, provides valuable insights for the design of ACE2-mimetic peptides and therapeutic antibodies with improved specificity and efficacy against emerging variants. Altogether, these results support a data-driven, structure-based approach to address the ongoing evolution of SARS-CoV-2.

## Supplementary Material



## Data Availability

Representative PDB structures, data, and scripts are publicly available on GitHub (Costa, C. H. S. (2025). GitHub (https://github.com/ClauberHSCosta/SARS-CoV-2).

## Notes

Total potential and dihedral energies during MD simulations, variables and parameters used in the aMD simulations, average RMSD values and distances for all systems during aMD simulations, fingerprint of protein–protein interaction between the spike binding region and ACE2, representation of the free energy landscape with energy minima, and representative tridimensional structures of human ACE2 and SARS-CoV-2 RBD domain (wt and several mutants) (PDF)
