# Supplementary material for: Conformational Dynamics and Binding Interactions of SARS-CoV‑2 Spike Protein Variants: Omicron, XBB.1.9.2, and EG.5
Source: J Chem Inf Model. 2025 Jul 11;65(14):7651–67. doi: 10.1021/acs.jcim.5c00308 (PMC12308811; doi:10.1021/acs.jcim.5c00308)
Supplement: Supplementary file 1 [file ci5c00308_si_001.pdf]

Supporting Information

Conformational Dynamics and Binding Interactions of SARS-CoV-2 Spike Protein Variants: Omicron, XBB.1.9.2 and EG.5

Clauber Henrique Souza da Costa,[1] Camila Auad Beltrão de Freitas,[2] Alberto Monteiro dos Santos,[1] Carlos Gabriel da Silva de Souza,[2] José Rogério A. Silva,[3,4] Jerônimo Lameira,[2,3] Vicent Moliner, [5] Munir S. Skaf [1,*]

[1] Institute of Chemistry and Center for Computing in Engineering & Sciences, University of Campinas – UNICAMP. Campinas, SP 13084-862, Brazil.

[2] Laboratório de Planejamento e Desenvolvimento de Fármacos, Instituto de Ciências Exatas e Naturais, Universidade Federal do Pará, 66075-110, Belém, Pará, Brazil.

[3] Laboratory of Computer Modeling of Molecular Biosystems (CompMBio), Federal University of Pará, Belém 66075-110, Brazil.

[4] Catalysis and Peptide Research Unit, University of KwaZulu-Natal, Durban 4000, South Africa.

[5] Institute of Advanced Materials (INAM), Universitat Jaume I, Castellon, Spain.

*Corresponding author: E-mail: skaf@unicamp.br

Representative PDB structures, data, and scripts are publicly available on GitHub (Costa, C. H. S. (2025). GitHub.(https://github.com/ClauberHSCosta/SARS-CoV-2).

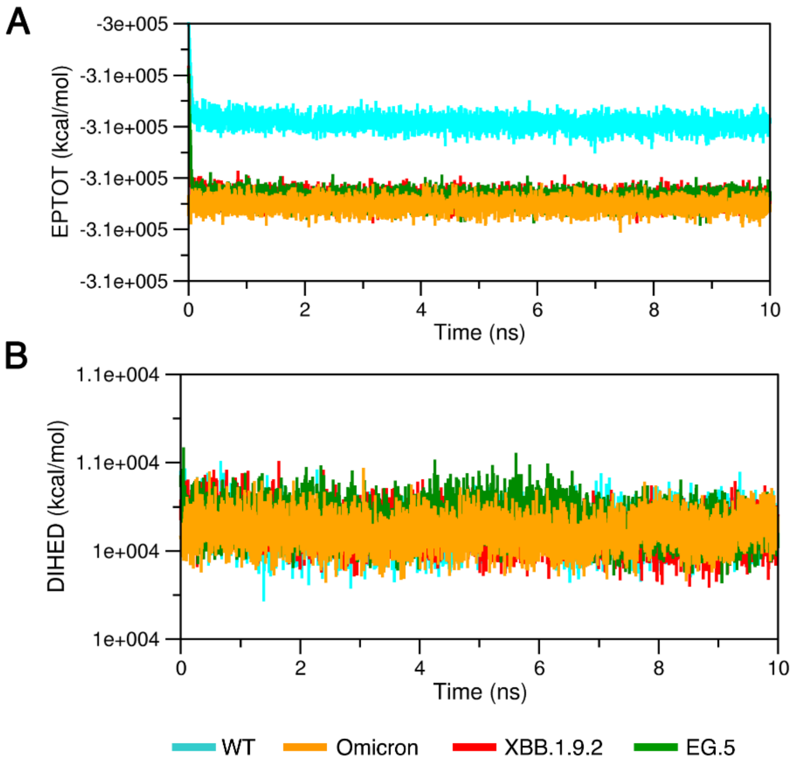

Figure S1: **A)** Total potential energy (EPTOT) and **B)** DIHED during 10ns of cMD for the complex between RBD of protein spike variants and ACE2.

Table S1: Variables and parameters used in the aMD simulation for all systems, obtained during cMD

System	Variables				Calculated Parameters			
	EPtot (kcal/mol)	DIHED (kcal/mol)	Total Atoms	Residues	Ethreshd	Alphad	Ethreshp	Alphap
**WT**	-307852.2225	10452.9602	102041	791	13616.9602	632.8	-291525.6625	16326,56
**Omicron**	-311012.6842	10478.9487	103159	791	13642.9487	632.8	-294507.2442	16505.44
**XBB.1.9.2**	-310764.1916	10449.9378	103163	791	13613.9378	632.8	-294258.1116	16506.08
**EG.5**	-310770.6513	10466.2743	103162	791	13630.2743	632.8	-294264.7313	16505.92

Table S2: Average RMSD values and distances for all systems and replicas computed during the 200ns of aMD. Standard deviation in brackets. All values are in Å.

Average RMSD				Distance between SpikeRBD and ACE2
**System**	**ACE2**	**$Spike_{RBD}$**	**Complex**	
**WT**	2.71(0.55)	3.17(0.43)	3.53(0.53)	49.02(0.86)
**Omicron**	3.18(0.74)	2.73(0.24)	4.42(0.72)	48.41(0.88)
**XBB.1.9.2**	2.17(0.32)	2.34(0.19)	3.35(0.53)	44.83(2.28)
**EG.5**	1.98(0.37)	1.94(0.19)	2.75(0.57)	49.73(0.86)
**Replica 2**				
**WT**	2.95(0.34)	2.60(0.26)	3.57(0.41)	47.20(0.93)
**Omicron**	2.40(0.38)	2.95(0.32)	3.75(0.57)	45.63(1.39)
**XBB.1.9.2**	3.52(0.58)	5.31(0.88)	5.15(0.69)	48.54(1.84)
**EG.5**	2.56(0.32)	2.92(0.52)	3.96(0.59)	47.43(1.40)
**Replica 3**				
**WT**	3.62(0.83)	3.06(0.70)	4.70(1.13)	46.52(1.32)
**Omicron**	2.78(0.29)	3.17(0.35)	3.69(0.37)	48.29(0.81)
**XBB.1.9.2**	4.12(0.80)	4.97(0.90)	7.32(1.78)	50.84(1.49)
**EG.5**	3.62(0.89)	2.53(0.25)	4.92(1.12)	48.40(1.0)

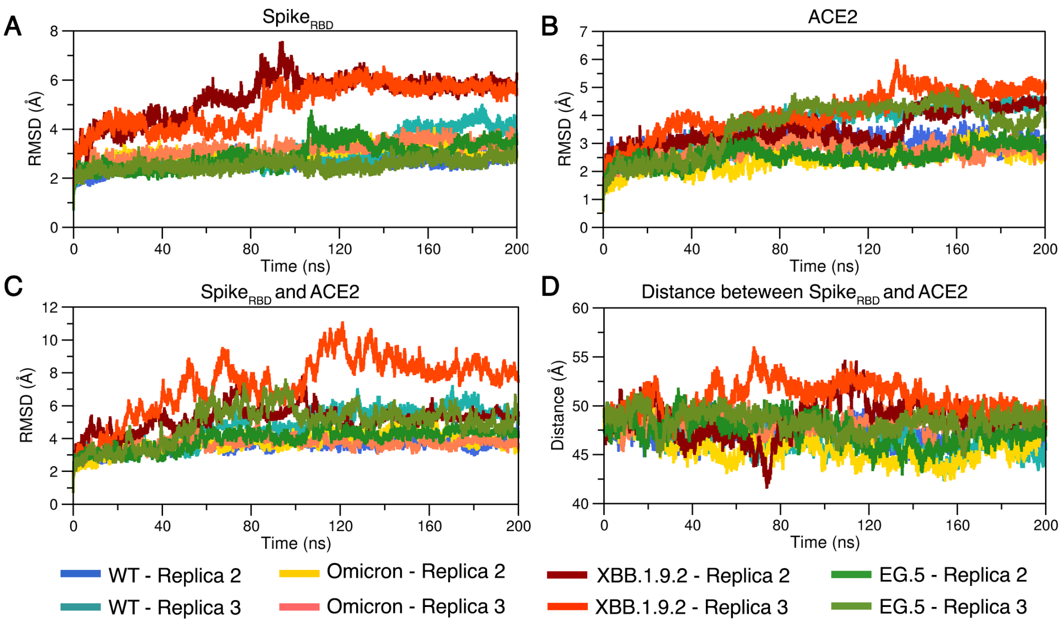

Figure S2: RMSD Analysis for WT, Omicron, XBB.1.9.2, and EG.5. a) RMSD for the RBD system. b) RMSD for the ACE2 receptor. c) RMSD for the complex formed by RBD + ACE2. d) Distance between RBD and ACE2 for replicas 1 and 2 from aMD simulations.

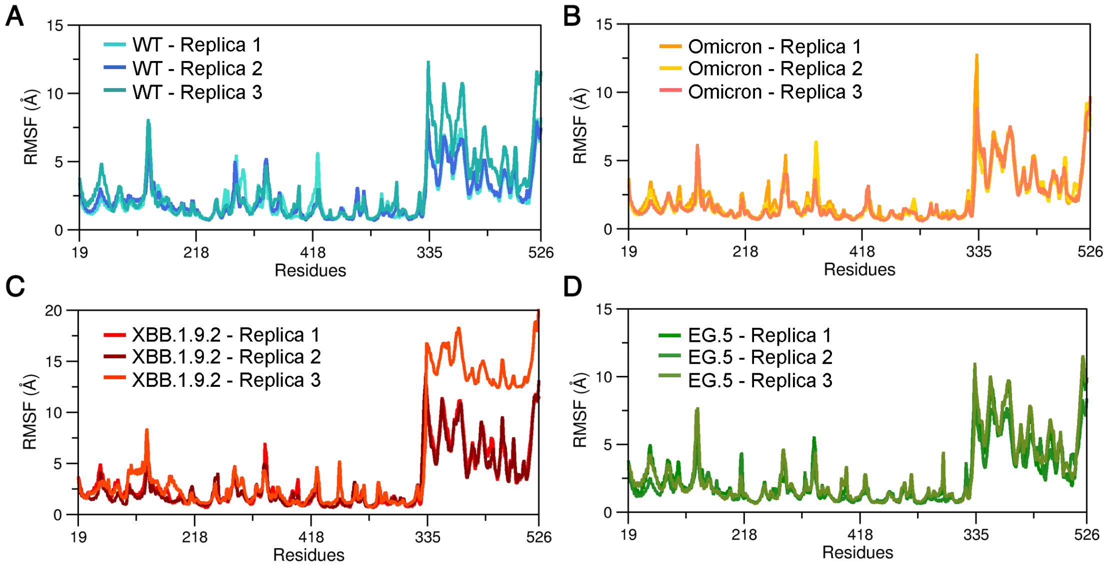

Figure S3: Root Mean Square Fluctuation (RMSF) of the RBD–ACE2 complex for three independent replicas of each variant: A) WT, B) Omicron, C) XBB.1.9.2, and D) EG.5. The RMSF values (Å) are plotted as a function of residue number.

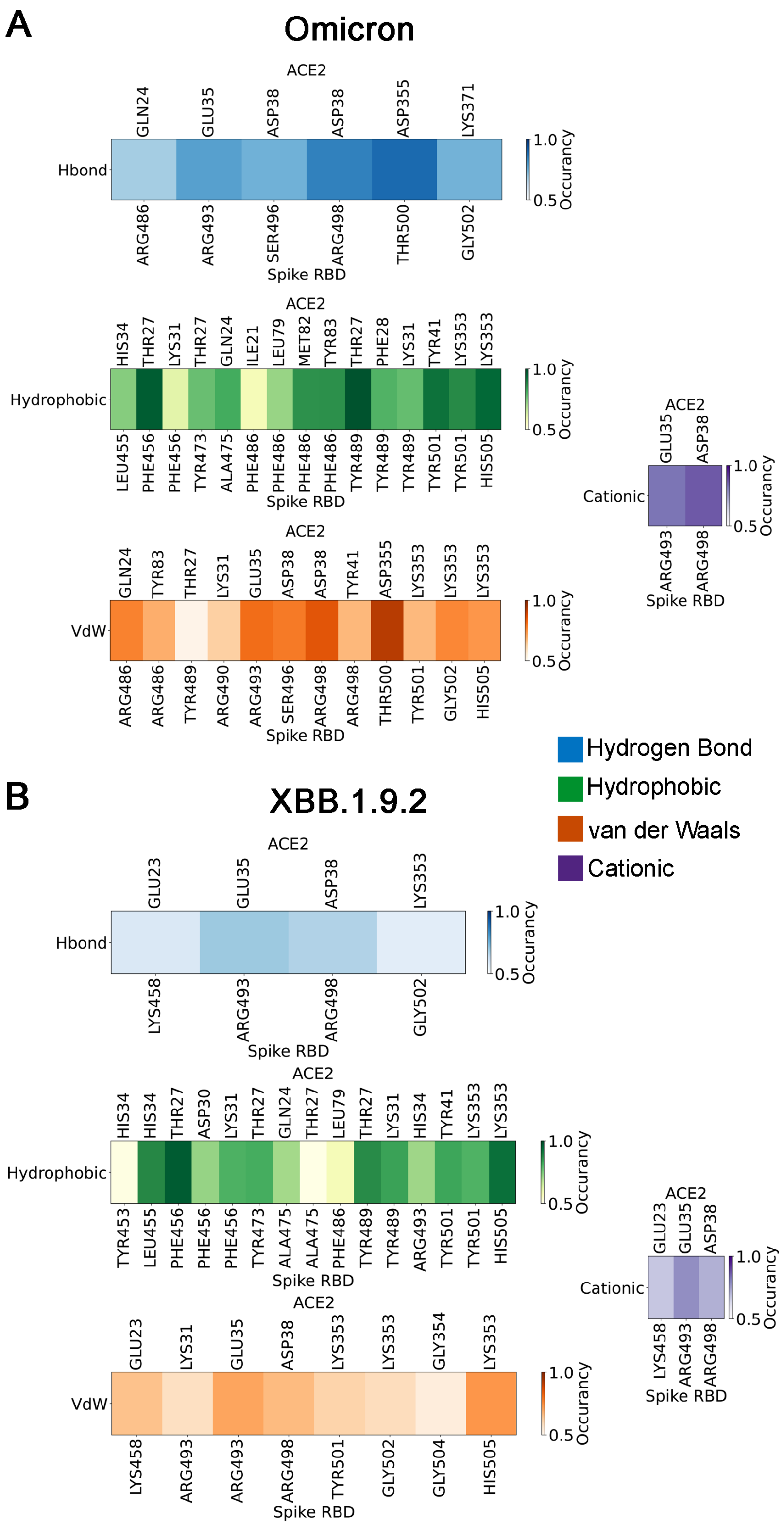

Figure S4: Fingerprint of Protein-Protein Interaction between the Spike Binding Region and ACE2 for the systems a) Omicron and b) XBB.1.9.2. The variations in color tones reflect the occurrence of interactions over a period of 200 ns for each system studied. The types of interaction are: Hydrogen Bonds (Hbond) in blue. Hydrophobic

Interactions in green. Cationic Interactions in purple. In orange, the van der Waals Forces.

A

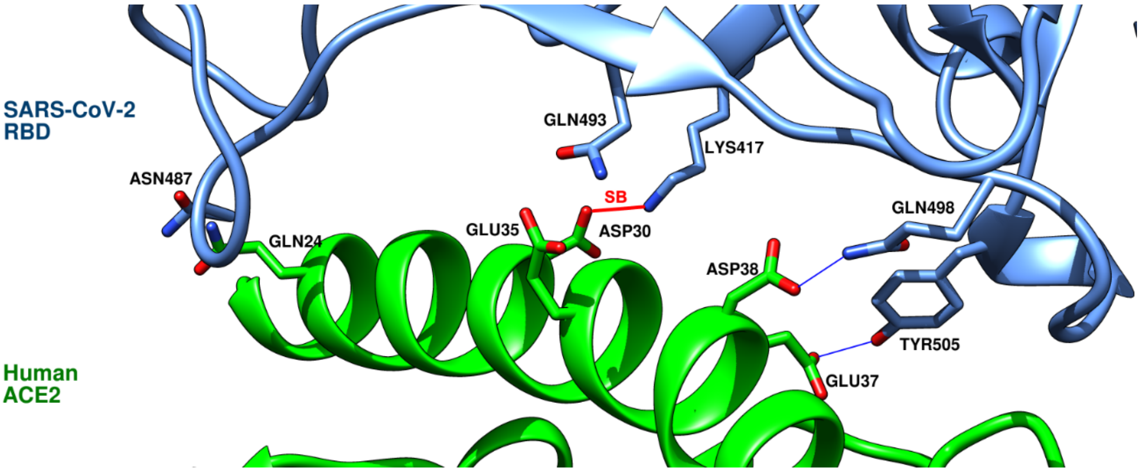

B

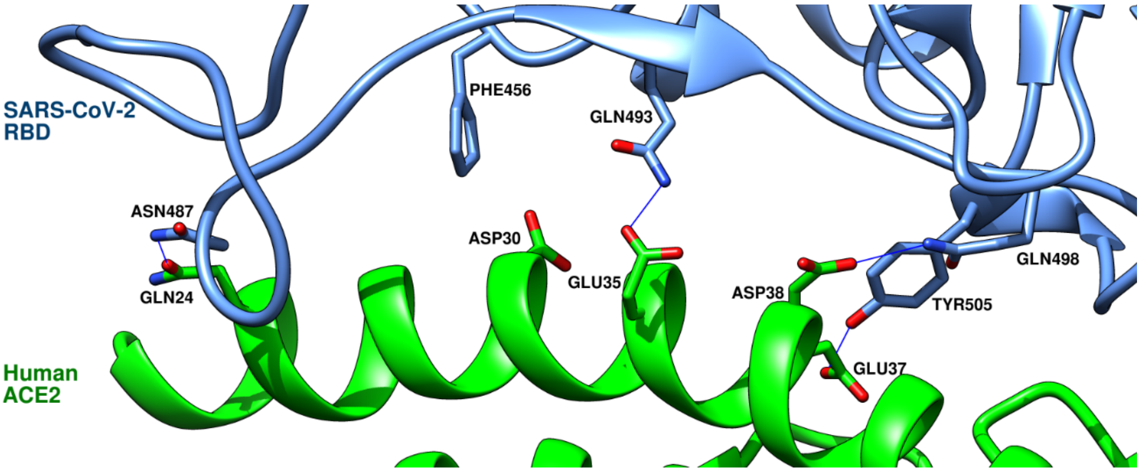

Figure S5: Representative Tridimensional structure of human ACE2 (in green) interface with a) minimum 1 (M1) and b) minimum 2 (M2) of SARS-CoV-2 RBD domain (in Cyan). Salt bridges (SB) are highlighted in red.

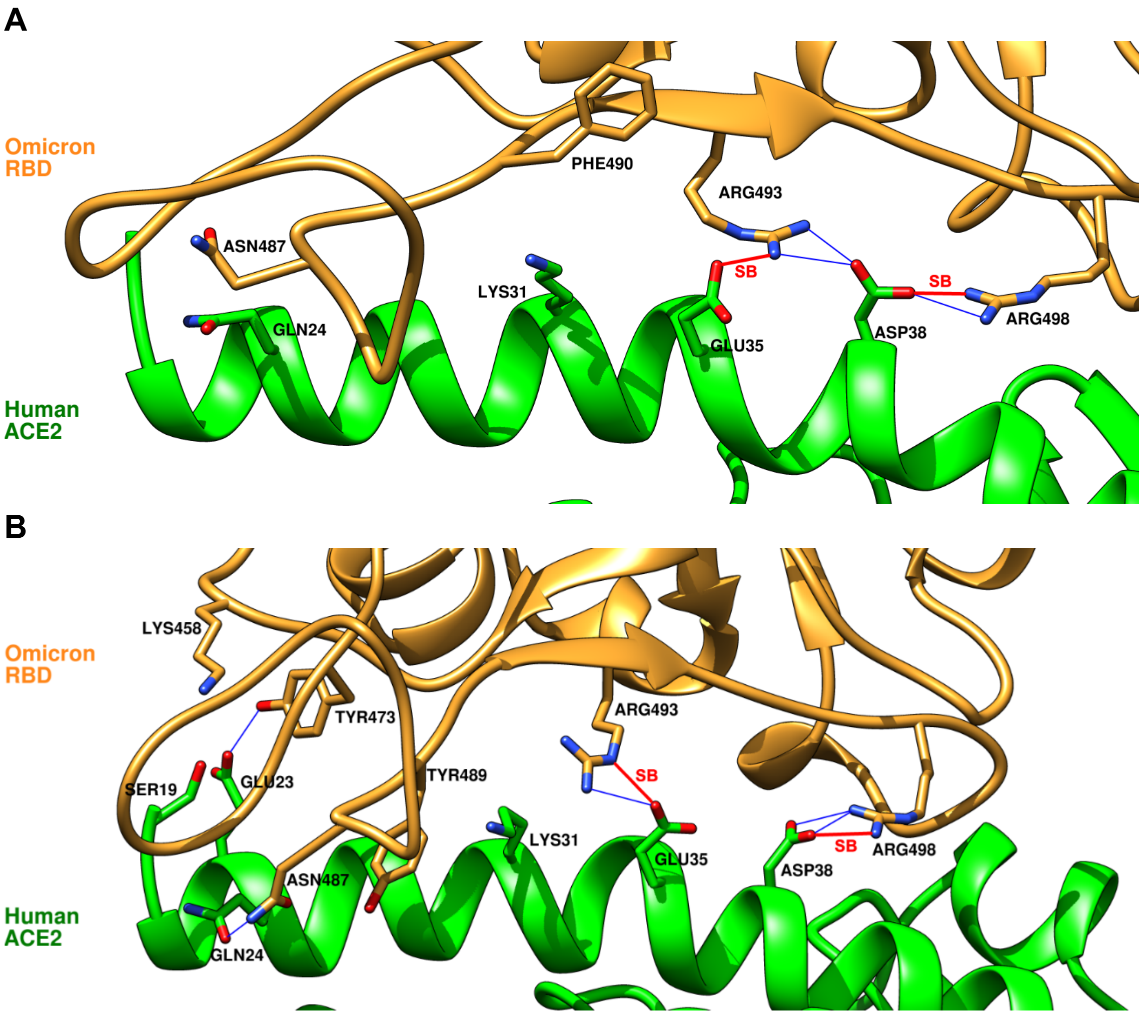

Figure S6: Representative Tridimensional structure of human ACE2 (in green) interface with a) minimum 1 (M1) and b) minimum 2 (M2) of Omicron RBD domain (in Orange). Salt bridges (SB) are highlighted in red.

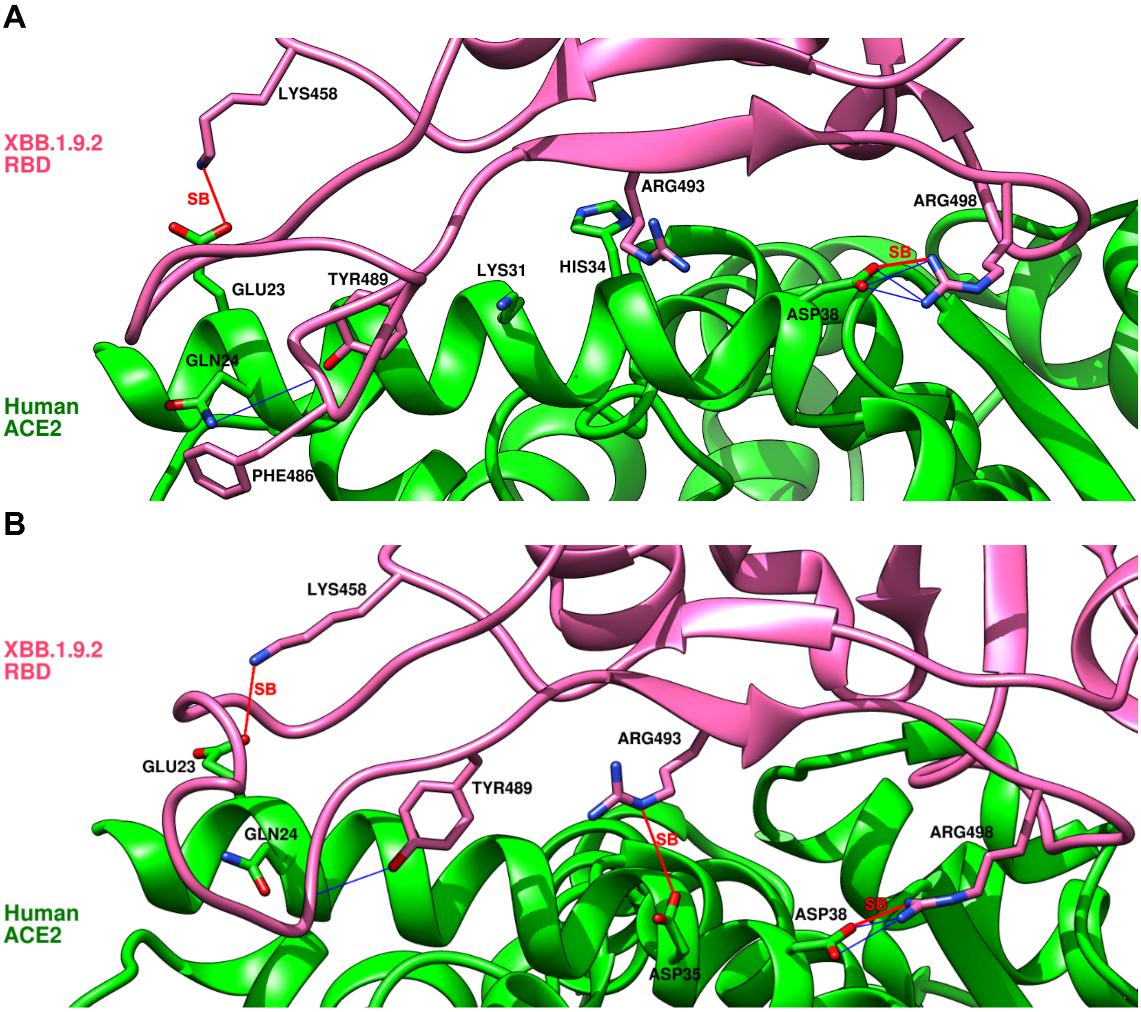

Figure S7: Representative Tridimensional structure of human ACE2 (in green) interface with a) minimum 1 (M1) and b) minimum 2 (M2) of XBB.1.9.2 RBD domain (in Pink). Salt bridges (SB) are highlighted in red.

A

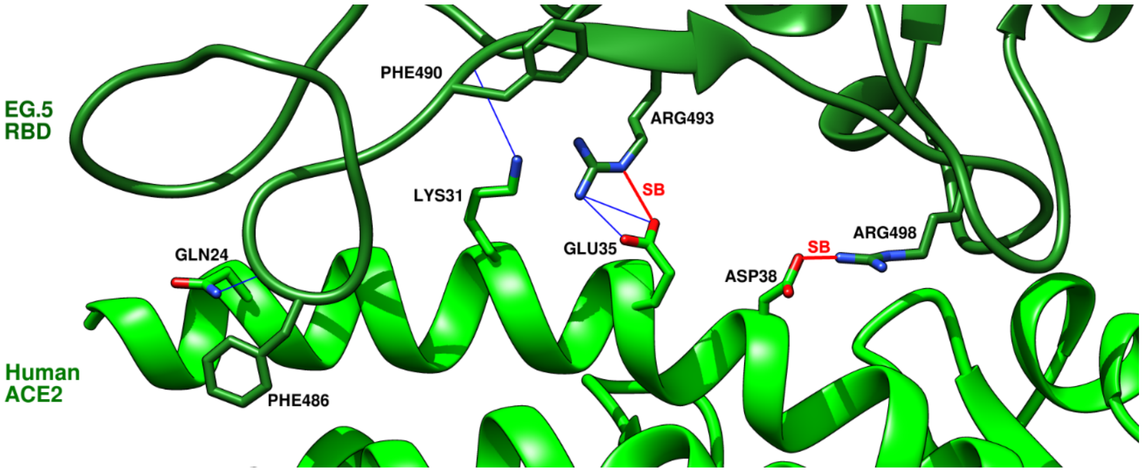

B

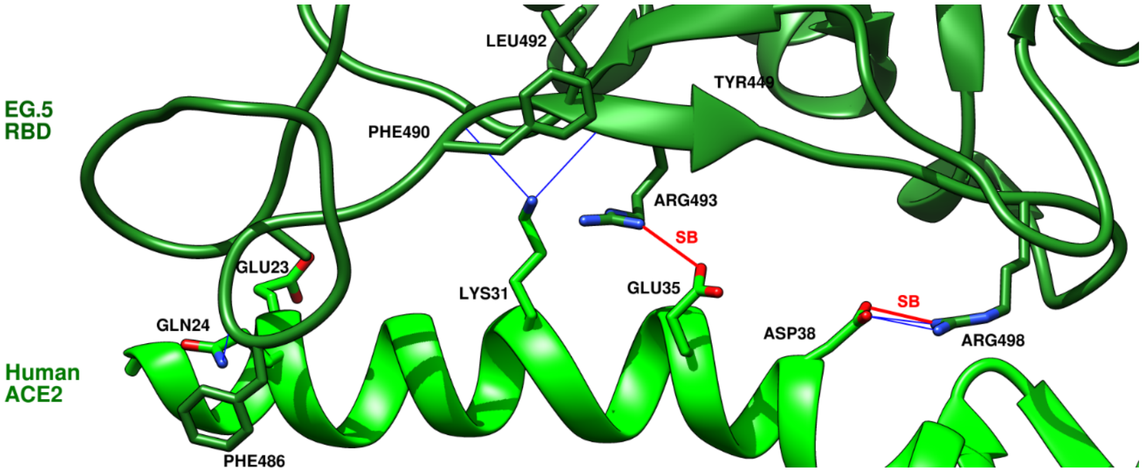

Figure S8: Representative Tridimensional structure of human ACE2 (in green) interface with a) minimum 1 (M1) and b) minimum 2 (M2) of EG.5 RBD domain (in Dark Green). Salt bridges (SB) are highlighted in red.

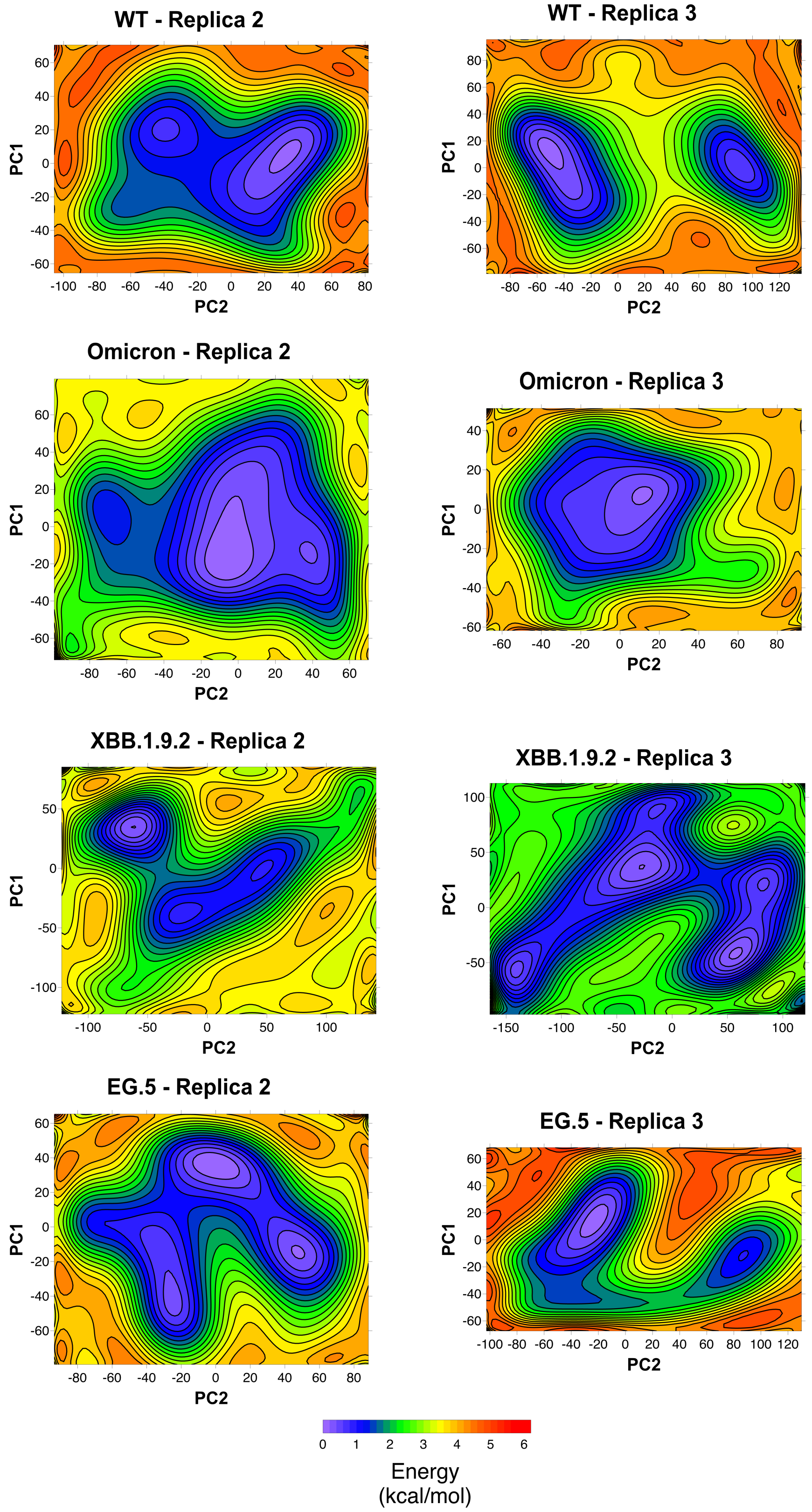

Figure S9: FEL analysis of native systems a) SARS-CoV-2, b) Omicron, c) XBB.1.9.1, and d) EG.5. The structures of each representative of the minima highlighting the distances that exist between the salt bridges (SB) are highlighted in red, e) M1 SARS-CoV-2, f) M1 Omicron, g) M1 XBB.1.9.1, and h) M1 EG.5, where minimum 1 (M1) and minimum 2 (M2) are two distinct conformational states. IN1 and IN2 are intermediate states.

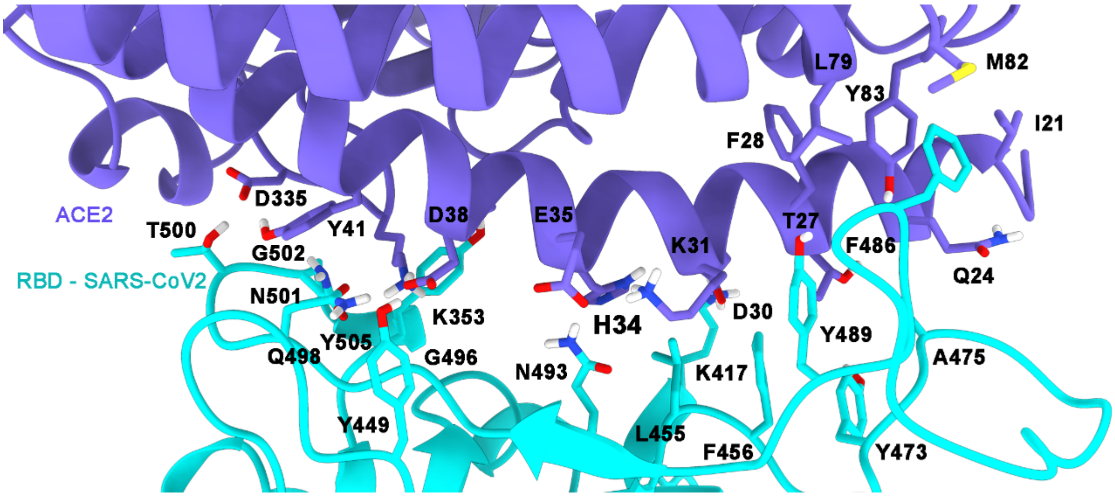

Figure S10: Representative tridimensional structure of ACE2 (in purple) and SARS-CoV-2 RBD (in cyan) depicting the location of key residues identified by IFP during the simulation.

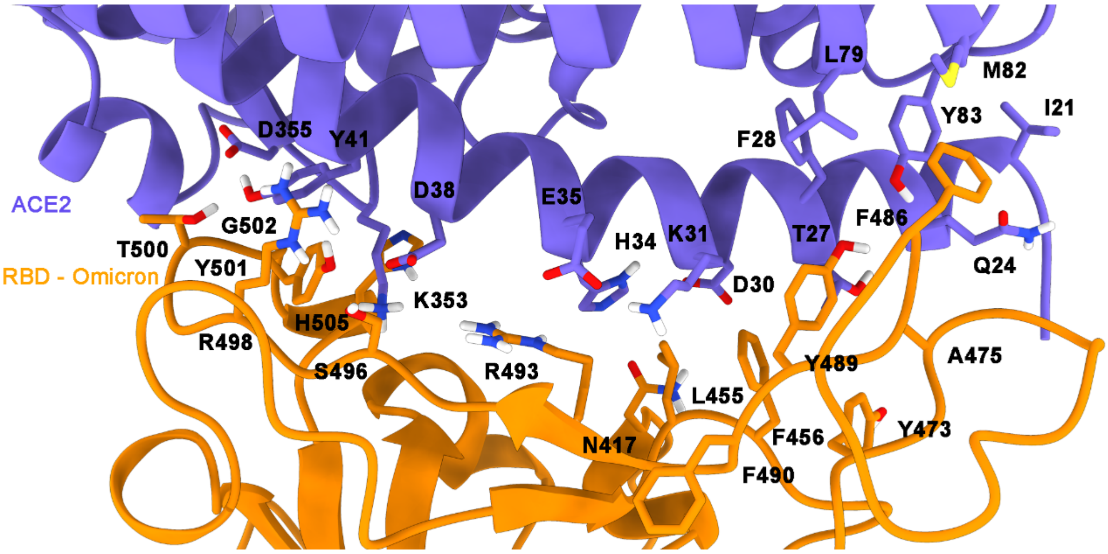

Figure S11: Representative tridimensional structure of ACE2 (in purple) and Omicron RBD (in orange) depicting the location of key residues identified by IFP during the simulation.

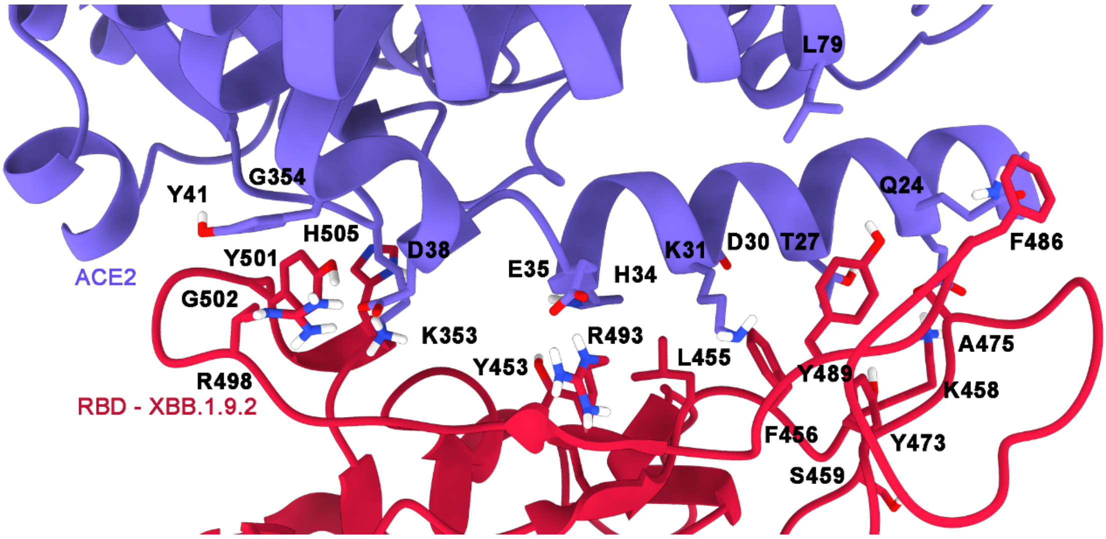

Figure S12: Representative Tridimensional structure of ACE2 (in purple) and XBB.1.9.2 RBD (in red) depicting the location of key residues identified by IFP during the simulation.

Table S3: Protein-protein interaction fingerprints (IFP) between the RBD of different SARS-CoV-2 variants (WT, Omicron, XBB.1.9.2 and EG.5) and the human ACE2 receptor. The occupancy of each interaction throughout the simulation is also provided.

WT			Omicron			XBB.1.9.2			EG.5		
RBD	Binding	ACE2	RBD	Binding	ACE2	RBD	Binding	ACE2	RBD	Binding	ACE2
	Hbond			Hbond			Hbond			Hbond	
Tyr449	0.77	Asp38	Arg486	0.68	Gln24	Lys458	0.57	Glu23	Tyr449	0.70	Asp38
Gln493	0.66	Glu35	Arg493	0.78	Glu35	Arg493	0.69	Glu35	Phe486	0.55	Gln24
Gln498	0.55	Asp38	Ser496	0.74	Asp38	Arg498	0.66	Asp38	Phe490	0.59	Lys31
Tyr505	0.69	Glu37	Arg498	0.84	Asp38	Gly502	0.55	Lys353	Arg493	0.56	Lys31
Gln498	0.71	Lys353	Thr500	0.89	Asp355				Arg493	0.82	Glu35
Thr500	0.61	Asp355	Gly502	0.74	Lys371				Ser496	0.87	Asp38
Gly502	0.95	Lys353							Arg498	0.84	Asp38
									THR500	0.91	ASP355
									GLY502	0.95	LYS353
RBD	VdW	ACE2	RBD	VdW	ACE2	RBD	VdW	ACE2	RBD	VdW	ACE2
Tyr449	0.81	Asp38	Arg486	0.77	Gln24	Lys458	0.65	Glu23	Tyr449	0.74	Asp38
Phe486	0.57	Thr27	Arg486	0.69	Tyr83	Arg493	0.58	Lys31	Tyr453	0.61	His34
Phe456	0.70	Gln24	Tyr489	0.51	Thr27	Arg493	0.70	Glu35	Phe486	0.67	Gln24
Phe456	0.68	Tyr83	Arg490	0.62	Lys31	Arg498	0.66	Asp38	Phe486	0.71	Tyr83
Tyr489	0.51	Thr27	Arg493	0.80	Glu35	Tyr501	0.61	Lys353	Phe490	0.79	Lys31
Gln493	0.59	Lys31	Ser496	0.78	Asp38	Gly502	0.59	Lys353	Arg493	0.69	Lys31
Gln493	0.70	Glu35	Arg498	0.85	Asp38	Gly504	0.53	Gly354	Arg493	0.85	Glu35
Gly496	0.93	Lys353	Arg498	0.67	Tyr41	His505	0.73	Lys353	Ser496	0.88	Asp38
Gln498	0.56	Asp38	Thr500	0.92	Asp355				Arg498	0.84	Asp38
Gln498	0.93	Lys353	Tyr501	0.67	Lys353				Arg498	0.65	Tyr41
Thr500	0.62	Tyr23	Gly502	0.76	Lys353				Thr500	0.53	Tyr41
Thr500	0.71	Asp355	His505	0.73	Lys353				Thr500	0.94	Asp355
Asn501	0.71	Lys353							Tyr501	0.76	Lys353
Gly502	0.96	Lys353							Gly502	0.97	Lys353
Tyr505	0.73	Glu37							His505	0.76	Lys353
Tyr505	0.77	Lys353									
RBD	Hydrophobic	ACE2	RBD	Hydrophobic	ACE2	RBD	Hydrophobic	ACE2	RBD	Hydrophobic	ACE2
Leu455	0.67	Lys31	Leu455	0.73	His34	Tyr453	0.50	His34	Tyr453	0.54	His34
Leu455	0.87	His34	Phe456	0.96	Thr27	Leu455	0.87	His34	Leu455	0.77	Lys31
Phe456	0.98	Thr27	Phe456	0.60	Lys31	Phe456	0.96	Thr27	Leu455	0.66	His34
Phe456	0.72	Asp30	Tyr473	0.77	Thr27	Phe456	0.71	Asp30	Leu456	0.88	Thr27
Phe456	0.78	Lys31	Ala475	0.81	Gln24	Phe456	0.80	Lys31	Ala475	0.83	Gln24
Tyr473	0.94	Thr27	Phe486	0.57	Ile21	Tyr473	0.81	Thr27	Phe486	0.50	Ile21
Ala475	0.84	Gln24	Phe486	0.71	Leu79	Ala475	0.70	Gln24	Phe486	0.68	Leu79
Ala475	0.57	Thr27	Phe486	0.85	Met82	Ala475	0.50	Thr27	Phe486	0.93	Met82
Phe486	0.50	Ile21	Phe486	0.86	Tyr83	Phe486	0.57	Leu79	Phe486	0.94	Tyr83
Phe486	0.68	Leu79	Tyr489	0.97	Thr27	Tyr489	0.86	Thr27	Tyr489	0.98	Thr27
Phe486	0.88	Met82	Tyr489	0.79	Phe28	Tyr489	0.83	Lys31	Tyr489	0.73	Phe28
Phe486	0.91	Tyr83	Tyr489	0.77	Lys31	Arg493	0.70	His34	Tyr489	0.81	Lys31
Tyr489	0.91	Thr27	Tyr501	0.91	Tyr41	Tyr501	0.82	Tyr41	Arg493	0.68	His34
Tyr489	0.71	Phe28	Tyr501	0.86	Lys353	Tyr501	0.80	Lys353	Tyr501	0.98	Tyr41
Tyr489	0.57	Lys31	His505	0.93	Lys353	His505	0.92	Lys353	Tyr501	0.99	Lys353
Gln498	0.92	Tyr41							His505	0.98	Lys353
Tyr505	0.99	Lys353									
RBD	Cationic	ACE2	RBD	Cationic	ACE2	RBD	Cationic	ACE2	RBD	Cationic	ACE2
Lys417	0.519,	Asp30	Arg493	0.82	Glu35	Lys458	0.67	Glu23	Arg493	0.92	Glu35
			Arg498	0.86	Asp38	Arg493	0.78	Glu35	Arg498	0.86	Asp38
						Arg498	0.71	Asp38			

Table S4: Per-residue energy decomposition (Van der Waals and electrostatic components, in kcal/mol) for the WT, Omicron, XBB.1.9.2 and EG.5 variants. The listed residues correspond to the interaction interface between the spike protein and the ACE2 receptor.

WT			Omicron			Xbb.1.9.2			EG.5		
**Residuos**	**Vdw**	**Elec**	**Residuos**	**Vdw**	**Elec**	**Residuos**	**Vdw**	**Elec**	**Residuos**	**Vdw**	**Elec**
**ACE2**											
Ser19	-0.10	14.45	Ser19	-0.09	36.74	Ser19	-0.14	36.18	Ser19	-0.10	37.57
Thr20	-1.23	-0.20	Thr20	-0.96	-0.73	Thr20	-1.02	-0.49	Thr20	-1.48	-0.19
Ile21	-0.56	-0.12	Ile21	-0.38	-0.95	Ile21	-0.73	-0.76	Ile21	-0.63	-0.49
Glu22	-0.13	-15.56	Glu22	-0.12	-36.05	Glu22	-0.13	-35.62	Glu22	-0.14	-36.82
Glu23	-1.21	-25.28	Glu23	-1.16	-48.30	Glu23	-1.06	-45.92	Glu23	-1.21	-50.27
Gln24	-4.32	-5.11	Gln24	-3.53	-4.86	Gln24	-3.89	-6.15	Gln24	-4.35	-6.43
Ala25	-0.31	-0.22	Ala25	-0.28	-0.87	Ala25	-0.31	-0.86	Ala25	-0.33	-0.83
Lys26	-0.42	23.94	Lys26	-0.40	40.92	Lys26	-0.44	40.08	Lys26	-0.33	42.37
Thr27	-3.57	-2.84	Thr27	-3.38	-1.56	Thr27	-3.34	-2.74	Thr27	-3.02	-2.63
Phe28	-1.88	-0.80	Phe28	-1.90	-1.00	Phe28	-1.95	-1.31	Phe28	-1.87	-1.74
Leu29	-0.24	-0.25	Leu29	-0.24	-1.05	Leu29	-0.23	-1.00	Leu29	-0.20	-1.18
Asp30	-1.44	-43.54	Asp30	-1.53	-52.20	Asp30	-1.11	-61.06	Asp30	-0.79	-67.87
Lys31	-3.19	9.40	Lyn31	-4.00	-5.18	Lyn31	-4.28	-4.05	Lyn31	-3.80	-5.21
Phe32	-0.25	-0.30	Phe32	-0.28	-1.97	Phe32	-0.41	-1.69	Phe32	-0.23	-1.77
Asn33	-0.25	0.68	Asn33	-0.25	-0.34	Asn33	-0.28	-0.80	Asn33	-0.20	0.72
Hid34	-3.29	-3.01	Hid34	-3.74	-2.52	Hid34	-3.18	-2.96	Hid34	-3.42	-1.08
Glu35	-1.03	-22.98	Glu35	-0.55	-90.56	Glu35	-0.95	-91.44	Glu35	-0.61	-80.76
Ala36	-0.10	0.20	Ala36	-0.14	-0.42	Ala36	-0.18	-0.33	Ala36	-0.09	-0.14
Glu37	-0.46	-32.60	Glu37	-0.70	-67.95	Glu37	-0.53	-58.49	Glu37	-0.37	-63.27
Asp38	-0.92	-29.53	Asp38	-0.48	-104.83	Asp38	-0.36	-104.25	Asp38	-0.19	-103.13
Leu39	-0.16	-0.04	Leu39	-0.27	0.20	Leu39	-0.46	0.04	Leu39	-0.26	-0.18
Phe40	-0.09	0.36	Phe40	-0.13	0.98	Phe40	-0.11	0.89	Phe40	-0.11	0.95
Tyr41	-2.37	-0.19	Tyr41	-3.60	0.98	Tyr41	-2.70	1.16	Tyr41	-3.59	1.05
Gln42	-0.75	-0.52	Gln42	-1.27	1.53	Gln42	-1.44	0.18	Gln42	-1.07	-1.40
Ser43	-0.04	0.29	Ser43	-0.05	0.93	Ser43	-0.06	1.09	Ser43	-0.05	0.86
Ser44	-0.05	0.88	Ser44	-0.06	2.38	Ser44	-0.06	2.21	Ser44	-0.06	2.52
Leu45	-0.70	0.63	Leu45	-0.94	2.27	Leu45	-0.87	2.04	Leu45	-0.91	2.16
Ala46	-0.03	0.20	Ala46	-0.05	1.03	Ala46	-0.09	0.93	Ala46	-0.04	0.95
Ser47	-0.01	0.52	Ser47	-0.02	1.35	Ser47	-0.02	1.24	Ser47	-0.02	1.40
Trp48	-0.09	0.67	Trp48	-0.08	1.58	Trp48	-0.10	1.61	Trp48	-0.10	1.62
Asn49	-0.04	0.48	Asn49	-0.06	1.66	Asn49	-0.14	1.48	Asn49	-0.07	1.64
Tyr50	-0.01	0.22	Tyr50	-0.01	0.68	Tyr50	-0.02	0.64	Tyr50	-0.01	0.72
Asn51	-0.01	0.67	Asn51	-0.01	1.53	Asn51	-0.01	1.31	Asn51	-0.01	1.63
Thr52	-0.01	0.18	Thr52	-0.01	0.62	Thr52	-0.02	0.53	Thr52	-0.01	0.56
Asn53	-0.01	0.17	Asn53	-0.01	0.24	Asn53	-0.05	0.78	Asn53	-0.01	0.08
Ile54	0.00	0.10	Ile54	0.00	0.23	Ile54	-0.01	0.09	Ile54	0.00	0.26
Thr55	0.00	-0.22	Thr55	0.00	-0.59	Thr55	-0.03	-0.10	Thr55	0.00	-0.61
Glu56	0.00	-11.10	Glu56	0.00	-28.38	Glu56	-0.03	-31.05	Glu56	0.00	-28.27
Glu57	-0.01	-13.70	Glu57	-0.01	-35.50	Glu57	-0.33	-41.94	Glu57	-0.01	-34.96
Asn58	-0.01	-0.49	Asn58	-0.01	-1.67	Asn58	-0.07	-1.18	Asn58	-0.01	-1.69
Val59	0.00	-0.22	Val59	0.00	-0.73	Val59	-0.03	-0.61	Val59	0.00	-0.73

Gln60	0.00	-0.07	Gln60	-0.01	-0.52	Gln60	-0.46	-0.84	Gln60	-0.01	-0.48
Asn61	-0.02	-0.14	Asn61	-0.05	-1.25	Asn61	-0.60	-2.01	Asn61	-0.04	-1.22
Met62	-0.01	-0.19	Met62	-0.01	-0.83	Met62	-0.05	-0.84	Met62	-0.01	-0.88
Asn63	0.00	-0.01	Asn63	-0.01	-0.46	Asn63	-0.05	-0.45	Asn63	-0.01	-0.43
Asn64	-0.01	0.06	Asn64	-0.02	-0.60	Asn64	-0.64	-0.93	Asn64	-0.02	-0.56
Ala65	-0.02	0.06	Ala65	-0.03	-0.35	Ala65	-0.13	-0.20	Ala65	-0.03	-0.36
Gly66	-0.01	-0.06	Gly66	-0.01	-0.57	Gly66	-0.02	-0.40	Gly66	-0.01	-0.53
Asp67	-0.01	-12.05	Asp67	-0.01	-32.90	Asp67	-0.04	-39.37	Asp67	-0.01	-37.71
Lys68	-0.04	16.26	Lys68	-0.05	42.97	Lys68	-0.45	50.02	Lys68	-0.07	49.55
Trp69	-0.04	0.00	Trp69	-0.05	-0.81	Trp69	-0.08	-0.75	Trp69	-0.04	-0.92
Ser70	-0.01	0.14	Ser70	-0.01	0.04	Ser70	-0.01	0.04	Ser70	-0.01	0.03
Ala71	-0.01	0.33	Ala71	-0.01	0.31	Ala71	-0.03	0.24	Ala71	-0.01	0.25
Phe72	-0.08	0.46	Phe72	-0.12	0.47	Phe72	-0.36	0.30	Phe72	-0.10	0.43
Leu73	-0.02	0.22	Leu73	-0.02	-0.02	Leu73	-0.04	-0.11	Leu73	-0.02	-0.18
Lys74	-0.02	9.71	Lys74	-0.02	30.62	Lys74	-0.03	35.94	Lys74	-0.02	35.10
Glu75	-0.09	-8.10	Glu75	-0.20	-37.41	Glu75	-0.37	-47.73	Glu75	-0.08	-45.27
Gln76	-0.16	0.06	Gln76	-0.23	-0.26	Gln76	-0.53	-0.34	Gln76	-0.16	-0.15
Ser77	-0.02	0.21	Ser77	-0.02	0.36	Ser77	-0.04	0.37	Ser77	-0.02	0.39
Thr78	-0.06	0.12	Thr78	-0.06	0.52	Thr78	-0.16	0.76	Thr78	-0.06	0.78
Leu79	-1.43	0.04	Leu79	-1.32	0.35	Leu79	-2.04	0.48	Leu79	-1.51	0.57
Ala80	-0.09	0.08	Ala80	-0.07	0.18	Ala80	-0.09	0.29	Ala80	-0.09	0.21
Gln81	-0.05	0.14	Gln81	-0.04	0.38	Gln81	-0.06	0.53	Gln81	-0.06	0.47
Met82	-1.13	0.04	Met82	-0.73	0.15	Met82	-1.45	0.02	Met82	-1.19	0.20
Tyr83	-1.94	-1.94	Tyr83	-1.28	-0.74	Tyr83	-1.97	-1.01	Tyr83	-2.06	-1.56
Pro84	-0.14	-0.26	Pro84	-0.08	-0.27	Pro84	-0.29	-0.20	Pro84	-0.18	-0.24
Leu85	-0.02	-0.20	Leu85	-0.01	-0.11	Leu85	-0.02	-0.01	Leu85	-0.02	-0.11
Gln86	-0.01	-0.15	Gln86	-0.01	0.07	Gln86	-0.01	0.14	Gln86	-0.01	0.14
Glu87	-0.03	-10.66	Glu87	-0.02	-28.96	Glu87	-0.04	-32.10	Glu87	-0.03	-33.75
Ile88	-0.04	0.32	Ile88	-0.03	0.57	Ile88	-0.05	0.59	Ile88	-0.04	0.60
Gln89	-0.01	0.21	Gln89	-0.01	0.59	Gln89	-0.01	0.58	Gln89	-0.01	0.64
Asn90	-0.02	0.03	Asn90	-0.02	-0.32	Asn90	-0.02	-0.52	Asn90	-0.02	-0.28
Leu91	-0.01	-0.03	Leu91	-0.01	-0.45	Leu91	-0.01	-0.54	Leu91	-0.01	-0.49
Thr92	-0.02	0.10	Thr92	-0.02	-0.20	Thr92	-0.02	-0.23	Thr92	-0.02	-0.18
Val93	-0.04	0.04	Val93	-0.04	-0.25	Val93	-0.04	-0.34	Val93	-0.04	-0.27
Lys94	-0.01	11.66	Lys94	-0.01	26.26	Lys94	-0.01	29.37	Lys94	-0.01	29.96
Leu95	-0.01	0.30	Leu95	-0.01	-0.05	Leu95	-0.01	-0.16	Leu95	-0.01	-0.05
Gln96	-0.04	-0.11	Gln96	-0.04	-0.83	Gln96	-0.03	-1.17	Gln96	-0.03	-0.97
Leu97	-0.05	0.12	Leu97	-0.04	-0.08	Leu97	-0.06	-0.19	Leu97	-0.05	-0.10
Gln98	-0.01	0.15	Gln98	-0.01	-0.05	Gln98	-0.01	-0.09	Gln98	-0.01	-0.07
Ala99	-0.01	0.28	Ala99	-0.01	0.24	Ala99	-0.01	0.20	Ala99	-0.01	0.30
Leu100	-0.03	0.24	Leu100	-0.03	0.37	Leu100	-0.05	0.35	Leu100	-0.03	0.44
Gln101	-0.02	0.28	Gln101	-0.02	0.72	Gln101	-0.02	0.65	Gln101	-0.02	0.81
Gln102	-0.01	0.03	Gln102	-0.01	-0.27	Gln102	-0.01	-0.14	Gln102	0.00	0.22
Asn103	-0.01	0.34	Asn103	-0.01	0.47	Asn103	-0.01	0.52	Asn103	-0.01	0.38
Gly104	0.00	0.18	Gly104	0.00	0.01	Gly104	0.00	0.05	Gly104	0.00	0.18
Ser105	0.00	0.22	Ser105	0.00	0.26	Ser105	0.00	0.21	Ser105	0.00	0.27
Ser106	-0.01	0.21	Ser106	-0.01	0.17	Ser106	-0.01	0.03	Ser106	0.00	0.08
Val107	0.00	0.17	Val107	-0.01	0.10	Val107	-0.01	0.06	Val107	0.00	0.21

Leu108	0.00	-0.06	Leu108	0.00	-0.53	Leu108	0.00	-0.51	Leu108	0.00	-0.57
Ser109	0.00	-0.13	Ser109	0.00	0.08	Ser109	0.00	0.23	Ser109	0.00	-0.06
Glu110	0.00	-9.19	Glu110	0.00	-27.35	Glu110	-0.01	-31.77	Glu110	0.00	-32.02
Asp111	0.00	-8.16	Asp111	0.00	-22.03	Asp111	0.00	-25.33	Asp111	0.00	-26.07
Lys112	0.00	7.72	Lys112	0.00	21.54	Lys112	0.00	24.85	Lys112	0.00	24.89
Ser113	0.00	-0.06	Ser113	0.00	0.28	Ser113	0.00	0.45	Ser113	0.00	0.20
Lys114	0.00	9.57	Lys114	0.00	24.75	Lys114	0.00	28.45	Lys114	0.00	30.30
Arg115	0.00	7.87	Arg115	0.00	19.77	Arg115	0.00	22.93	Arg115	0.00	23.73
Leu116	0.00	0.04	Leu116	0.00	0.30	Leu116	0.00	0.38	Leu116	0.00	0.29
Asn117	0.00	0.02	Asn117	0.00	0.31	Asn117	0.00	0.44	Asn117	0.00	0.25
Thr118	0.00	-0.07	Thr118	0.00	0.03	Thr118	0.00	0.12	Thr118	0.00	-0.08
Ile119	0.00	0.00	Ile119	0.00	0.17	Ile119	0.00	0.22	Ile119	0.00	0.13
Leu120	0.00	0.08	Leu120	0.00	0.34	Leu120	0.00	0.40	Leu120	0.00	0.43
Asn121	0.00	0.03	Asn121	0.00	0.28	Asn121	0.00	0.37	Asn121	0.00	0.21
Thr122	0.00	0.01	Thr122	0.00	0.16	Thr122	0.00	0.20	Thr122	0.00	0.13
Met123	0.00	0.11	Met123	0.00	0.37	Met123	0.00	0.40	Met123	0.00	0.41
Ser124	0.00	0.05	Ser124	0.00	0.27	Ser124	0.00	0.30	Ser124	0.00	0.38
Thr125	0.00	-0.03	Thr125	0.00	0.06	Thr125	0.00	0.10	Thr125	0.00	0.00
Ile126	0.00	0.02	Ile126	0.00	0.14	Ile126	0.00	0.17	Ile126	0.00	0.14
Tyr127	0.00	0.09	Tyr127	0.00	0.32	Tyr127	0.00	0.36	Tyr127	0.00	0.36
Ser128	0.00	0.02	Ser128	0.00	0.16	Ser128	0.00	0.19	Ser128	0.00	0.14
Thr129	0.00	0.06	Thr129	0.00	0.19	Thr129	0.00	0.22	Thr129	0.00	0.19
Gly130	0.00	0.03	Gly130	0.00	0.06	Gly130	0.00	0.09	Gly130	0.00	0.18
Lys131	0.00	8.15	Lys131	0.00	17.62	Lys131	0.00	18.51	Lys131	0.00	22.82
Val132	0.00	-0.04	Val132	0.00	-0.10	Val132	0.00	-0.05	Val132	0.00	-0.13
Cyx133	0.00	0.00	Cyx133	0.00	0.01	Cyx133	0.00	-0.02	Cyx133	0.00	-0.02
Asn134	0.00	0.03	Asn134	0.00	0.06	Asn134	0.00	0.02	Asn134	0.00	0.04
Pro135	0.00	0.00	Pro135	0.00	-0.02	Pro135	0.00	-0.04	Pro135	0.00	-0.12
Asp136	0.00	-5.97	Asp136	0.00	-13.04	Asp136	0.00	-15.96	Asp136	0.00	-16.73
Asn137	0.00	-0.04	Asn137	0.00	-0.09	Asn137	0.00	-0.04	Asn137	0.00	-0.23
Pro138	0.00	-0.05	Pro138	0.00	-0.09	Pro138	0.00	-0.06	Pro138	0.00	-0.22
Gln139	0.00	-0.05	Gln139	0.00	-0.11	Gln139	0.00	-0.01	Gln139	0.00	-0.15
Glu140	0.00	-7.10	Glu140	0.00	-15.10	Glu140	0.00	-18.11	Glu140	0.00	-19.61
Cyx141	0.00	-0.03	Cyx141	0.00	-0.06	Cyx141	0.00	-0.01	Cyx141	0.00	-0.15
Leu142	0.00	0.00	Leu142	0.00	-0.01	Leu142	0.00	-0.04	Leu142	0.00	-0.08
Leu143	0.00	0.10	Leu143	0.00	0.25	Leu143	0.00	0.24	Leu143	0.00	0.35
Leu144	0.00	0.07	Leu144	0.00	0.19	Leu144	0.00	0.21	Leu144	0.00	0.27
Glu145	0.00	-8.81	Glu145	0.00	-17.76	Glu145	0.00	-19.77	Glu145	0.00	-21.15
Pro146	0.00	0.08	Pro146	0.00	0.08	Pro146	0.00	0.18	Pro146	0.00	0.28
Gly147	0.00	0.06	Gly147	0.00	0.12	Gly147	0.00	0.17	Gly147	0.00	0.25
Leu148	0.00	0.07	Leu148	0.00	0.17	Leu148	0.00	0.19	Leu148	0.00	0.25
Asn149	0.00	0.15	Asn149	0.00	0.29	Asn149	0.00	0.33	Asn149	0.00	0.61
Glu150	0.00	-7.62	Glu150	0.00	-15.71	Glu150	0.00	-16.88	Glu150	0.00	-18.93
Ile151	0.00	0.06	Ile151	0.00	0.15	Ile151	0.00	0.16	Ile151	0.00	0.22
Met152	0.00	0.08	Met152	0.00	0.22	Met152	0.00	0.20	Met152	0.00	0.31
Ala153	0.00	0.07	Ala153	0.00	0.17	Ala153	0.00	0.18	Ala153	0.00	0.23
Asn154	0.00	0.09	Asn154	0.00	0.22	Asn154	0.00	0.22	Asn154	0.00	0.30
Ser155	0.00	-0.02	Ser155	0.00	0.01	Ser155	0.00	-0.02	Ser155	0.00	0.04

Leu156	0.00	0.00	Leu156	0.00	0.00	Leu156	0.00	0.01	Leu156	0.00	0.03
Asp157	0.00	-5.83	Asp157	0.00	-12.05	Asp157	0.00	-13.32	Asp157	0.00	-14.30
Tyr158	0.00	-0.08	Tyr158	0.00	-0.15	Tyr158	0.00	-0.16	Tyr158	0.00	-0.18
Asn159	0.00	-0.04	Asn159	0.00	-0.11	Asn159	0.00	-0.12	Asn159	0.00	-0.14
Glu160	0.00	-6.21	Glu160	0.00	-12.92	Glu160	0.00	-14.17	Glu160	0.00	-15.52
Arg161	0.00	6.56	Arg161	0.00	13.08	Arg161	0.00	14.48	Arg161	0.00	15.37
Leu162	0.00	-0.08	Leu162	0.00	-0.16	Leu162	0.00	-0.18	Leu162	0.00	-0.21
Trp163	0.00	-0.06	Trp163	0.00	-0.13	Trp163	0.00	-0.14	Trp163	0.00	-0.16
Ala164	0.00	-0.04	Ala164	0.00	-0.14	Ala164	0.00	-0.14	Ala164	0.00	-0.19
Trp165	0.00	-0.03	Trp165	0.00	-0.12	Trp165	0.00	-0.12	Trp165	0.00	-0.17
Glu166	0.00	-6.25	Glu166	0.00	-13.78	Glu166	0.00	-15.08	Glu166	0.00	-16.15
Ser167	0.00	-0.01	Ser167	0.00	-0.10	Ser167	0.00	-0.11	Ser167	0.00	-0.12
Trp168	0.00	-0.01	Trp168	0.00	-0.03	Trp168	0.00	-0.03	Trp168	0.00	0.02
Arg169	0.00	7.05	Arg169	0.00	14.85	Arg169	0.00	16.30	Arg169	0.00	17.66
Ser170	0.00	0.00	Ser170	0.00	-0.06	Ser170	0.00	-0.08	Ser170	0.00	-0.09
Glu171	0.00	-6.71	Glu171	0.00	-14.90	Glu171	0.00	-16.33	Glu171	0.00	-17.56
Val172	0.00	0.00	Val172	0.00	-0.04	Val172	0.00	-0.06	Val172	0.00	-0.07
Gly173	0.00	-0.05	Gly173	0.00	-0.13	Gly173	0.00	-0.16	Gly173	0.00	-0.21
Lys174	0.00	6.37	Lys174	0.00	13.99	Lys174	0.00	15.60	Lys174	0.00	16.92
Gln175	0.00	-0.03	Gln175	0.00	-0.13	Gln175	0.00	-0.15	Gln175	0.00	-0.17
Leu176	0.00	-0.02	Leu176	0.00	-0.12	Leu176	0.00	-0.15	Leu176	0.00	-0.16
Arg177	0.00	6.61	Arg177	0.00	15.27	Arg177	0.00	16.99	Arg177	0.00	18.03
Pro178	0.00	-0.03	Pro178	0.00	-0.15	Pro178	0.00	-0.18	Pro178	0.00	-0.15
Leu179	0.00	-0.01	Leu179	0.00	-0.15	Leu179	0.00	-0.18	Leu179	0.00	-0.14
Tyr180	0.00	-0.04	Tyr180	0.00	-0.15	Tyr180	0.00	-0.22	Tyr180	0.00	-0.14
Glu181	0.00	-6.89	Glu181	0.00	-16.35	Glu181	0.00	-18.26	Glu181	0.00	-19.35
Glu182	0.00	-7.57	Glu182	0.00	-18.69	Glu182	0.00	-20.95	Glu182	0.00	-22.43
Tyr183	0.00	-0.02	Tyr183	0.00	-0.13	Tyr183	0.00	-0.30	Tyr183	0.00	-0.19
Val184	0.00	-0.09	Val184	0.00	-0.31	Val184	0.00	-0.33	Val184	0.00	-0.32
Val185	0.00	-0.05	Val185	0.00	-0.21	Val185	0.00	-0.23	Val185	0.00	-0.19
Leu186	0.00	-0.03	Leu186	0.00	-0.25	Leu186	0.00	-0.28	Leu186	0.00	-0.22
Lys187	0.00	9.82	Lys187	0.00	22.97	Lys187	0.00	25.07	Lys187	0.00	27.54
Asn188	0.00	-0.07	Asn188	0.00	-0.51	Asn188	0.00	-0.49	Asn188	0.00	-0.51
Glu189	0.00	-7.74	Glu189	0.00	-21.16	Glu189	0.00	-23.92	Glu189	0.00	-24.33
Met190	0.00	-0.02	Met190	0.00	-0.28	Met190	0.00	-0.30	Met190	0.00	-0.18
Ala191	0.00	-0.08	Ala191	0.00	-0.36	Ala191	0.00	-0.41	Ala191	0.00	-0.32
Arg192	0.00	7.48	Arg192	0.00	19.79	Arg192	0.00	22.28	Arg192	0.00	22.51
Ala193	0.00	0.02	Ala193	0.00	-0.24	Ala193	0.00	-0.25	Ala193	0.00	-0.14
Asn194	0.00	-0.09	Asn194	0.00	-0.36	Asn194	0.00	-0.30	Asn194	0.00	-0.40
Hid195	0.00	0.04	Hid195	0.00	-0.11	Hid195	-0.01	0.03	Hid195	0.00	0.09
Tyr196	0.00	0.00	Tyr196	0.00	-0.10	Tyr196	0.00	-0.14	Tyr196	0.00	-0.14
Glu197	0.00	-7.50	Glu197	0.00	-19.28	Glu197	0.00	-21.45	Glu197	0.00	-21.46
Asp198	0.00	-8.26	Asp198	0.00	-19.50	Asp198	0.00	-21.67	Asp198	0.00	-22.09
Tyr199	0.00	-0.13	Tyr199	0.00	-0.25	Tyr199	0.00	-0.23	Tyr199	0.00	-0.28
Gly200	0.00	-0.16	Gly200	0.00	-0.24	Gly200	0.00	-0.25	Gly200	0.00	-0.25
Asp201	0.00	-8.74	Asp201	0.00	-20.61	Asp201	0.00	-22.96	Asp201	0.00	-23.07
Tyr202	0.00	-0.13	Tyr202	0.00	-0.20	Tyr202	0.00	-0.22	Tyr202	0.00	-0.12
Trp203	0.00	-0.20	Trp203	0.00	-0.16	Trp203	0.00	-0.22	Trp203	0.00	0.13

Arg204	0.00	8.35	Arg204	0.00	19.11	Arg204	0.00	22.12	Arg204	0.00	21.53
Gly205	0.00	-0.11	Gly205	0.00	-0.14	Gly205	0.00	-0.15	Gly205	0.00	-0.05
Asp206	0.00	-12.23	Asp206	0.00	-25.55	Asp206	0.00	-27.93	Asp206	0.00	-28.37
Tyr207	0.00	-0.11	Tyr207	0.00	-0.05	Tyr207	0.00	-0.06	Tyr207	0.00	0.04
Glu208	0.00	-10.03	Glu208	0.00	-22.79	Glu208	0.00	-25.10	Glu208	0.00	-24.73
Val209	0.00	0.19	Val209	0.00	0.32	Val209	0.00	0.35	Val209	0.00	0.32
Asn210	0.00	-0.12	Asn210	0.00	-0.27	Asn210	0.00	-0.25	Asn210	0.00	-0.04
Gly211	0.00	-0.02	Gly211	0.00	-0.03	Gly211	0.00	-0.07	Gly211	0.00	-0.07
Val212	0.00	0.14	Val212	0.00	0.11	Val212	0.00	0.14	Val212	0.00	0.07
Asp213	0.00	-8.97	Asp213	0.00	-19.08	Asp213	0.00	-20.77	Asp213	0.00	-20.87
Gly214	0.00	0.03	Gly214	0.00	0.10	Gly214	0.00	0.08	Gly214	0.00	-0.04
Tyr215	0.00	-0.04	Tyr215	0.00	-0.07	Tyr215	0.00	-0.09	Tyr215	0.00	0.03
Asp216	0.00	-8.96	Asp216	0.00	-19.97	Asp216	0.00	-21.97	Asp216	0.00	-21.22
Tyr217	0.00	-0.19	Tyr217	0.00	-0.39	Tyr217	0.00	-0.36	Tyr217	0.00	-0.40
Ser218	0.00	-0.02	Ser218	0.00	0.14	Ser218	0.00	0.07	Ser218	0.00	0.13
Arg219	0.00	9.22	Arg219	0.00	21.86	Arg219	0.00	22.55	Arg219	0.00	23.91
Gly220	0.00	0.04	Gly220	0.00	0.16	Gly220	0.00	0.13	Gly220	0.00	0.15
Gln221	0.00	0.07	Gln221	0.00	0.14	Gln221	0.00	0.10	Gln221	0.00	0.14
Leu222	0.00	0.11	Leu222	0.00	0.28	Leu222	0.00	0.28	Leu222	0.00	0.30
Ile223	0.00	0.04	Ile223	0.00	0.14	Ile223	0.00	0.15	Ile223	0.00	0.14
Glu224	0.00	-7.10	Glu224	0.00	-15.87	Glu224	0.00	-17.35	Glu224	0.00	-17.54
Asp225	0.00	-7.99	Asp225	0.00	-17.13	Asp225	0.00	-18.70	Asp225	0.00	-18.86
Val226	0.00	0.07	Val226	0.00	0.19	Val226	0.00	0.20	Val226	0.00	0.20
Glu227	0.00	-6.92	Glu227	0.00	-15.34	Glu227	0.00	-16.82	Glu227	0.00	-17.22
Hid228	0.00	0.12	Hid228	0.00	0.26	Hid228	0.00	0.30	Hid228	0.00	0.29
Thr229	0.00	0.06	Thr229	0.00	0.14	Thr229	0.00	0.16	Thr229	0.00	0.14
Phe230	0.00	0.04	Phe230	0.00	0.12	Phe230	0.00	0.12	Phe230	0.00	0.13
Glu231	0.00	-6.31	Glu231	0.00	-13.79	Glu231	0.00	-15.22	Glu231	0.00	-15.42
Glu232	0.00	-6.54	Glu232	0.00	-13.97	Glu232	0.00	-15.47	Glu232	0.00	-15.49
Ile233	0.00	0.07	Ile233	0.00	0.15	Ile233	0.00	0.16	Ile233	0.00	0.16
Lys234	0.00	6.22	Lys234	0.00	13.58	Lys234	0.00	14.95	Lys234	0.00	15.37
Pro235	0.00	0.03	Pro235	0.00	0.07	Pro235	0.00	0.07	Pro235	0.00	0.06
Leu236	0.00	0.08	Leu236	0.00	0.16	Leu236	0.00	0.17	Leu236	0.00	0.16
Tyr237	0.00	0.00	Tyr237	0.00	0.04	Tyr237	0.00	0.04	Tyr237	0.00	0.03
Glu238	0.00	-5.92	Glu238	0.00	-12.80	Glu238	0.00	-14.17	Glu238	0.00	-14.49
Hid239	0.00	0.11	Hid239	0.00	0.23	Hid239	0.00	0.23	Hid239	0.00	0.25
Leu240	0.00	0.07	Leu240	0.00	0.15	Leu240	0.00	0.16	Leu240	0.00	0.16
Hie241	0.00	0.08	Hie241	0.00	0.18	Hie241	0.00	0.20	Hie241	0.00	0.20
Ala242	0.00	0.03	Ala242	0.00	0.06	Ala242	0.00	0.06	Ala242	0.00	0.06
Tyr243	0.00	0.05	Tyr243	0.00	0.12	Tyr243	0.00	0.10	Tyr243	0.00	0.09
Val244	0.00	0.05	Val244	0.00	0.12	Val244	0.00	0.12	Val244	0.00	0.12
Arg245	0.00	5.62	Arg245	0.00	12.02	Arg245	0.00	13.43	Arg245	0.00	13.74
Ala246	0.00	0.04	Ala246	0.00	0.07	Ala246	0.00	0.08	Ala246	0.00	0.07
Lys247	0.00	5.75	Lys247	0.00	11.96	Lys247	0.00	13.38	Lys247	0.00	13.73
Leu248	0.00	0.04	Leu248	0.00	0.09	Leu248	0.00	0.09	Leu248	0.00	0.10
Met249	0.00	0.03	Met249	0.00	0.05	Met249	0.00	0.06	Met249	0.00	0.06
Asn250	0.00	0.05	Asn250	0.00	0.11	Asn250	0.00	0.10	Asn250	0.00	0.11
Ala251	0.00	0.04	Ala251	0.00	0.10	Ala251	0.00	0.10	Ala251	0.00	0.11

Tyr252	0.00	-0.01	Tyr252	0.00	-0.03	Tyr252	0.00	-0.02	Tyr252	0.00	-0.02
Pro253	0.00	-0.02	Pro253	0.00	-0.04	Pro253	0.00	-0.07	Pro253	0.00	-0.07
Ser254	0.00	0.01	Ser254	0.00	0.01	Ser254	0.00	0.02	Ser254	0.00	0.01
Tyr255	0.00	0.01	Tyr255	0.00	-0.01	Tyr255	0.00	0.02	Tyr255	0.00	-0.01
Ile256	0.00	0.05	Ile256	0.00	0.11	Ile256	0.00	0.10	Ile256	0.00	0.13
Ser257	0.00	-0.05	Ser257	0.00	-0.11	Ser257	0.00	-0.10	Ser257	0.00	-0.12
Pro258	0.00	-0.05	Pro258	0.00	-0.10	Pro258	0.00	-0.08	Pro258	0.00	-0.11
Ile259	0.00	-0.03	Ile259	0.00	-0.08	Ile259	0.00	-0.08	Ile259	0.00	-0.08
Gly260	0.00	-0.03	Gly260	0.00	-0.05	Gly260	0.00	-0.06	Gly260	0.00	-0.04
Cys261	0.00	0.04	Cys261	0.00	0.09	Cys261	0.00	0.10	Cys261	0.00	0.11
Leu262	0.00	-0.01	Leu262	0.00	-0.05	Leu262	0.00	-0.06	Leu262	0.00	-0.07
Pro263	0.00	-0.05	Pro263	0.00	-0.04	Pro263	0.00	-0.04	Pro263	0.00	-0.05
Ala264	0.00	-0.01	Ala264	0.00	0.00	Ala264	0.00	0.00	Ala264	0.00	-0.03
Hid265	0.00	-0.02	Hid265	0.00	0.01	Hid265	0.00	0.00	Hid265	0.00	-0.03
Leu266	0.00	-0.07	Leu266	0.00	-0.05	Leu266	0.00	-0.05	Leu266	0.00	-0.07
Leu267	0.00	0.02	Leu267	0.00	0.01	Leu267	0.00	0.02	Leu267	0.00	-0.01
Gly268	0.00	-0.01	Gly268	0.00	-0.09	Gly268	0.00	-0.12	Gly268	0.00	-0.12
Asp269	0.00	-7.69	Asp269	0.00	-15.40	Asp269	0.00	-16.94	Asp269	0.00	-18.32
Met270	0.00	0.10	Met270	0.00	0.18	Met270	0.00	0.21	Met270	0.00	0.23
Trp271	0.00	0.04	Trp271	0.00	0.05	Trp271	0.00	0.08	Trp271	0.00	0.09
Gly272	0.00	0.00	Gly272	0.00	0.04	Gly272	0.00	0.03	Gly272	0.00	0.09
Arg273	0.00	8.74	Arg273	0.00	17.06	Arg273	0.00	18.80	Arg273	0.00	20.13
Phe274	0.00	0.09	Phe274	0.00	0.10	Phe274	0.00	0.09	Phe274	0.00	0.09
Trp275	0.00	0.12	Trp275	0.00	0.19	Trp275	0.00	0.19	Trp275	0.00	0.22
Thr276	0.00	0.10	Thr276	0.00	0.16	Thr276	0.00	0.15	Thr276	0.00	0.22
Asn277	0.00	0.12	Asn277	0.00	0.18	Asn277	0.00	0.15	Asn277	0.00	0.19
Leu278	0.00	0.07	Leu278	0.00	0.11	Leu278	0.00	0.13	Leu278	0.00	0.13
Tyr279	0.00	0.09	Tyr279	0.00	0.14	Tyr279	0.00	0.16	Tyr279	0.00	0.20
Ser280	0.00	0.07	Ser280	0.00	0.12	Ser280	0.00	0.12	Ser280	0.00	0.16
Leu281	0.00	0.06	Leu281	0.00	0.12	Leu281	0.00	0.11	Leu281	0.00	0.14
Thr282	0.00	0.07	Thr282	0.00	0.02	Thr282	0.00	0.06	Thr282	0.00	0.11
Val283	0.00	0.05	Val283	0.00	0.09	Val283	0.00	0.05	Val283	0.00	0.00
Pro284	0.00	0.03	Pro284	0.00	0.04	Pro284	0.00	0.07	Pro284	0.00	-0.05
Phe285	0.00	-0.01	Phe285	0.00	0.01	Phe285	0.00	0.02	Phe285	0.00	-0.06
Gly286	0.00	-0.02	Gly286	0.00	-0.02	Gly286	0.00	-0.06	Gly286	0.00	-0.09
Gln287	0.00	-0.02	Gln287	0.00	-0.02	Gln287	0.00	-0.03	Gln287	0.00	-0.03
Lys288	0.00	6.35	Lys288	0.00	12.30	Lys288	0.00	14.14	Lys288	0.00	13.95
Pro289	0.00	0.01	Pro289	0.00	0.01	Pro289	0.00	0.02	Pro289	0.00	0.11
Asn290	0.00	-0.05	Asn290	0.00	0.00	Asn290	0.00	-0.03	Asn290	0.00	-0.04
Ile291	0.00	-0.07	Ile291	0.00	-0.09	Ile291	0.00	-0.12	Ile291	0.00	-0.14
Asp292	0.00	-7.50	Asp292	0.00	-15.24	Asp292	0.00	-17.23	Asp292	0.00	-17.41
Val293	0.00	0.00	Val293	0.00	0.03	Val293	0.00	0.01	Val293	0.00	0.00
Thr294	0.00	-0.13	Thr294	0.00	-0.19	Thr294	0.00	-0.22	Thr294	0.00	-0.25
Asp295	0.00	-7.30	Asp295	0.00	-14.30	Asp295	0.00	-16.38	Asp295	0.00	-16.45
Ala296	0.00	-0.01	Ala296	0.00	0.03	Ala296	0.00	0.00	Ala296	0.00	0.02
Met297	0.00	-0.05	Met297	0.00	-0.04	Met297	0.00	-0.06	Met297	0.00	-0.06
Val298	0.00	-0.06	Val298	0.00	-0.05	Val298	0.00	-0.09	Val298	0.00	-0.09
Asp299	0.00	-7.40	Asp299	0.00	-14.11	Asp299	0.00	-16.38	Asp299	0.00	-16.28

Gln300	0.00	-0.17	Gln300	0.00	-0.30	Gln300	0.00	-0.33	Gln300	0.00	-0.38
Ala301	0.00	-0.10	Ala301	0.00	-0.12	Ala301	0.00	-0.14	Ala301	0.00	-0.17
Trp302	0.00	0.01	Trp302	0.00	-0.05	Trp302	0.00	-0.14	Trp302	0.00	-0.08
Asp303	0.00	-10.78	Asp303	0.00	-19.92	Asp303	0.00	-23.13	Asp303	0.00	-23.47
Ala304	0.00	-0.04	Ala304	0.00	-0.05	Ala304	0.00	-0.01	Ala304	0.00	-0.05
Gln305	-0.01	-0.06	Gln305	0.00	-0.12	Gln305	0.00	-0.08	Gln305	0.00	-0.06
Arg306	0.00	9.58	Arg306	0.00	17.77	Arg306	0.00	20.33	Arg306	0.00	20.51
Ile307	0.00	0.03	Ile307	0.00	0.08	Ile307	0.00	0.11	Ile307	0.00	0.13
Phe308	-0.01	-0.02	Phe308	-0.01	-0.01	Phe308	-0.01	0.13	Phe308	-0.01	0.12
Lys309	-0.01	12.45	Lys309	0.00	22.83	Lys309	0.00	25.11	Lys309	0.00	26.06
Glu310	0.00	-10.14	Glu310	0.00	-18.63	Glu310	0.00	-20.97	Glu310	0.00	-21.27
Ala311	0.00	0.08	Ala311	0.00	0.16	Ala311	0.00	0.22	Ala311	0.00	0.27
Glu312	-0.01	-14.05	Glu312	-0.01	-24.63	Glu312	-0.01	-27.17	Glu312	-0.01	-28.25
Lys313	0.00	10.08	Lys313	0.00	18.65	Lys313	0.00	20.85	Lys313	0.00	21.27
Phe314	0.00	0.07	Phe314	0.00	0.18	Phe314	0.00	0.23	Phe314	0.00	0.23
Phe315	-0.01	0.11	Phe315	-0.01	0.22	Phe315	-0.01	0.28	Phe315	0.00	0.28
Val316	-0.01	0.03	Val316	0.00	0.10	Val316	0.00	0.13	Val316	0.00	0.15
Ser317	0.00	0.13	Ser317	0.00	0.25	Ser317	0.00	0.28	Ser317	0.00	0.31
Val318	0.00	0.07	Val318	0.00	0.14	Val318	0.00	0.21	Val318	0.00	0.17
Gly319	0.00	-0.12	Gly319	0.00	-0.07	Gly319	0.00	-0.02	Gly319	0.00	0.00
Leu320	-0.01	0.25	Leu320	-0.01	0.32	Leu320	-0.01	0.35	Leu320	-0.01	0.24
Pro321	-0.03	0.27	Pro321	-0.02	0.18	Pro321	-0.02	0.12	Pro321	-0.01	0.02
Asn322	-0.04	-0.62	Asn322	-0.03	-0.73	Asn322	-0.05	-0.92	Asn322	-0.02	-0.73
Met323	-0.11	0.00	Met323	-0.05	0.58	Met323	-0.06	-0.11	Met323	-0.11	0.51
Thr324	-0.29	0.04	Thr324	-0.38	0.58	Thr324	-0.29	-0.29	Thr324	-0.27	0.56
Gln325	-0.42	-0.26	Gln325	-0.19	-0.01	Gln325	-0.52	-0.56	Gln325	-0.48	-0.85
Gly326	-0.33	-0.06	Gly326	-0.29	0.10	Gly326	-0.14	-0.31	Gly326	-0.24	-0.03
Phe327	-0.24	-0.02	Phe327	-0.22	-0.20	Phe327	-0.24	-0.45	Phe327	-0.12	-0.30
Trp328	-0.05	0.13	Trp328	-0.05	0.16	Trp328	-0.03	-0.02	Trp328	-0.04	0.01
Glu329	-0.14	-17.99	Glu329	-0.12	-36.89	Glu329	-0.07	-39.75	Glu329	-0.13	-41.65
Asn330	-0.88	-0.30	Asn330	-0.76	-0.55	Asn330	-0.37	-0.82	Asn330	-0.84	-1.18
Ser331	-0.04	-0.06	Ser331	-0.03	-0.17	Ser331	-0.02	-0.20	Ser331	-0.03	-0.27
Met332	-0.02	0.07	Met332	-0.01	-0.05	Met332	-0.02	0.13	Met332	-0.02	0.07
Leu333	-0.01	0.06	Leu333	-0.01	0.31	Leu333	-0.01	0.04	Leu333	-0.01	-0.02
Thr334	0.00	-0.08	Thr334	0.00	0.11	Thr334	0.00	0.04	Thr334	0.00	0.12
Asp335	0.00	-10.87	Asp335	0.00	-23.55	Asp335	0.00	-25.97	Asp335	0.00	-25.17
Pro336	0.00	0.04	Pro336	0.00	0.07	Pro336	0.00	-0.15	Pro336	0.00	-0.10
Gly337	0.00	-0.02	Gly337	0.00	0.17	Gly337	0.00	-0.04	Gly337	0.00	-0.01
Asn338	0.00	-0.02	Asn338	0.00	0.21	Asn338	0.00	-0.24	Asn338	0.00	-0.27
Val339	0.00	0.04	Val339	0.00	0.05	Val339	0.00	0.03	Val339	0.00	0.18
Gln340	0.00	0.07	Gln340	0.00	0.23	Gln340	0.00	0.35	Gln340	0.00	-0.15
Lys341	0.00	11.14	Lys341	0.00	22.88	Lys341	0.00	28.76	Lys341	0.00	25.49
Ala342	0.00	0.29	Ala342	0.00	0.50	Ala342	0.00	0.29	Ala342	0.00	0.31
Val343	0.00	-0.16	Val343	0.00	-0.21	Val343	0.00	-0.36	Val343	0.00	0.26
Cyx344	0.00	-0.05	Cyx344	0.00	0.06	Cyx344	0.00	0.27	Cyx344	0.00	0.60
Hid345	0.00	-0.25	Hid345	0.00	-0.41	Hid345	0.00	-0.43	Hid345	0.00	-0.48
Pro346	0.00	0.11	Pro346	0.00	0.05	Pro346	0.00	0.10	Pro346	0.00	0.11
Thr347	-0.01	-0.08	Thr347	-0.01	-0.30	Thr347	0.00	-0.30	Thr347	-0.01	-0.62

Ala348	-0.01	0.24	Ala348	-0.01	0.13	Ala348	-0.01	0.16	Ala348	-0.01	0.18
Trp349	-0.04	-0.41	Trp349	-0.04	-0.56	Trp349	-0.04	-0.42	Trp349	-0.04	-0.79
Ash350	-0.10	-0.73	Ash350	-0.10	-1.34	Ash350	-0.08	-1.56	Ash350	-0.08	-1.46
Leu351	-0.26	0.49	Leu351	-0.30	-0.10	Leu351	-0.23	-0.21	Leu351	-0.34	0.01
Gly352	-0.33	-0.60	Gly352	-0.54	-0.61	Gly352	-0.29	0.01	Gly352	-0.59	-0.92
Lys353	-4.36	15.30	Lys353	-4.63	48.47	Lys353	-2.42	59.91	Lys353	-4.10	62.26
Gly354	-1.81	-1.70	Gly354	-1.77	-0.86	Gly354	-0.82	-0.81	Gly354	-1.52	-0.52
Asp355	-1.93	-31.29	Asp355	-1.99	-54.03	Asp355	-1.15	-56.97	Asp355	-1.69	-63.50
Phe356	-0.59	-0.30	Phe356	-0.58	-0.65	Phe356	-0.37	-0.66	Phe356	-0.33	-0.70
Arg357	-0.75	23.49	Arg357	-0.64	45.40	Arg357	-0.41	50.15	Arg357	-0.73	53.32
Ile358	-0.02	-0.29	Ile358	-0.02	-0.57	Ile358	-0.02	-0.60	Ile358	-0.02	-0.68
Leu359	-0.01	0.01	Leu359	-0.01	0.10	Leu359	-0.01	0.14	Leu359	-0.01	0.17
Met360	0.00	-0.10	Met360	0.00	-0.46	Met360	0.00	-0.34	Met360	0.00	-0.23
Cyx361	0.00	0.03	Cyx361	0.00	0.03	Cyx361	0.00	0.02	Cyx361	0.00	0.02
Thr362	0.00	0.01	Thr362	0.00	0.05	Thr362	0.00	0.06	Thr362	0.00	0.03
Lys363	0.00	9.44	Lys363	0.00	19.38	Lys363	0.00	21.61	Lys363	0.00	22.07
Val364	0.00	0.16	Val364	0.00	0.21	Val364	0.00	0.29	Val364	0.00	0.39
Thr365	0.00	-0.17	Thr365	0.00	-0.27	Thr365	0.00	-0.32	Thr365	0.00	-0.33
Met366	0.00	-0.16	Met366	0.00	-0.28	Met366	0.00	-0.29	Met366	0.00	-0.32
Asp367	0.00	-9.55	Asp367	0.00	-19.16	Asp367	0.00	-21.95	Asp367	0.00	-22.10
Asp368	0.00	-10.42	Asp368	0.00	-20.53	Asp368	0.00	-23.30	Asp368	0.00	-23.64
Phe369	0.00	-0.23	Phe369	0.00	-0.38	Phe369	0.00	-0.42	Phe369	0.00	-0.43
Leu370	0.00	-0.21	Leu370	0.00	-0.33	Leu370	0.00	-0.37	Leu370	0.00	-0.38
Thr371	0.00	-0.16	Thr371	0.00	-0.32	Thr371	0.00	-0.33	Thr371	0.00	-0.37
Ala372	0.00	-0.27	Ala372	0.00	-0.45	Ala372	0.00	-0.48	Ala372	0.00	-0.52
Hid373	0.00	-0.26	Hid373	0.00	-0.34	Hid373	0.00	-0.37	Hid373	0.00	-0.36
Hie374	0.00	-0.29	Hip374	0.00	20.52	Hip374	0.00	25.20	Hip374	0.00	25.54
Glu375	-0.01	-13.74	Glu375	0.00	-25.90	Glu375	0.00	-29.54	Glu375	0.00	-29.77
Met376	-0.01	-0.45	Met376	-0.01	-0.58	Met376	-0.01	-0.60	Met376	-0.01	-0.64
Gly377	0.00	-0.32	Gly377	0.00	-0.41	Gly377	0.00	-0.44	Gly377	0.00	-0.48
Hid378	-0.01	-0.30	Hid378	-0.01	-0.04	Hid378	-0.01	-0.02	Hid378	-0.01	0.07
Ile379	-0.03	-0.45	Ile379	-0.03	-0.50	Ile379	-0.02	-0.56	Ile379	-0.02	-0.57
Gln380	-0.02	-0.87	Gln380	-0.02	-0.73	Gln380	-0.01	-0.83	Gln380	-0.01	-0.77
Tyr381	-0.02	-0.36	Tyr381	-0.02	-0.34	Tyr381	-0.01	-0.31	Tyr381	-0.01	-0.25
Asp382	-0.04	-20.16	Asp382	-0.04	-34.23	Asp382	-0.03	-39.11	Asp382	-0.02	-39.25
Met383	-0.32	-0.53	Met383	-0.22	-0.34	Met383	-0.12	-0.54	Met383	-0.06	-0.43
Ala384	-0.03	-0.57	Ala384	-0.04	-0.40	Ala384	-0.02	-0.36	Ala384	-0.01	-0.35
Tyr385	-0.05	-0.34	Tyr385	-0.07	-0.26	Tyr385	-0.04	-0.30	Tyr385	-0.03	-0.30
Ala386	-0.25	-0.71	Ala386	-0.43	-0.99	Ala386	-0.17	-0.91	Ala386	-0.08	-0.83
Ala387	-0.21	-0.90	Ala387	-0.50	-0.88	Ala387	-0.39	-0.89	Ala387	-0.11	-0.72
Gln388	-0.09	-0.85	Gln388	-0.15	-1.50	Gln388	-0.12	-1.21	Gln388	-0.06	-1.36
Pro389	-0.10	1.07	Pro389	-0.14	1.48	Pro389	-0.10	1.50	Pro389	-0.09	1.50
Phe390	-0.09	0.59	Phe390	-0.12	1.14	Phe390	-0.08	1.23	Phe390	-0.07	1.27
Leu391	-0.03	0.47	Leu391	-0.04	0.76	Leu391	-0.03	0.91	Leu391	-0.03	0.88
Leu392	-0.03	0.64	Leu392	-0.03	0.87	Leu392	-0.03	0.96	Leu392	-0.02	0.90
Arg393	-0.21	25.16	Arg393	-0.33	45.39	Arg393	-0.09	48.62	Arg393	-0.10	49.28
Asn394	-0.01	0.25	Asn394	-0.01	0.43	Asn394	-0.01	0.68	Asn394	-0.01	0.56
Gly395	0.00	0.12	Gly395	0.00	-0.17	Gly395	0.00	0.12	Gly395	0.00	0.08

Ala396	0.00	0.08	Ala396	0.00	-0.13	Ala396	0.00	-0.21	Ala396	0.00	-0.25
Asn397	0.00	-0.17	Asn397	0.00	0.01	Asn397	0.00	0.05	Asn397	0.00	-0.04
Glh398	0.00	-0.03	Glh398	0.00	-0.02	Glh398	0.00	-0.12	Glh398	0.00	-0.19
Gly399	0.00	-0.05	Gly399	0.00	0.01	Gly399	0.00	0.00	Gly399	0.00	-0.03
Phe400	0.00	0.04	Phe400	0.00	0.19	Phe400	0.00	0.18	Phe400	0.00	0.18
Hid401	-0.01	-0.26	Hip401	-0.01	25.48	Hip401	0.00	30.94	Hip401	0.00	31.66
Glu402	0.00	-11.96	Glu402	0.00	-23.26	Glu402	0.00	-25.78	Glu402	0.00	-26.44
Ala403	0.00	0.13	Ala403	0.00	0.22	Ala403	0.00	0.21	Ala403	0.00	0.21
Val404	0.00	0.25	Val404	0.00	0.34	Val404	0.00	0.35	Val404	0.00	0.34
Gly405	0.00	0.14	Gly405	0.00	0.22	Gly405	0.00	0.23	Gly405	0.00	0.24
Glu406	0.00	-9.96	Glu406	0.00	-20.07	Glu406	0.00	-22.58	Glu406	0.00	-22.87
Ile407	0.00	0.19	Ile407	0.00	0.30	Ile407	0.00	0.35	Ile407	0.00	0.34
Met408	0.00	0.21	Met408	0.00	0.44	Met408	0.00	0.47	Met408	0.00	0.58
Ser409	0.00	0.21	Ser409	0.00	0.21	Ser409	0.00	0.20	Ser409	0.00	0.20
Leu410	0.00	0.11	Leu410	0.00	0.22	Leu410	0.00	0.22	Leu410	0.00	0.23
Ser411	0.00	0.23	Ser411	0.00	0.41	Ser411	0.00	0.39	Ser411	0.00	0.44
Ala412	0.00	0.14	Ala412	0.00	0.26	Ala412	0.00	0.29	Ala412	0.00	0.28
Ala413	0.00	0.11	Ala413	0.00	0.20	Ala413	0.00	0.20	Ala413	0.00	0.20
Thr414	0.00	-0.03	Thr414	0.00	-0.06	Thr414	0.00	-0.08	Thr414	0.00	-0.14
Pro415	0.00	-0.07	Pro415	0.00	-0.10	Pro415	0.00	-0.11	Pro415	0.00	-0.12
Lys416	0.00	7.58	Lys416	0.00	14.45	Lys416	0.00	15.90	Lys416	0.00	16.00
Hid417	0.00	-0.06	Hip417	0.00	16.18	Hip417	0.00	19.52	Hip417	0.00	19.88
Leu418	0.00	-0.02	Leu418	0.00	-0.02	Leu418	0.00	-0.02	Leu418	0.00	-0.04
Lys419	0.00	7.02	Lys419	0.00	13.68	Lys419	0.00	15.36	Lys419	0.00	15.51
Ser420	0.00	0.09	Ser420	0.00	0.15	Ser420	0.00	0.14	Ser420	0.00	0.16
Ile421	0.00	0.01	Ile421	0.00	0.00	Ile421	0.00	0.00	Ile421	0.00	-0.01
Gly422	0.00	0.00	Gly422	0.00	-0.01	Gly422	0.00	-0.03	Gly422	0.00	-0.03
Leu423	0.00	0.10	Leu423	0.00	0.17	Leu423	0.00	0.19	Leu423	0.00	0.20
Leu424	0.00	-0.03	Leu424	0.00	-0.07	Leu424	0.00	-0.03	Leu424	0.00	-0.09
Ser425	0.00	-0.02	Ser425	0.00	-0.05	Ser425	0.00	-0.02	Ser425	0.00	-0.05
Pro426	0.00	0.02	Pro426	0.00	0.02	Pro426	0.00	0.01	Pro426	0.00	0.03
Asp427	0.00	-6.04	Asp427	0.00	-12.14	Asp427	0.00	-13.94	Asp427	0.00	-13.74
Phe428	0.00	-0.01	Phe428	0.00	0.00	Phe428	0.00	0.04	Phe428	0.00	0.00
Gln429	0.00	-0.03	Gln429	0.00	-0.08	Gln429	0.00	-0.05	Gln429	0.00	-0.03
Glu430	0.00	-6.10	Glu430	0.00	-12.81	Glu430	0.00	-13.99	Glu430	0.00	-14.36
Asp431	0.00	-6.34	Asp431	0.00	-12.52	Asp431	0.00	-14.05	Asp431	0.00	-14.02
Asn432	0.00	-0.07	Asn432	0.00	-0.14	Asn432	0.00	-0.19	Asn432	0.00	-0.10
Glu433	0.00	-5.89	Glu433	0.00	-12.04	Glu433	0.00	-13.56	Glu433	0.00	-13.57
Thr434	0.00	-0.06	Thr434	0.00	-0.12	Thr434	0.00	-0.10	Thr434	0.00	-0.11
Glu435	0.00	-6.89	Glu435	0.00	-13.62	Glu435	0.00	-15.45	Glu435	0.00	-15.52
Ile436	0.00	-0.08	Ile436	0.00	-0.16	Ile436	0.00	-0.16	Ile436	0.00	-0.18
Asn437	0.00	-0.07	Asn437	0.00	-0.18	Asn437	0.00	-0.13	Asn437	0.00	-0.18
Phe438	0.00	-0.03	Phe438	0.00	-0.09	Phe438	0.00	-0.09	Phe438	0.00	-0.13
Leu439	0.00	-0.07	Leu439	0.00	-0.16	Leu439	0.00	-0.17	Leu439	0.00	-0.20
Leu440	0.00	-0.06	Leu440	0.00	-0.15	Leu440	0.00	-0.15	Leu440	0.00	-0.17
Lys441	0.00	7.89	Lys441	0.00	15.53	Lys441	0.00	17.21	Lys441	0.00	17.73
Gln442	0.00	-0.07	Gln442	0.00	-0.21	Gln442	0.00	-0.10	Gln442	0.00	-0.10
Ala443	0.00	-0.07	Ala443	0.00	-0.15	Ala443	0.00	-0.17	Ala443	0.00	-0.19

Leu444	0.00	-0.03	Leu444	0.00	-0.09	Leu444	0.00	-0.09	Leu444	0.00	-0.12
Thr445	0.00	0.00	Thr445	0.00	-0.04	Thr445	0.00	-0.03	Thr445	0.00	-0.06
Ile446	0.00	-0.02	Ile446	0.00	-0.05	Ile446	0.00	-0.06	Ile446	0.00	-0.08
Val447	0.00	-0.01	Val447	0.00	-0.05	Val447	0.00	-0.04	Val447	0.00	-0.07
Gly448	0.00	0.04	Gly448	0.00	0.03	Gly448	0.00	0.04	Gly448	0.00	0.02
Thr449	0.00	-0.01	Thr449	0.00	0.03	Thr449	0.00	-0.02	Thr449	0.00	-0.03
Leu450	0.00	-0.04	Leu450	0.00	-0.13	Leu450	0.00	-0.13	Leu450	0.00	-0.16
Pro451	0.00	-0.05	Pro451	0.00	-0.16	Pro451	0.00	-0.17	Pro451	0.00	-0.18
Phe452	0.00	0.02	Phe452	0.00	-0.04	Phe452	0.00	-0.04	Phe452	0.00	-0.03
Thr453	0.00	-0.04	Thr453	0.00	-0.15	Thr453	0.00	-0.16	Thr453	0.00	-0.16
Tyr454	0.00	-0.04	Tyr454	0.00	-0.16	Tyr454	0.00	-0.18	Tyr454	0.00	-0.21
Met455	0.00	0.02	Met455	0.00	-0.01	Met455	0.00	-0.05	Met455	0.00	-0.04
Leu456	0.00	0.06	Leu456	0.00	0.03	Leu456	0.00	0.03	Leu456	0.00	0.04
Glu457	0.00	-8.86	Glu457	0.00	-19.57	Glu457	0.00	-21.46	Glu457	0.00	-22.55
Lys458	0.00	7.03	Lys458	0.00	15.67	Lys458	0.00	17.18	Lys458	0.00	17.73
Trp459	0.00	-0.03	Trp459	0.00	-0.15	Trp459	0.00	-0.16	Trp459	0.00	-0.16
Arg460	0.00	8.96	Arg460	0.00	19.80	Arg460	0.00	21.72	Arg460	0.00	22.82
Trp461	0.00	0.10	Trp461	0.00	0.12	Trp461	0.00	0.05	Trp461	0.00	0.14
Met462	0.00	0.04	Met462	0.00	0.00	Met462	0.00	-0.02	Met462	0.00	0.00
Val463	0.00	0.07	Val463	0.00	0.05	Val463	0.00	0.04	Val463	0.00	0.08
Phe464	0.00	0.07	Phe464	0.00	0.06	Phe464	0.00	0.03	Phe464	0.00	0.07
Lys465	0.00	7.21	Lys465	0.00	17.31	Lys465	0.00	19.29	Lys465	0.00	19.48
Gly466	0.00	0.06	Gly466	0.00	0.10	Gly466	0.00	0.07	Gly466	0.00	0.14
Glu467	0.00	-6.55	Glu467	0.00	-15.60	Glu467	0.00	-17.51	Glu467	0.00	-17.56
Ile468	0.00	-0.06	Ile468	0.00	-0.18	Ile468	0.00	-0.19	Ile468	0.00	-0.19
Pro469	0.00	-0.04	Pro469	0.00	-0.03	Pro469	0.00	-0.02	Pro469	0.00	-0.02
Lys470	0.00	6.62	Lys470	0.00	16.01	Lys470	0.00	17.94	Lys470	0.00	18.73
Asp471	0.00	-5.88	Asp471	0.00	-13.70	Asp471	0.00	-15.19	Asp471	0.00	-15.93
Gln472	0.00	-0.01	Gln472	0.00	0.01	Gln472	0.00	0.02	Gln472	0.00	0.02
Trp473	0.00	-0.03	Trp473	0.00	0.00	Trp473	0.00	0.02	Trp473	0.00	0.04
Met474	0.00	-0.04	Met474	0.00	-0.04	Met474	0.00	-0.06	Met474	0.00	-0.11
Lys475	0.00	5.78	Lys475	0.00	13.23	Lys475	0.00	14.70	Lys475	0.00	15.36
Lys476	0.00	6.20	Lys476	0.00	14.23	Lys476	0.00	15.78	Lys476	0.00	16.27
Trp477	0.00	-0.01	Trp477	0.00	-0.02	Trp477	0.00	-0.04	Trp477	0.00	-0.01
Trp478	0.00	-0.01	Trp478	0.00	0.03	Trp478	0.00	0.02	Trp478	0.00	0.02
Glu479	0.00	-5.92	Glu479	0.00	-13.33	Glu479	0.00	-14.84	Glu479	0.00	-15.31
Met480	0.00	0.02	Met480	0.00	0.06	Met480	0.00	0.06	Met480	0.00	0.07
Lys481	0.00	6.94	Lyn481	0.00	1.12	Lyn481	0.00	0.05	Lyn481	0.00	0.07
Arg482	0.00	5.81	Arg482	0.00	12.86	Arg482	0.00	14.33	Arg482	0.00	15.06
Glu483	0.00	-6.01	Glu483	0.00	-13.37	Glu483	0.00	-14.83	Glu483	0.00	-15.19
Ile484	0.00	0.03	Ile484	0.00	0.09	Ile484	0.00	0.10	Ile484	0.00	0.12
Val485	0.00	0.01	Val485	0.00	0.05	Val485	0.00	0.05	Val485	0.00	0.07
Gly486	0.00	0.01	Gly486	0.00	0.04	Gly486	0.00	0.05	Gly486	0.00	0.05
Val487	0.00	0.01	Val487	0.00	0.00	Val487	0.00	-0.01	Val487	0.00	0.02
Val488	0.00	-0.04	Val488	0.00	-0.07	Val488	0.00	-0.08	Val488	0.00	-0.07
Glu489	0.00	-6.04	Glu489	0.00	-12.97	Glu489	0.00	-14.32	Glu489	0.00	-15.09
Pro490	0.00	0.06	Pro490	0.00	0.12	Pro490	0.00	0.03	Pro490	0.00	0.18
Val491	0.00	-0.05	Val491	0.00	-0.11	Val491	0.00	-0.06	Val491	0.00	-0.14

Pro492	0.00	0.02	Pro492	0.00	0.04	Pro492	0.00	0.09	Pro492	0.00	0.01
Hip493	0.00	6.09	Hip493	0.00	13.83	Hip493	0.00	15.03	Hip493	0.00	15.81
Asp494	0.00	-5.88	Asp494	0.00	-13.60	Asp494	0.00	-15.18	Asp494	0.00	-15.81
Glu495	0.00	-6.21	Glu495	0.00	-14.19	Glu495	0.00	-15.72	Glu495	0.00	-16.28
Thr496	0.00	-0.06	Thr496	0.00	-0.16	Thr496	0.00	-0.17	Thr496	0.00	-0.20
Tyr497	0.00	-0.03	Tyr497	0.00	0.02	Tyr497	0.00	0.02	Tyr497	0.00	-0.08
Cys498	0.00	-0.08	Cys498	0.00	-0.11	Cys498	0.00	-0.09	Cys498	0.00	-0.22
Asp499	0.00	-7.23	Asp499	0.00	-15.43	Asp499	0.00	-17.01	Asp499	0.00	-18.54
Pro500	0.00	-0.12	Pro500	0.00	-0.14	Pro500	0.00	-0.13	Pro500	0.00	-0.29
Ala501	0.00	-0.12	Ala501	0.00	-0.20	Ala501	0.00	-0.21	Ala501	0.00	-0.31
Ser502	0.00	-0.07	Ser502	0.00	-0.11	Ser502	0.00	-0.14	Ser502	0.00	-0.27
Leu503	0.00	0.00	Leu503	0.00	-0.09	Leu503	0.00	-0.12	Leu503	0.00	-0.23
Phe504	0.00	-0.03	Phe504	0.00	-0.11	Phe504	0.00	-0.15	Phe504	0.00	-0.22
Hie505	0.00	0.02	Hie505	0.00	-0.05	Hie505	0.00	-0.12	Hie505	0.00	-0.18
Val506	0.00	-0.09	Val506	0.00	-0.28	Val506	0.00	-0.29	Val506	0.00	-0.31
Ser507	0.00	-0.05	Ser507	0.00	-0.20	Ser507	0.00	-0.21	Ser507	0.00	-0.17
Asn508	0.00	0.04	Asn508	0.00	-0.31	Asn508	0.00	-0.15	Asn508	0.00	-0.09
Asp509	0.00	-10.44	Asp509	0.00	-23.64	Asp509	0.00	-25.71	Asp509	0.00	-28.63
Tyr510	0.00	0.19	Tyr510	0.00	0.32	Tyr510	0.00	0.26	Tyr510	0.00	0.50
Ser511	0.00	-0.07	Ser511	0.00	0.06	Ser511	0.00	-0.01	Ser511	0.00	-0.13
Phe512	0.00	-0.06	Phe512	0.00	-0.02	Phe512	0.00	-0.03	Phe512	0.00	0.00
Ile513	0.00	0.01	Ile513	0.00	0.13	Ile513	0.00	0.14	Ile513	0.00	0.16
Arg514	0.00	11.70	Arg514	0.00	24.19	Arg514	0.00	26.39	Arg514	0.00	27.95
Tyr515	0.00	-0.02	Tyr515	0.00	0.08	Tyr515	0.00	0.05	Tyr515	0.00	0.13
Tyr516	0.00	0.02	Tyr516	0.00	0.14	Tyr516	0.00	0.11	Tyr516	0.00	0.15
Thr517	0.00	-0.04	Thr517	0.00	0.05	Thr517	0.00	0.05	Thr517	0.00	0.10
Arg518	0.00	10.58	Arg518	0.00	20.44	Arg518	0.00	22.77	Arg518	0.00	23.99
Thr519	0.00	-0.02	Thr519	0.00	0.08	Thr519	0.00	0.04	Thr519	0.00	0.09
Leu520	0.00	0.03	Leu520	0.00	0.14	Leu520	0.00	0.13	Leu520	0.00	0.16
Tyr521	0.00	0.03	Tyr521	0.00	0.09	Tyr521	0.00	0.13	Tyr521	0.00	0.19
Gln522	0.00	0.00	Gln522	0.00	-0.06	Gln522	0.00	-0.01	Gln522	0.00	0.00
Phe523	0.00	0.00	Phe523	0.00	0.03	Phe523	0.00	0.03	Phe523	0.00	0.06
Gln524	0.00	0.00	Gln524	0.00	-0.03	Gln524	0.00	0.03	Gln524	0.00	-0.01
Phe525	0.00	0.09	Phe525	0.00	0.20	Phe525	0.00	0.19	Phe525	0.00	0.24
Gln526	0.00	0.02	Gln526	0.00	-0.11	Gln526	0.00	-0.06	Gln526	0.00	0.03
Glu527	0.00	-7.86	Glu527	0.00	-15.76	Glu527	0.00	-17.50	Glu527	0.00	-17.31
Ala528	0.00	0.09	Ala528	0.00	0.18	Ala528	0.00	0.19	Ala528	0.00	0.20
Leu529	0.00	0.08	Leu529	0.00	0.16	Leu529	0.00	0.17	Leu529	0.00	0.19
Cyx530	0.00	0.01	Cyx530	0.00	0.04	Cyx530	0.00	0.06	Cyx530	0.00	0.04
Gln531	0.00	0.07	Gln531	0.00	0.13	Gln531	0.00	0.16	Gln531	0.00	0.15
Ala532	0.00	0.11	Ala532	0.00	0.20	Ala532	0.00	0.20	Ala532	0.00	0.22
Ala533	0.00	0.08	Ala533	0.00	0.14	Ala533	0.00	0.13	Ala533	0.00	0.14
Lys534	0.00	7.72	Lys534	0.00	15.07	Lys534	0.00	16.46	Lys534	0.00	16.43
Hie535	0.00	-0.02	Hie535	0.00	-0.03	Hie535	0.00	0.01	Hie535	0.00	-0.02
Glu536	0.00	-6.63	Glu536	0.00	-13.30	Glu536	0.00	-15.03	Glu536	0.00	-14.66
Gly537	0.00	-0.08	Gly537	0.00	-0.15	Gly537	0.00	-0.13	Gly537	0.00	-0.16
Pro538	0.00	-0.06	Pro538	0.00	-0.09	Pro538	0.00	-0.09	Pro538	0.00	-0.09
Leu539	0.00	-0.06	Leu539	0.00	-0.08	Leu539	0.00	-0.08	Leu539	0.00	-0.11

Hid540	0.00	-0.01	Hid540	0.00	-0.09	Hid540	0.00	-0.09	Hid540	0.00	-0.06
Lys541	0.00	6.66	Lys541	0.00	13.38	Lys541	0.00	14.94	Lys541	0.00	14.98
Cyx542	0.00	-0.06	Cyx542	0.00	-0.11	Cyx542	0.00	-0.12	Cyx542	0.00	-0.13
Asp543	0.00	-8.54	Asp543	0.00	-16.51	Asp543	0.00	-18.08	Asp543	0.00	-18.46
Ile544	0.00	-0.05	Ile544	0.00	-0.01	Ile544	0.00	-0.04	Ile544	0.00	0.04
Ser545	0.00	-0.03	Ser545	0.00	-0.21	Ser545	0.00	-0.24	Ser545	0.00	-0.23
Asn546	0.00	0.00	Asn546	0.00	-0.03	Asn546	0.00	0.02	Asn546	0.00	-0.05
Ser547	0.00	-0.16	Ser547	0.00	-0.22	Ser547	0.00	-0.21	Ser547	0.00	-0.31
Thr548	0.00	-0.13	Thr548	0.00	-0.21	Thr548	0.00	-0.07	Thr548	0.00	-0.21
Glu549	0.00	-9.51	Glu549	0.00	-17.45	Glu549	0.00	-19.55	Glu549	0.00	-18.74
Ala550	0.00	-0.16	Ala550	0.00	-0.23	Ala550	0.00	-0.27	Ala550	0.00	-0.26
Gly551	0.00	-0.22	Gly551	0.00	-0.30	Gly551	0.00	-0.34	Gly551	0.00	-0.35
Gln552	0.00	-0.30	Gln552	0.00	-0.40	Gln552	0.00	-0.41	Gln552	0.00	-0.38
Lys553	0.00	9.73	Lys553	0.00	18.15	Lys553	0.00	20.03	Lys553	0.00	19.66
Leu554	0.00	-0.20	Leu554	0.00	-0.23	Leu554	0.00	-0.29	Leu554	0.00	-0.31
Phe555	-0.01	-0.22	Phe555	-0.02	-0.35	Phe555	-0.01	-0.36	Phe555	-0.01	-0.40
Asn556	0.00	-0.50	Asn556	-0.01	-0.67	Asn556	0.00	-0.67	Asn556	0.00	-0.66
Met557	0.00	-0.24	Met557	-0.01	-0.56	Met557	0.00	-0.32	Met557	0.00	-0.44
Leu558	-0.01	-0.22	Leu558	-0.01	-0.46	Leu558	-0.01	-0.43	Leu558	-0.01	-0.50
Arg559	-0.03	15.93	Arg559	-0.04	25.84	Arg559	-0.03	28.93	Arg559	-0.01	27.50
Leu560	-0.01	-0.42	Leu560	-0.01	-0.66	Leu560	-0.01	-0.45	Leu560	-0.01	-0.39
Gly561	0.00	0.19	Gly561	-0.01	0.51	Gly561	0.00	0.48	Gly561	0.00	0.17
Lys562	-0.02	16.33	Lys562	-0.02	29.65	Lys562	-0.01	31.03	Lys562	-0.01	29.56
Ser563	-0.01	0.03	Ser563	-0.01	0.03	Ser563	-0.01	0.11	Ser563	-0.01	-0.03
Glu564	-0.01	-14.32	Glu564	-0.01	-25.17	Glu564	0.00	-27.08	Glu564	0.00	-26.66
Pro565	0.00	-0.03	Pro565	0.00	0.16	Pro565	0.00	0.16	Pro565	0.00	0.20
Trp566	0.00	0.11	Trp566	0.00	0.35	Trp566	0.00	0.35	Trp566	0.00	0.37
Thr567	0.00	-0.06	Thr567	0.00	-0.01	Thr567	0.00	0.04	Thr567	0.00	-0.02
Leu568	0.00	0.21	Leu568	0.00	0.35	Leu568	0.00	0.38	Leu568	0.00	0.36
Ala569	0.00	0.14	Ala569	0.00	0.25	Ala569	0.00	0.31	Ala569	0.00	0.30
Leu570	0.00	0.04	Leu570	0.00	0.09	Leu570	0.00	0.15	Leu570	0.00	0.14
Glu571	0.00	-9.13	Glu571	0.00	-18.69	Glu571	0.00	-19.89	Glu571	0.00	-20.08
Asn572	0.00	0.19	Asn572	0.00	0.40	Asn572	0.00	0.47	Asn572	0.00	0.42
Val573	0.00	0.16	Val573	0.00	0.26	Val573	0.00	0.30	Val573	0.00	0.29
Val574	0.00	0.13	Val574	0.00	0.24	Val574	0.00	0.23	Val574	0.00	0.25
Gly575	0.00	0.12	Gly575	0.00	0.18	Gly575	0.00	0.13	Gly575	0.00	0.17
Ala576	0.00	-0.13	Ala576	0.00	-0.19	Ala576	0.00	-0.19	Ala576	0.00	-0.20
Lys577	0.00	8.56	Lys577	0.00	18.23	Lys577	0.00	19.57	Lys577	0.00	19.80
Asn578	0.00	-0.06	Asn578	0.00	-0.05	Asn578	0.00	-0.08	Asn578	0.00	-0.03
Met579	0.00	0.06	Met579	0.00	0.09	Met579	0.00	0.15	Met579	0.00	0.12
Asn580	0.00	-0.01	Asn580	0.00	-0.02	Asn580	0.00	0.01	Asn580	0.00	-0.04
Val581	0.00	-0.03	Val581	0.00	-0.02	Val581	0.00	-0.03	Val581	0.00	-0.03
Arg582	0.00	7.02	Arg582	0.00	14.64	Arg582	0.00	16.08	Arg582	0.00	16.03
Pro583	0.00	0.10	Pro583	0.00	0.20	Pro583	0.00	0.21	Pro583	0.00	0.20
Leu584	0.00	0.05	Leu584	0.00	0.13	Leu584	0.00	0.14	Leu584	0.00	0.15
Leu585	0.00	0.03	Leu585	0.00	0.09	Leu585	0.00	0.08	Leu585	0.00	0.08
Asn586	0.00	0.09	Asn586	0.00	0.23	Asn586	0.00	0.18	Asn586	0.00	0.30
Tyr587	0.00	0.10	Tyr587	0.00	0.21	Tyr587	0.00	0.20	Tyr587	0.00	0.21

Phe588	0.00	0.07	Phe588	0.00	0.14	Phe588	0.00	0.15	Phe588	0.00	0.15
Glu589	0.00	-6.30	Glu589	0.00	-13.13	Glu589	0.00	-14.64	Glu589	0.00	-14.63
Pro590	0.00	0.09	Pro590	0.00	0.15	Pro590	0.00	0.17	Pro590	0.00	0.17
Leu591	0.00	0.10	Leu591	0.00	0.18	Leu591	0.00	0.19	Leu591	0.00	0.20
Phe592	0.00	0.06	Phe592	0.00	0.12	Phe592	0.00	0.13	Phe592	0.00	0.12
Thr593	0.00	0.07	Thr593	0.00	0.13	Thr593	0.00	0.13	Thr593	0.00	0.14
Trp594	0.00	0.04	Trp594	0.00	0.07	Trp594	0.00	0.08	Trp594	0.00	0.07
Leu595	0.00	0.07	Leu595	0.00	0.14	Leu595	0.00	0.15	Leu595	0.00	0.15
Lys596	0.00	5.77	Lys596	0.00	12.21	Lys596	0.00	13.42	Lys596	0.00	13.69
Asp597	0.00	-5.21	Asp597	0.00	-11.01	Asp597	0.00	-12.31	Asp597	0.00	-12.43
Gln598	0.00	0.08	Gln598	0.00	0.16	Gln598	0.00	0.16	Gln598	0.00	0.16
Asn599	0.00	0.05	Asn599	0.00	0.09	Asn599	0.00	0.11	Asn599	0.00	0.09
Lys600	0.00	5.04	Lys600	0.00	10.67	Lys600	0.00	11.90	Lys600	0.00	12.06
Asn601	0.00	0.01	Asn601	0.00	0.01	Asn601	0.00	0.02	Asn601	0.00	0.02
Ser602	0.00	0.01	Ser602	0.00	0.03	Ser602	0.00	0.02	Ser602	0.00	0.05
Phe603	0.00	-0.03	Phe603	0.00	-0.08	Phe603	0.00	-0.10	Phe603	0.00	-0.09
Val604	0.00	0.03	Val604	0.00	0.06	Val604	0.00	0.06	Val604	0.00	0.07
Gly605	0.00	0.04	Gly605	0.00	0.09	Gly605	0.00	0.08	Gly605	0.00	0.11
Trp606	0.00	-0.01	Trp606	0.00	-0.03	Trp606	0.00	0.01	Trp606	0.00	-0.02
Ser607	0.00	-0.05	Ser607	0.00	-0.10	Ser607	0.00	-0.07	Ser607	0.00	-0.11
Thr608	0.00	0.00	Thr608	0.00	0.01	Thr608	0.00	-0.01	Thr608	0.00	0.01
Asp609	0.00	-5.18	Asp609	0.00	-11.42	Asp609	0.00	-12.55	Asp609	0.00	-12.92
Trp610	0.00	-0.02	Trp610	0.00	-0.13	Trp610	0.00	-0.03	Trp610	0.00	-0.20
Ser611	0.00	0.01	Ser611	0.00	0.04	Ser611	0.00	0.01	Ser611	0.00	0.10
Pro612	0.00	0.05	Pro612	0.00	0.04	Pro612	0.00	0.04	Pro612	0.00	0.13
Tyr613	0.00	0.02	Tyr613	0.00	0.01	Tyr613	0.00	0.03	Tyr613	0.00	0.08
Ala614	0.00	0.01	Ala614	0.00	0.02	Ala614	0.00	0.02	Ala614	0.00	0.01
Asp615	0.00	-9.81	Asp615	0.00	-20.72	Asp615	0.00	-22.77	Asp615	0.00	-24.45
**Spike RBD**											
Thr333	0.00	-54.88	Thr333	0.00	-52.94	Thr333	0.00	-54.60	Thr333	0.00	-53.39
Asn334	0.00	0.07	Asn334	0.00	0.11	Asn334	0.00	0.17	Asn334	0.00	0.03
Leu335	0.00	-0.17	Leu335	0.00	-0.13	Leu335	0.00	-0.24	Leu335	0.00	-0.17
Cyx336	0.00	-0.03	Cyx336	0.00	-0.03	Cyx336	0.00	0.05	Cyx336	0.00	0.06
Pro337	0.00	0.17	Pro337	0.00	0.22	Pro337	0.00	0.22	Pro337	0.00	0.32
Phe338	0.00	0.37	Phe338	0.00	0.54	Phe338	0.00	0.41	Phe338	0.00	0.62
Gly339	0.00	0.69	Asp339	0.00	56.14	Asp339	0.00	67.12	Asp339	0.00	68.06
Glu340	0.00	66.82	Glu340	0.00	64.92	Glu340	0.00	64.38	Glu340	0.00	66.15
Val341	0.00	0.49	Val341	0.00	0.61	Val341	0.00	0.60	Val341	0.00	0.76
Phe342	0.00	0.69	Phe342	0.00	0.81	Phe342	0.00	0.60	Phe342	0.00	0.90
Asn343	0.00	0.79	Asn343	0.00	0.95	Asn343	0.00	1.33	Asn343	0.00	1.15
Ala344	0.00	0.56	Ala344	0.00	0.75	Ala344	0.00	0.86	Ala344	0.00	0.85
Thr345	0.00	0.67	Thr345	0.00	0.77	Thr345	0.00	0.82	Thr345	0.00	0.79
Arg346	-0.01	-77.76	Arg346	-0.01	-76.97	Arg346	0.00	-71.71	Arg346	-0.01	-80.26
Phe347	-0.01	0.21	Phe347	-0.01	0.33	Phe347	-0.01	0.39	Phe347	-0.01	0.53
Ala348	0.00	0.81	Ala348	0.00	0.58	Ala348	0.00	0.76	Ala348	0.00	0.66
Ser349	-0.01	-0.83	Ser349	-0.01	-1.12	Ser349	-0.01	-0.90	Ser349	-0.01	-1.01
Val350	-0.02	-1.30	Val350	-0.02	-1.41	Val350	-0.02	-1.30	Val350	-0.02	-1.52
Tyr351	-0.02	-0.93	Tyr351	-0.02	-1.10	Tyr351	-0.02	-0.81	Tyr351	-0.02	-1.22

Ala352	0.00	-0.42	Ala352	0.00	-0.61	Ala352	0.00	-0.30	Ala352	0.00	-0.57
Trp353	-0.01	-1.39	Trp353	-0.01	-1.40	Trp353	-0.01	-0.62	Trp353	-0.01	-1.25
Asn354	0.00	-0.11	Asn354	0.00	-0.27	Asn354	0.00	-0.95	Asn354	0.00	-0.27
Arg355	0.00	-69.42	Arg355	0.00	-68.65	Arg355	0.00	-70.59	Arg355	0.00	-67.41
Lys356	0.00	-67.60	Lys356	0.00	-65.72	Lys356	0.00	-66.02	Lys356	0.00	-66.47
Arg357	0.00	-60.31	Arg357	0.00	-59.14	Arg357	0.00	-59.50	Arg357	0.00	-58.63
Ile358	0.00	-0.04	Ile358	0.00	0.05	Ile358	0.00	-0.04	Ile358	0.00	0.00
Ser359	0.00	0.09	Ser359	0.00	0.17	Ser359	0.00	0.17	Ser359	0.00	0.16
Asn360	0.00	-0.11	Asn360	0.00	-0.04	Asn360	0.00	-0.10	Asn360	0.00	0.01
Cyx361	0.00	0.16	Cyx361	0.00	0.16	Cyx361	0.00	0.15	Cyx361	0.00	0.08
Val362	0.00	0.23	Val362	0.00	0.25	Val362	0.00	0.08	Val362	0.00	0.33
Ala363	0.00	-0.23	Ala363	0.00	-0.30	Ala363	0.00	-0.21	Ala363	0.00	-0.27
Asp364	0.00	64.40	Asp364	0.00	62.17	Asp364	0.00	62.54	Asp364	0.00	61.86
Tyr365	0.00	0.63	Tyr365	0.00	0.70	Tyr365	0.00	0.64	Tyr365	0.00	0.78
Ser366	0.00	0.60	Ser366	0.00	0.65	Ser366	0.00	0.61	Ser366	0.00	0.76
Val367	0.00	0.60	Val367	0.00	0.63	Val367	0.00	0.60	Val367	0.00	0.75
Leu368	0.00	0.82	Leu368	0.00	0.82	Leu368	0.00	0.64	Leu368	0.00	0.83
Tyr369	0.00	0.41	Tyr369	0.00	0.62	Tyr369	0.00	0.68	Tyr369	0.00	0.69
Asn370	0.00	0.94	Asn370	0.00	0.94	Asn370	0.00	0.91	Asn370	0.00	1.05
Ser371	0.00	0.72	Leu371	0.00	0.79	Phe371	0.00	0.65	Phe371	0.00	0.65
Ala372	0.00	0.70	Ala372	0.00	-0.40	Ala372	0.00	-0.54	Ala372	0.00	-0.74
Ser373	-0.01	0.79	Pro373	-0.01	0.17	Pro373	-0.01	0.45	Pro373	-0.01	0.48
Phe374	-0.01	-0.05	Phe374	-0.02	0.18	Phe374	-0.01	0.02	Phe374	-0.01	0.14
Ser375	-0.01	-0.38	Phe375	-0.07	-0.15	Phe375	-0.03	-0.33	Phe375	-0.11	-0.34
Thr376	-0.01	0.11	Thr376	-0.01	0.09	Ala376	-0.01	0.26	Ala376	-0.01	0.20
Phe377	-0.01	0.29	Phe377	-0.01	0.24	Phe377	0.00	0.18	Phe377	-0.01	0.04
Lys378	-0.01	-83.75	Lys378	-0.01	-80.26	Lys378	-0.01	-78.67	Lys378	-0.01	-77.94
Cyx379	0.00	0.50	Cyx379	0.00	0.45	Cyx379	0.00	0.36	Cyx379	0.00	0.43
Tyr380	0.00	-0.25	Tyr380	-0.01	-0.24	Tyr380	0.00	-0.27	Tyr380	0.00	-0.15
Gly381	0.00	-0.30	Gly381	0.00	-0.30	Gly381	0.00	-0.28	Gly381	0.00	-0.28
Val382	0.00	0.58	Val382	0.00	0.64	Val382	0.00	0.49	Val382	0.00	0.59
Ser383	0.00	-0.40	Ser383	0.00	-0.35	Ser383	0.00	-0.34	Ser383	0.00	-0.34
Pro384	0.00	-0.58	Pro384	0.00	-0.55	Pro384	0.00	-0.56	Pro384	0.00	-0.54
Thr385	0.00	-0.26	Thr385	0.00	-0.23	Thr385	0.00	-0.17	Thr385	0.00	-0.19
Lys386	0.00	-62.47	Lys386	0.00	-60.44	Lys386	0.00	-58.90	Lys386	0.00	-58.49
Leu387	0.00	-0.65	Leu387	0.00	-0.64	Leu387	0.00	-0.55	Leu387	0.00	-0.59
Asn388	0.00	-0.42	Asn388	0.00	-0.34	Asn388	0.00	-0.47	Asn388	0.00	-0.36
Asp389	0.00	58.12	Asp389	0.00	56.71	Asp389	0.00	55.39	Asp389	0.00	55.41
Leu390	0.00	-0.10	Leu390	0.00	0.02	Leu390	0.00	0.04	Leu390	0.00	0.18
Cyx391	0.00	-0.10	Cyx391	0.00	-0.03	Cyx391	0.00	-0.12	Cyx391	0.00	-0.08
Phe392	0.00	-0.37	Phe392	0.00	-0.38	Phe392	0.00	-0.21	Phe392	0.00	-0.33
Thr393	0.00	-0.12	Thr393	0.00	-0.15	Thr393	0.00	-0.03	Thr393	0.00	-0.08
Asn394	0.00	-0.14	Asn394	0.00	-0.26	Asn394	0.00	-0.05	Asn394	0.00	-0.20
Val395	0.00	-0.39	Val395	0.00	-0.38	Val395	0.00	-0.38	Val395	0.00	-0.31
Tyr396	0.00	0.11	Tyr396	0.00	0.08	Tyr396	0.00	0.13	Tyr396	0.00	0.04
Ala397	0.00	-0.32	Ala397	0.00	-0.27	Ala397	0.00	-0.26	Ala397	0.00	-0.22
Asp398	0.00	72.96	Asp398	0.00	71.83	Asp398	0.00	73.14	Asp398	0.00	70.76
Ser399	0.00	-0.41	Ser399	0.00	-0.22	Ser399	0.00	-0.32	Ser399	0.00	-0.14

Phe400	-0.01	-0.34	Phe400	-0.01	-0.50	Phe400	-0.01	-0.55	Phe400	-0.01	-0.57
Val401	-0.02	-0.41	Val401	-0.02	-0.28	Val401	-0.02	-0.07	Val401	-0.02	-0.30
Ile402	-0.04	-0.46	Ile402	-0.04	-0.44	Ile402	-0.03	-0.40	Ile402	-0.04	-0.26
Arg403	-0.53	-119.78	Arg403	-0.84	-118.03	Arg403	-0.58	-121.73	Arg403	-0.51	-114.92
Gly404	-0.04	-1.39	Gly404	-0.06	-1.23	Gly404	-0.04	-1.50	Gly404	-0.05	-1.49
Asp405	-0.22	111.74	Asp405	-0.49	100.57	Asn405	-0.27	-2.78	Asn405	-0.46	-1.92
Glu406	-0.11	107.52	Glu406	-0.18	103.76	Glu406	-0.13	106.85	Glu406	-0.12	99.43
Val407	-0.03	-1.22	Val407	-0.03	-0.95	Val407	-0.02	-1.17	Val407	-0.03	-1.19
Arg408	-0.14	-105.57	Arg408	-0.31	-98.08	Ser408	-0.04	-1.16	Ser408	-0.04	-1.17
Gln409	-0.05	-1.95	Gln409	-0.06	-1.55	Gln409	-0.07	-1.31	Gln409	-0.08	-1.25
Ile410	-0.01	-0.15	Ile410	-0.02	-0.23	Ile410	-0.01	-0.70	Ile410	-0.01	-0.25
Ala411	-0.01	0.33	Ala411	-0.01	0.45	Ala411	-0.01	-0.09	Ala411	-0.01	0.40
Pro412	0.00	0.87	Pro412	-0.01	0.85	Pro412	-0.01	-0.03	Pro412	0.00	0.62
Gly413	0.00	0.38	Gly413	0.00	0.29	Gly413	0.00	-0.05	Gly413	0.00	0.28
Gln414	-0.01	0.24	Gln414	-0.02	-0.16	Gln414	-0.01	0.15	Gln414	-0.01	0.15
Thr415	-0.02	0.02	Thr415	-0.03	0.21	Thr415	-0.03	0.47	Thr415	-0.02	-0.13
Gly416	-0.02	-1.52	Gly416	-0.04	-1.70	Gly416	-0.03	-0.86	Gly416	-0.02	-0.73
Lys417	-0.22	-122.99	Asn417	-0.27	-25.46	Asn417	-0.18	-2.22	Asn417	-0.20	-0.57
Ile418	-0.19	-1.22	Ile418	-0.07	-0.64	Ile418	-0.11	-0.40	Ile418	-0.24	-0.71
Ala419	-0.02	-0.96	Ala419	-0.02	-1.05	Ala419	-0.01	-0.39	Ala419	-0.02	-0.46
Asp420	-0.03	86.21	Asp420	-0.03	86.28	Asp420	-0.03	82.88	Asp420	-0.02	81.03
Tyr421	-0.15	-1.30	Tyr421	-0.16	-1.01	Tyr421	-0.19	-1.41	Tyr421	-0.12	-0.96
Asn422	-0.03	-1.76	Asn422	-0.03	-1.15	Asn422	-0.03	-0.73	Asn422	-0.02	-0.88
Tyr423	-0.01	0.94	Tyr423	-0.01	1.00	Tyr423	-0.01	0.05	Tyr423	-0.02	0.64
Lys424	-0.01	-81.14	Lys424	-0.01	-80.79	Lys424	-0.01	-79.15	Lys424	-0.01	-76.80
Leu425	-0.01	0.35	Leu425	-0.01	0.45	Leu425	-0.01	0.30	Leu425	0.00	0.33
Pro426	0.00	0.89	Pro426	0.00	0.82	Pro426	0.00	0.67	Pro426	0.00	0.77
Asp427	0.00	73.08	Asp427	0.00	71.86	Asp427	0.00	70.93	Asp427	0.00	67.94
Asp428	0.00	66.84	Asp428	0.00	65.58	Asp428	0.00	66.46	Asp428	0.00	62.60
Phe429	0.00	-0.04	Phe429	0.00	-0.06	Phe429	0.00	-0.09	Phe429	0.00	-0.13
Thr430	0.00	-0.21	Thr430	0.00	-0.18	Thr430	0.00	-0.07	Thr430	0.00	-0.17
Gly431	0.00	0.03	Gly431	0.00	0.03	Gly431	0.00	0.02	Gly431	0.00	-0.08
Cyx432	0.00	-0.58	Cyx432	0.00	-0.49	Cyx432	0.00	-0.43	Cyx432	0.00	-0.47
Val433	0.00	-0.24	Val433	-0.01	-0.31	Val433	0.00	-0.45	Val433	0.00	-0.33
Ile434	0.00	-0.04	Ile434	-0.01	0.04	Ile434	0.00	0.17	Ile434	0.00	0.07
Ala435	-0.01	-0.34	Ala435	-0.01	-0.37	Ala435	-0.01	-0.52	Ala435	-0.01	-0.47
Trp436	-0.02	-0.45	Trp436	-0.02	-0.23	Trp436	-0.01	0.00	Trp436	-0.02	-0.19
Asn437	-0.05	-1.56	Asn437	-0.05	-1.37	Asn437	-0.05	-1.08	Asn437	-0.04	-1.29
Ser438	-0.02	-0.35	Ser438	-0.02	-0.20	Ser438	-0.02	-0.16	Ser438	-0.02	0.09
Asn439	-0.10	-0.91	Asn439	-0.09	0.03	Asn439	-0.12	0.54	Asn439	-0.08	0.09
Asn440	-0.03	-0.33	Lys440	-0.04	-78.28	Lys440	-0.09	-100.21	Lys440	-0.04	-97.05
Leu441	-0.02	0.16	Leu441	-0.01	0.26	Leu441	-0.02	0.49	Leu441	-0.01	0.41
Asp442	-0.02	91.31	Asp442	-0.02	90.76	Asp442	-0.02	91.43	Asp442	-0.02	92.80
Ser443	-0.06	-1.29	Ser443	-0.05	-1.38	Ser443	-0.06	-0.02	Ser443	-0.05	-1.26
Lys444	-0.08	-93.78	Lys444	-0.08	-93.29	Lys444	-0.09	-93.83	Lys444	-0.09	-97.26
Val445	-0.18	-1.10	Val445	-0.18	-0.16	Val445	-0.35	-0.38	Val445	-0.37	-0.42
Gly446	-0.42	-0.12	Ser446	-0.32	0.96	Ser446	-0.95	-0.37	Ser446	-0.70	1.00
Gly447	-0.22	-0.82	Gly447	-0.15	-1.03	Gly447	-0.32	-0.93	Gly447	-0.18	-0.93

Asn448	-0.11	0.49	Asn448	-0.08	1.56	Asn448	-0.12	0.93	Asn448	-0.12	1.43
Tyr449	-0.45	-4.20	Tyr449	-0.35	-2.74	Tyr449	-1.12	-2.14	Tyr449	-0.37	-4.39
Asn450	-0.03	-0.73	Asn450	-0.03	-0.93	Asn450	-0.04	-0.89	Asn450	-0.03	-1.21
Tyr451	-0.05	1.00	Tyr451	-0.05	1.41	Tyr451	-0.05	1.63	Tyr451	-0.05	1.55
Leu452	-0.06	-0.69	Leu452	-0.06	-1.20	Leu452	-0.06	-1.09	Leu452	-0.06	-1.36
Tyr453	-0.74	-0.24	Tyr453	-0.82	0.33	Tyr453	-0.48	0.46	Tyr453	-0.55	0.58
Arg454	-0.10	-84.13	Arg454	-0.12	-89.27	Arg454	-0.10	-89.25	Arg454	-0.08	-87.06
Leu455	-2.64	1.52	Leu455	-2.55	0.04	Leu455	-2.22	-0.54	Leu455	-1.75	-0.93
Phe456	-2.47	-0.78	Phe456	-2.50	-0.68	Phe456	-1.37	0.44	Leu456	-1.12	-0.61
Arg457	-0.07	-79.28	Arg457	-0.08	-80.19	Arg457	-0.52	-91.45	Arg457	-0.06	-79.31
Lys458	-0.10	-84.55	Lys458	-0.11	-86.03	Lys458	-0.11	-85.90	Lys458	-0.09	-86.80
Ser459	-0.02	0.90	Ser459	-0.02	0.82	Ser459	-0.02	0.85	Ser459	-0.01	1.14
Asn460	-0.01	-1.01	Asn460	-0.02	-1.25	Asn460	-0.02	-0.71	Asn460	-0.01	-0.78
Leu461	-0.01	0.16	Leu461	-0.01	0.10	Leu461	-0.01	-0.30	Leu461	-0.01	-0.11
Lys462	0.00	-68.08	Lys462	0.00	-68.27	Lys462	0.00	-69.74	Lys462	0.00	-65.58
Pro463	0.00	-0.68	Pro463	0.00	-0.62	Pro463	0.00	0.05	Pro463	0.00	-0.57
Phe464	0.00	-0.74	Phe464	0.00	-0.83	Phe464	0.00	-0.20	Phe464	0.00	-0.65
Glu465	0.00	71.76	Glu465	0.00	72.99	Glu465	-0.01	72.43	Glu465	0.00	70.61
Arg466	0.00	-73.10	Arg466	-0.01	-74.52	Arg466	-0.01	-74.38	Arg466	0.00	-73.92
Asp467	-0.01	77.95	Asp467	-0.01	81.95	Asp467	-0.01	79.20	Asp467	-0.01	80.09
Ile468	-0.01	0.46	Ile468	-0.01	0.20	Ile468	-0.01	0.34	Ile468	-0.01	0.47
Ser469	-0.01	0.52	Ser469	-0.01	0.05	Ser469	-0.01	0.54	Ser469	-0.01	0.71
Thr470	-0.01	-0.16	Thr470	-0.01	-0.34	Thr470	-0.01	-0.47	Thr470	-0.01	-0.10
Glu471	-0.03	80.31	Glu471	-0.03	85.34	Glu471	-0.04	87.46	Glu471	-0.03	82.76
Ile472	-0.06	-0.73	Ile472	-0.06	-1.25	Ile472	-0.07	-1.37	Ile472	-0.07	-1.34
Tyr473	-0.91	-1.15	Tyr473	-1.00	-0.56	Tyr473	-1.08	0.15	Tyr473	-0.91	-0.77
Gln474	-0.29	-0.14	Gln474	-0.38	-0.68	Gln474	-1.59	-2.83	Gln474	-0.36	-0.53
Ala475	-2.37	-0.64	Ala475	-2.12	-1.71	Ala475	-2.48	-2.01	Ala475	-2.25	-1.16
Gly476	-1.21	-0.36	Gly476	-1.15	-0.55	Gly476	-1.43	-1.19	Gly476	-1.13	-0.39
Ser477	-0.61	-0.83	Asn477	-0.77	-1.13	Asn477	-1.77	-2.24	Asn477	-0.65	-1.12
Thr478	-0.21	0.42	Lys478	-0.41	-73.09	Lys478	-0.75	-90.02	Lys478	-0.27	-79.95
Pro479	-0.05	0.60	Pro479	-0.07	0.80	Pro479	-0.18	0.95	Pro479	-0.04	0.65
Cyx480	-0.05	-0.67	Cyx480	-0.06	-0.65	Cyx480	-0.12	-0.15	Cyx480	-0.05	-0.63
Asn481	-0.04	-0.36	Asn481	-0.05	0.29	Asn481	-0.20	0.39	Asn481	-0.03	0.03
Gly482	-0.03	-0.09	Gly482	-0.03	0.23	Gly482	-0.21	-0.49	Gly482	-0.03	-0.32
Val483	-0.15	0.22	Val483	-0.18	0.19	Val483	-1.04	-0.03	Val483	-0.18	-0.24
Glu484	-0.44	91.59	Ala484	-0.31	15.81	Ala484	-0.76	-0.62	Ala484	-0.31	-0.97
Gly485	-1.09	-0.60	Gly485	-0.64	-0.03	Gly485	-0.63	0.58	Gly485	-1.33	-0.94
Phe486	-4.97	-3.67	Phe486	-3.45	-1.82	Phe486	-4.80	-3.07	Phe486	-5.38	-4.32
Asn487	-1.48	-0.41	Asn487	-1.85	-2.70	Asn487	-1.45	-1.39	Asn487	-1.46	-1.37
Cyx488	-0.27	0.21	Cyx488	-0.24	0.41	Cyx488	-0.40	0.65	Cyx488	-0.31	0.36
Tyr489	-4.36	-0.95	Tyr489	-4.01	-3.18	Tyr489	-3.38	-4.66	Tyr489	-4.79	-3.52
Phe490	-0.61	-0.10	Phe490	-0.51	1.31	Phe490	-0.89	1.43	Phe490	-0.52	0.78
Pro491	-0.20	1.39	Pro491	-0.21	1.30	Pro491	-0.20	1.54	Pro491	-0.22	1.15
Leu492	-0.19	-1.83	Leu492	-0.21	1.01	Leu492	-0.22	1.21	Leu492	-0.23	1.63
Gln493	-2.03	-11.37	Arg493	-2.18	-137.71	Arg493	-1.37	-149.76	Arg493	-2.70	-152.08
Ser494	-0.36	2.21	Ser494	-0.36	3.01	Ser494	-0.29	1.24	Ser494	-0.50	2.17
Tyr495	-0.34	-2.54	Tyr495	-0.47	-2.30	Tyr495	-0.46	-2.81	Tyr495	-0.40	-1.31

Gly496	-0.54	-2.18	Ser496	-0.47	-6.00	Ser496	-0.51	-3.90	Ser496	-0.56	-4.80
Phe497	-0.41	-0.53	Phe497	-0.31	-0.29	Phe497	-0.23	0.06	Phe497	-0.24	-0.12
Gln498	-2.11	-4.18	Arg498	-2.39	-117.37	Arg498	-2.17	-147.73	Arg498	-2.45	-153.32
Pro499	-0.37	0.71	Pro499	-0.33	0.07	Pro499	-0.63	0.54	Pro499	-0.33	0.08
Thr500	-2.68	-3.45	Thr500	-2.57	-3.27	Thr500	-2.00	-3.73	Thr500	-2.50	-3.86
Asn501	-3.19	-6.80	Tyr501	-4.66	-5.69	Tyr501	-4.21	-5.50	Tyr501	-4.60	-4.24
Gly502	-1.59	-4.29	Gly502	-1.32	-2.62	Gly502	-1.02	-1.15	Gly502	-1.11	-1.78
Val503	-1.64	-1.31	Val503	-1.67	0.08	Val503	-0.86	-0.55	Val503	-0.84	-0.80
Gly504	-0.40	-2.20	Gly504	-0.37	-0.85	Gly504	-0.30	-1.42	Gly504	-0.31	-1.47
Tyr505	-3.82	-5.85	Hid505	-3.90	-2.69	Hid505	-2.32	-2.74	Hid505	-2.82	-3.24
Gln506	-0.33	-2.58	Gln506	-0.32	-2.35	Gln506	-0.28	-1.57	Gln506	-0.28	-3.37
Pro507	-0.07	0.63	Pro507	-0.07	0.19	Pro507	-0.05	0.06	Pro507	-0.06	0.25
Tyr508	-0.07	0.14	Tyr508	-0.07	0.51	Tyr508	-0.05	0.63	Tyr508	-0.07	0.59
Arg509	-0.01	-85.19	Arg509	-0.01	-83.77	Arg509	-0.01	-84.38	Arg509	-0.01	-85.26
Val510	-0.01	-0.08	Val510	-0.01	0.02	Val510	-0.01	0.16	Val510	-0.01	0.12
Val511	0.00	0.50	Val511	0.00	0.43	Val511	0.00	0.41	Val511	0.00	0.39
Val512	0.00	-0.29	Val512	0.00	-0.22	Val512	0.00	-0.21	Val512	0.00	-0.16
Leu513	0.00	0.34	Leu513	0.00	0.32	Leu513	0.00	0.24	Leu513	0.00	0.26
Ser514	0.00	-0.26	Ser514	0.00	-0.25	Ser514	0.00	-0.22	Ser514	0.00	-0.23
Phe515	0.00	0.26	Phe515	0.00	0.26	Phe515	0.00	0.31	Phe515	0.00	0.17
Glu516	0.00	61.74	Glu516	0.00	60.75	Glu516	0.00	62.10	Glu516	0.00	59.21
Leu517	0.00	-0.07	Leu517	0.00	0.03	Leu517	0.00	-0.13	Leu517	0.00	-0.02
Leu518	0.00	-0.19	Leu518	0.00	-0.05	Leu518	0.00	-0.09	Leu518	0.00	-0.20
Hid519	0.00	-0.21	Hid519	0.00	-0.29	Hid519	0.00	-0.03	Hid519	0.00	0.06
Ala520	0.00	0.23	Ala520	0.00	0.28	Ala520	0.00	0.10	Ala520	0.00	0.08
Pro521	0.00	0.44	Pro521	0.00	0.45	Pro521	0.00	0.21	Pro521	0.00	0.39
Ala522	0.00	-0.39	Ala522	0.00	-0.43	Ala522	0.00	0.06	Ala522	0.00	-0.38
Thr523	0.00	-0.03	Thr523	0.00	-0.02	Thr523	0.00	0.07	Thr523	0.00	0.01
Val524	0.00	-0.05	Val524	0.00	-0.07	Val524	0.00	0.15	Val524	0.00	-0.11
Cyx525	0.00	-0.02	Cyx525	0.00	0.00	Cyx525	0.00	-0.09	Cyx525	0.00	0.03
Gly526	0.00	57.15	Gly526	0.00	56.30	Gly526	0.00	53.71	Gly526	0.00	55.70
